# Machine-Learning-Derived Nomogram Based on 3D Radiomic Features and Clinical Factors Predicts Progression-Free Survival in Lung Adenocarcinoma

**DOI:** 10.3389/fonc.2021.692329

**Published:** 2021-06-23

**Authors:** Guixue Liu, Zhihan Xu, Yaping Zhang, Beibei Jiang, Lu Zhang, Lingyun Wang, Geertruida H. de Bock, Rozemarijn Vliegenthart, Xueqian Xie

**Affiliations:** ^1^ Department of Radiology, Shanghai General Hospital, Shanghai Jiao Tong University School of Medicine, Shanghai, China; ^2^ DI CT Collaboration, Siemens Healthineers Ltd., Shanghai, China; ^3^ Department of Epidemiology, Hanzeplein 1, University Medical Center Groningen, University of Groningen, Groningen, Netherlands; ^4^ Department of Radiology, Hanzeplein 1, University Medical Center Groningen, University of Groningen, Groningen, Netherlands

**Keywords:** radiomics, machine learning, progression-free survival, lung adenocarcinoma, computed tomography

## Abstract

**Background:**

To establish a machine-learning-derived nomogram based on radiomic features and clinical factors to predict post-surgical 2-year progression-free survival (PFS) in patients with lung adenocarcinoma.

**Methods:**

Patients with >2 years post-surgical prognosis results of lung adenocarcinoma were included in Hospital-1 for model training (n = 100) and internal validation (n = 50), and in Hospital-2 for external testing (n = 50). A total of 1,672 radiomic features were extracted from 3D segmented CT images. The Rad-score was established using random survival forest by accumulating and weighting the top-20 imaging features contributive to PFS. A nomogram for predicting PFS was established, which comprised the Rad-score and clinical factors highly relevant to PFS.

**Results:**

In the training, internal validation, and external test groups, 69/100 (69%), 37/50 (74%) and 36/50 (72%) patients were progression-free at two years, respectively. According to the Rad-score, the integral of area under the curve (iAUC) for discriminating high and low risk of progression was 0.92 (95%CI: 0.77-1.0), 0.70 (0.41-0.98) and 0.90 (0.65-1.0), respectively. The C-index of Rad-score was 0.781 and 0.860 in the training and external test groups, higher than 0.707 and 0.606 for TNM stage, respectively. The nomogram integrating Rad-score and clinical factors (lung nodule type, cM stage and histological type) achieved a C-index of 0.845 and 0.837 to predict 2-year PFS, respectively, significantly higher than by only radiomic features (all p < 0.01).

**Conclusion:**

The nomogram comprising CT-derived radiomic features and risk factors showed a high performance in predicting post-surgical 2-year PFS of patients with lung adenocarcinoma, which may help personalize the treatment decisions.

## Introduction

Lung cancer is the most common malignant tumor worldwide, accounting for 11.6% of all cancers, and 18.4% of all cancer deaths (1). Population-based screening improved the detection of early-stage lung cancer (2). TNM stage, as determined by medical imaging, has been widely considered an important predictor of prognosis and is used to guide therapeutic decision-making (3). However, even patients with the same TNM stage may have different prognoses due to tumor heterogeneity (4). In lung adenocarcinoma, the primary histological type of lung cancer, post-surgical outcomes vary among patients, and post-surgical recurrence is frequent because most cases have mixed subtypes (5), increasing the importance of a personalized post-surgical follow-up theme.

Machine learning algorithms have been developed to analyze the high-dimensional features of tumor images, which provide better specificity than naked-eye observation (6). Radiomics is a subfield of machine learning, in which interpretable quantitative features are extracted from medical images to characterize tumor heterogeneity. Tumor radiomics research has made some progress, such as predicting survival rate after chemotherapy (7) and immunotherapy (8), analyzing the tumor microenvironment (9), and promoting individualized treatment of patients by providing accurate and effective decision support (10). Previous studies have associated radiomic features with multiple clinical endpoints, such as survival and drug response, in patients with non-small cell lung cancer (NSCLC) (11–13). However, the concordance index of the radiomic models for predicting the survival in lung cancer was not optimal (11). There is no consensus whether radiomics-based methods can effectively predict the post-surgical prognosis of patients with lung adenocarcinoma.

Selection of appropriate follow-up plans after resection of lung adenocarcinoma requires accurate prediction of post-surgical progression-free survival (PFS) according to tumor characteristics and histological subtypes. The purpose of this study was to train and test a machine-learning-derived radiomics approach to predict the PFS of post-surgical patients with lung adenocarcinoma, and establish a nomogram based on radiomic score and multiple clinical PFS-related factors.

## Materials and Methods

### Study Sample

We searched the electronic health records of two medical centers. In the first center (Hospital-1, Shanghai General Hospital – North [city center]), 100 patients admitted from July 2017 to June 2018 were randomly selected as the training cohort, and 50 patients from July 2018 to December 2018 were randomly selected as the internal validation cohort. In the second center (Hospital-2, Shanghai General Hospital – South [Songjiang new city]), 50 patients from July 2016 to December 2018 were randomly selected for external testing. The inclusion criteria were: 1) patients whose lung tumor tissue was completely resected by surgical operation; 2) lung adenocarcinoma diagnosed based on hematoxylin-eosin gross pathological specimen and immunohistochemical staining; 3) ≥ 2-year follow-up to obtain PFS results (or shorter in case of earlier progression); 4) with pre-surgical thin-slice (<1mm) contrast-enhanced CT scanning; 5) ≤ 2 months interval between CT scan and surgical resection.

The collected baseline data were age, gender, smoking status (non-smoker or smoker), 8^th^ edition cTNM stage, lung nodule type (solid or subsolid), histological subtype, and post-surgical treatment. The cTNM stage was determined by whole-body positron emission tomography (PET)-CT or whole-body CT for the head, neck, chest and abdomen, except the lower extremities. The histological subtype of adenocarcinoma was defined by the 2015 World Health Organization classification of lung cancer (14). The 150 patients in Hospital-1 underwent molecular testing for *EGFR* mutations (expression of exon-18, -19, -20, and -21) using the human gene mutation detection kit (Aide Biomedical Technology).

The study endpoint was PFS, defined as the period from tumor resection to tumor recurrence observed by PET-CT or whole-body CT, or death for any reason. The PFS results were obtained from patient medical records, including chest CT scans, clinical outcomes, or death records.

**Figure 1:**
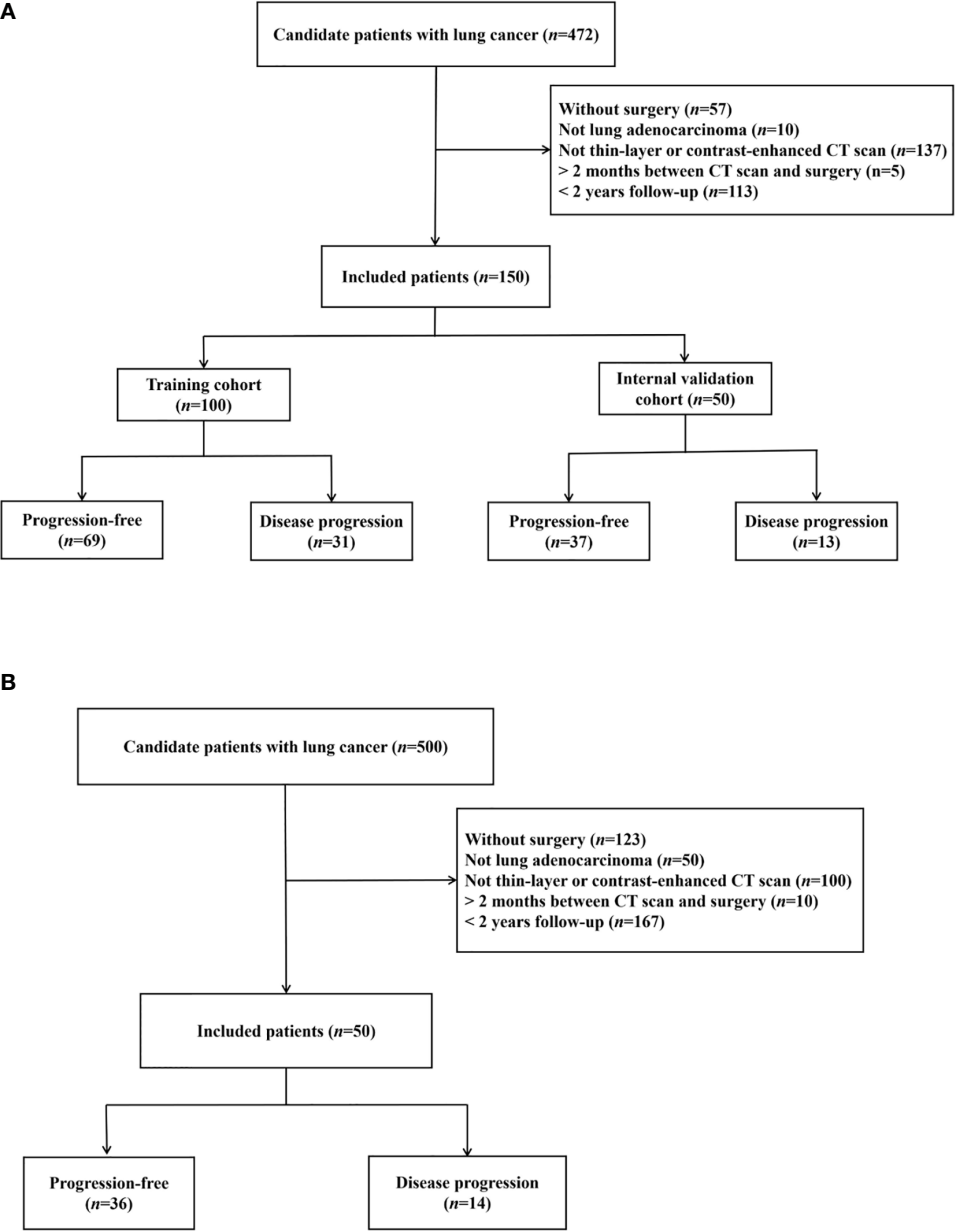
Patient inclusion flowcharts in two hospitals. **(A)** Hospital-1 (training and internal validation). **(B)** Hospital-2 (external testing).

**Figure 2:**
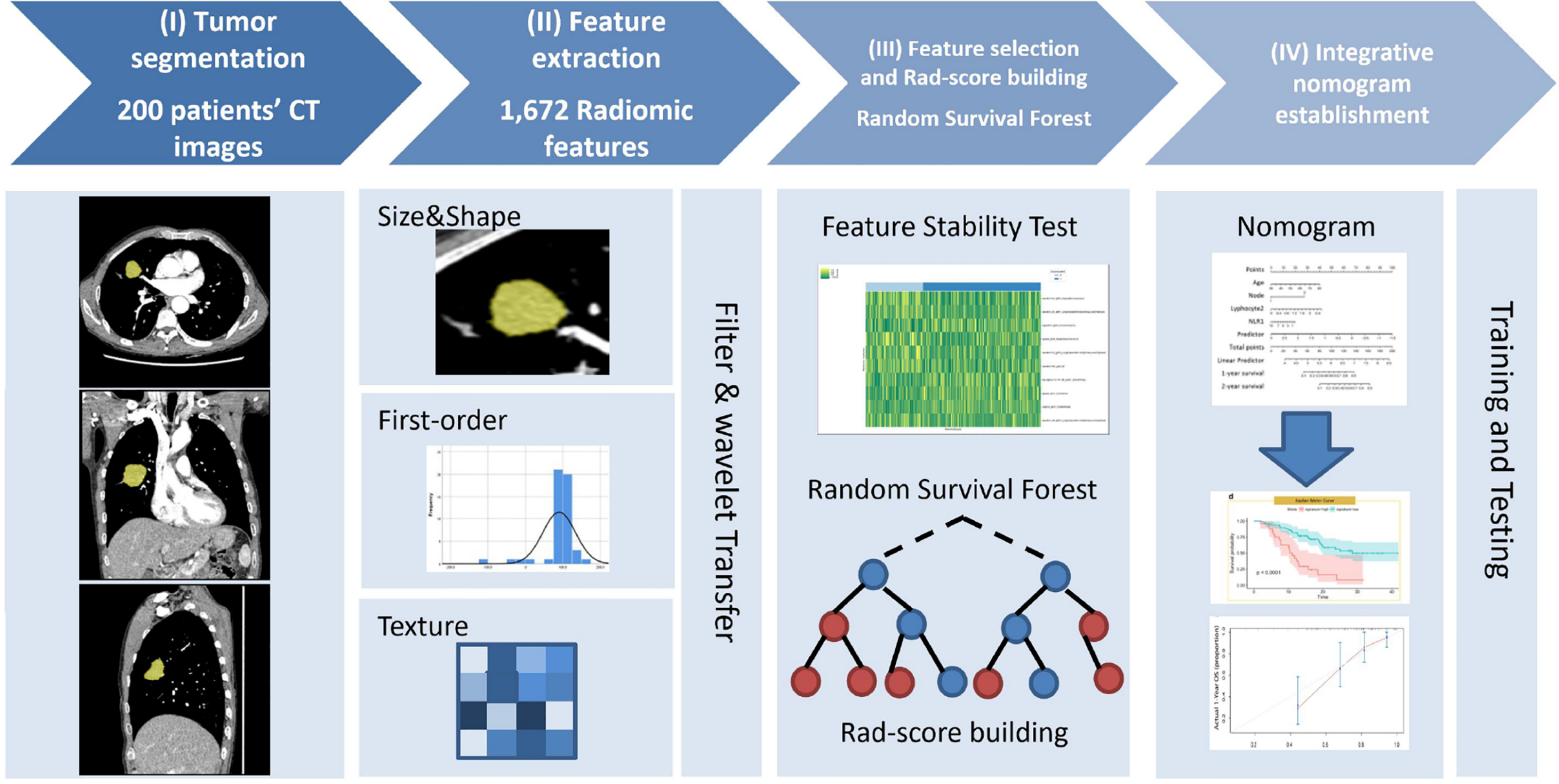
Study workflow diagram. (I) Tumor segmentation: 3D semi-automatic segmentation of tumors on chest CT images. (II) Feature extraction: 1,672 radiomic features were automatically extracted from the segmented tumor volume. (III) Feature selection and Rad-score calculation: Spearman’s rank correlation was used to select reproductive and stable radiomic features, and random survival forest model was implemented to calculate Rad-score. (IV) Nomogram development: the model was trained and tested to determine its predictive performance.

The institutional review boards approved this retrospective study and waived the requirement for patient informed consent in the two hospitals. **Figure 1** shows the patient selection flow diagram. **Figure 2** shows the study workflow.

### CT Acquisition

Various CT scanners of different type were used in clinical practice, including three CT systems (Somatom Force, Siemens Healthineers; Revolution and HD750, GE Healthcare) in Hospital-1, and two CT systems (Somatom Flash, Siemens Healthineers; Revolution, GE Healthcare) in Hospital-2. All study patients underwent contrast-enhanced chest CT scanning after injection of 60-80 mL contrast-media (Iopamiro 300, Bracco) into the antecubital vein at 3-4 mL/sec. The image slice thickness was 0.6 mm or 0.625 mm. **Supplementary Table 1** details the acquisition protocol and reconstruction parameters.

### Tumor Segmentation and Radiomic Feature Extraction

A dedicated software package (Radiomics v1.2.3, Siemens Healthineers) running on a research platform (SyngoVia VB10, Research Frontier, Siemens Healthineers) was used to perform three-dimensional tumor segmentation and feature extraction. This package was developed based on the PyRadiomics library (http://www.radiomics.io) and subjected to the Image Biomarker Standardization Initiative (15). One radiologist with 6 years of experience in thoracic imaging semi-automatically segmented the tumor lesions on CT images by finding the lesion and clicking on it, blinded to the prognostic results. Then the software automatically extracted a total of 1,672 radiomic features for each lesion, including first-order, size and shape, and texture features. **Supplementary Table 2** describes these features.

### Feature Selection

Radiomic features with good stability and repeatability can be selected as candidates for further feature selection. To select these candidate features, 50 patients were randomly chosen from the training cohort. Two radiologists with 20 and 6 years of experience independently segmented the tumor lesions of these 50 patients. Spearman’s rank correlation coefficients between the 1,672 features measured by the two radiologists were calculated to filter unstable features (16). The radiomic features with a Spearman’s r > 0.8 were considered stable and reproducible, and were chosen to build the predictive model (17).

### Model Establishment

A machine-learning method, the random survival forest was used to establish the R-model and generate a Rad-score. Random survival forest provides high predictive accuracy with nonlinear regression. It is a robust algorithm with an automated feature screening process suitable for right-censored correlated complex survival datasets compared with the commonly used least-absolute shrinkage and selection operator (LASSO)-Cox regression (18). Random survival forest is suitable for integrating high-dimensional features like radiomics features for survival analysis and risk stratification. The random survival forest model was recently used to identify risk factors and generate radiomic signatures for different diseases (19). The robustness of the R-model was validated by a five-fold cross-validation approach for tuning the optimal hyperparameters. Feature importance was evaluated by the magnitude of the log-rank test statistic by permutation (20). Accordingly, the Rad-score (range from 0 to 1) was computed and generated by the random survival forest model to represent the average of expected number of events across all trees in the established forest (21). A higher Rad-score represented a higher risk of progression or shorter PFS.

A Kaplan-Meier curve with log-rank test and univariate Cox proportional-hazards model was applied to select candidate influential factors (clinical factors and *EGFR* mutation status) and establish a C-model. Factors with a p-value < 0.1 were included in the multivariate Cox proportional-hazards regression to establish the C-model. Prognosis prediction based on TNM staging was evaluated.

To establish the Combi-model and avoid multicollinearity and potential correlation between the Rad-score and clinical factors, a multivariate Cox proportional-hazards model with stepwise selection using minimum Akaike information criterion was implemented to integrate the Rad-score and high-relevance clinical risk factors.

### Solid Tumor *vs.* Subsolid Tumor

Subgroup stratified analysis was implemented to verify that the radiomics model can independently predict prognosis in patients with solid and subsolid tumors. The patients in each group were classified by Rad-score as high or low risk of disease progression. The prognosis difference between risk levels was assessed in each group by Kaplan-Meier survival analysis with the log-rank test.

### Statistical Analysis

Clinical characteristics between datasets were compared using independent sample *t*-test or Wilcoxon test for continuous variables depending on the normality test, whereas Chi-square or Fisher’s exact test was used for categorical variables.

The R-score’s predictive ability was evaluated by Harrell’s concordance index (C-index) in the training, internal validation, and external test cohorts. The risk stratification capability was assessed by using the Kaplan-Meier survival curve and log-rank test. The optimal R-score cut-off values were determined by X-tile software (version 3.6.1, Yale University) (22) in the training cohort and applied to the internal validation and external test cohorts.

The proportional-hazards assumption was first verified by the Schoenfeld residuals test to evaluate model performance. Time-dependent receiver operating characteristic (ROC) curves, the integral of the area under the curves (iAUC) at various timepoints and C-index were generated and calculated in the training and internal validation cohorts to compare the prognosis discriminative ability of the three models (R-, C- and Combi-). The goodness-fit of all the three models was illustrated by the calibration curve by calculating the actual and predicted probabilities of PFS at 2 year. Decision curve analysis (DCA) was used to evaluate and compare the three models incorporating clinical net benefits. Finally, a nomogram of the Combi-model was built to visually represent the final predictive model making it convenient for clinicians to identify new patients.

Statistical analysis was performed with Python Scikit-survival v0.13.2 (https://scikit-survival.readthedocs.io), Lifelines library v0.25.5 (https://lifelines.readthedocs.io), and R package v3.6.0 (http://www.r-project.org). Detailed descriptions of the software packages and functions are listed in the Supplementary Methods. All statistical tests were two-sided, and statistical significance was set at p<0.05.

## Results

### Patient Characteristics

The training, internal validation and external test groups comprised 150 (61.8 ± 8.6 years old), 50 (60.8 ± 9.7), and 50 (59.9 ± 10.3) patients, respectively. All patients had histologically proven lung adenocarcinoma. Whole-body PET-CT was performed for 51/100 (51%) training, 23/50 (46%) internal validation, and 35/50 (70%) external test patients. The remaining patients underwent whole-body CT except for the lower extremities. At two years, 69/100 (69%) training, 37/50 (74%) internal validation, and 36/50 (72%) external test patients were progression-free. PFS was 409.1 ± 202.1 days, 413.8 ± 188.8 days and 541.8 ± 364.7 days, respectively. **Table 1** shows the association between patient characteristics and PFS.

**Table 1:** Association between patient characteristics and progression-free survival.

Characteristics	Hospital-1		P_1_ value	Hospital-2	P_2_ value
	Training (*n*=100)	Internal validation (*n*=50)		External test (*n*=50)	
Age (mean ± SD)	61.8 ± 8.6	60.8 ± 9.7	1.000	59.9 ± 10.3	0.389
Sex			0.863		0.602
Male	52 (52%)	27 (54%)		29 (58%)	
Female	48 (48%)	23 (46%)		21 (42%)	
Smoking Status			1.000		0.090
Yes	11 (11%)	6 (12%)		11 (22%)	
No	89 (89%)	44 (88%)		39 (78%)	
Lung nodule type			0.489		0.002*
Solid	45 (45%)	26 (52%)		39 (78%)	
Progression	6 (6%)	1 (2%)		13 (26%)	
Progression-free	39 (39%)	25 (50%)		26 (52%)	
Subsolid	55 (55%)	24 (48%)		11 (22%)	
Progression	25 (25%)	12 (24%)		0 (0%)	
Progression-free	30 (30%)	12 (24%)		11 (22%)	
Lesion location			0.140		0.624
Right upper lobe	40 (40%)	14 (28%)		14 (28%)	
Right middle lobe	7 (7%)	4 (8%)		7 (14%)	
Right lower lobe	13 (13%)	10 (20%)		10 (20%)	
Left upper lobe	20 (20%)	17 (34%)		10 (20%)	
Left lower lobe	20 (20%)	5 (10%)		9 (18%)	
Histological type			0.493		0.100
Invasive adenocarcinoma	75 (75%)	40 (80%)		30 (60%)	
Microinvasive adenocarcinoma	17 (17%)	5 (10%)		0	
Other subtypes	8 (8%)	5 (10%)		20 (40%)	
cT category			0.160		0.460
T1	47 (47%)	29 (58%)		26 (52%)	
T2	15 (15%)	2 (4%)		11 (22%)	
T3	13 (13%)	8 (16%)		4 (8%)	
T4	25 (25%)	11 (22%)		9 (18%)	
cN category			0.486		0.175
N0	57 (57%)	24 (48%)		38 (76%)	
N1	19 (19%)	14 (28%)		6 (12%)	
N2	19 (19%)	11 (22%)		5 (10%)	
N3	5 (5%)	1 (2%)		1 (2%)	
cM category			0.477		0.011*
M0	66 (66%)	30 (60%)		43 (86%)	
M1	34 (34%)	20 (40%)		7 (14%)	
Overall stage					0.12
Stage I	28 (28%)	15 (30%)		28 (56%)	
Stage II	13 (13%)	6 (12%)		8 (16%)	
Stage III	25 (25%)	9 (18%)		7 (14%)	
Stage IV	34 (34%)	20 (40%)		7 (14%)	
*EGFR* mutation			0.492		\
Exon19del expression	24 (24%)	12 (24%)		\	
Exon21L expression	38 (38%)	22 (44%)		\	
Exon18 expression	2 (2%)	1 (2%)		\	
Exon20 expression	1 (1%)	2 (4%)		\	
Wild type	35 (35%)	13 (26%)			
Post-surgical treatment			0.291		0.101
Yes	44 (44%)	17 (34%)		32 (64%)	
No	56 (56%)	33 (66%)		18 (36%)	

Data are represented as mean ± standard deviation or number (percentage). P_1_-value represents the significance between the training and internal validation cohorts. P_2_-value represents the significance between the training and external test cohorts. * indicates p < 0.05. Other histological subtypes of lung adenocarcinoma: in the training cohort, 3 patients had adenocarcinoma in situ, 4 had unclassified adenocarcinoma, and 1 had solid adenocarcinoma with mucus secretion; in the internal validation cohort, 2 patients had adenocarcinoma in situ and 3 had unclassified adenocarcinoma; in the external test cohort, 1 patient had poorly differentiated adenocarcinoma, 2 had adenocarcinoma in situ, 15 had unclassified adenocarcinoma, 1 had poorly differentiated adenocarcinoma and 1 had pleomorphic carcinoma with adenocarcinoma. Disease progression: in the training cohort, 30 patients recurred and 1 case died; in the internal validation cohort, 12 patients recurred and 1 case died; in the external test cohort, 13 patients recurred and 1 case died. Post-surgical treatment: postoperative radiotherapy, chemotherapy and targeted therapy. EGFR, epidermal growth factor receptor; SD, standard deviation.

### Establishment and Assessment of the Radiomics Model

The feature stability analysis yielded 597 radiomic features out of 1,672 with a Spearman’s *r* > 0.8 (**Supplementary Table 2**), that were considered stable features indicating good interobserver consistency. From these stable features, the 20 features highly contributive to PFS were selected to create an R-score using a random survival forest algorithm (**Supplementary Figure 3**). The optimal R-score cut-off value to discriminate high and low progression risk was set to 3.684 based on the training cohort determined by X-tile (22), and the patients were subsequently stratified as high or low risk for disease progression (**Supplementary Figure 4**). Kaplan-Meier analysis showed that this cut-off value could discriminate high- and low-risk patients in the training (p<0.001), internal validationt (p=0.0014), and external test (p=0.045) cohorts.

**Figure 3:**
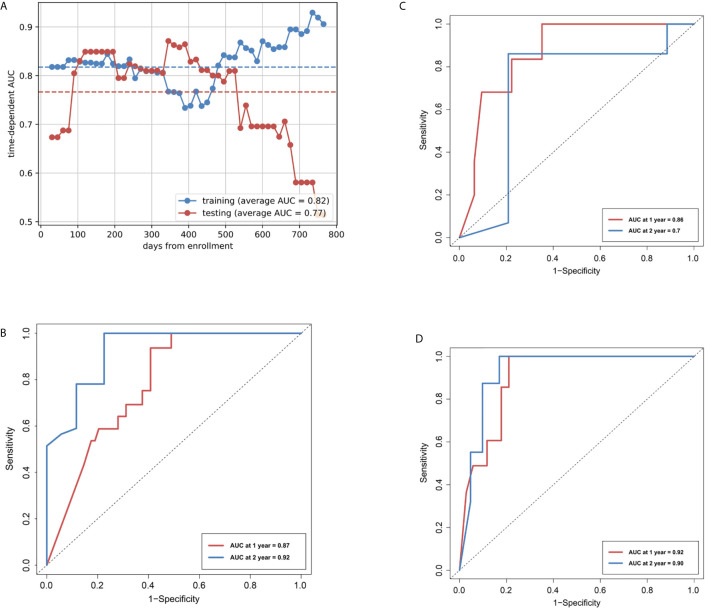
Area under the receiver operating characteristic curves (AUCs) of R-model. **(A)** AUCs of Rad-score over time in the training and internal validation cohorts. **(B)** Time-dependent ROCs in the training cohort. **(C)** Time-dependent ROCs in the internal test cohort. **(D)** Time-dependent ROCs in the external test cohort.

In the training cohort, the R-model reached an average AUC of 0.82 for discriminating patients with high- and low-risk for disease progression (**Figure 3A**), and an iAUC of 0.78 (95%CI: 0.67 to 0.87) and 0.92 (0.77 to 1.0) at one and two years, respectively (**Figure 3B**). In the internal validation cohort, R-model reached an average AUC of 0.77, and iAUC of 0.86 (0.74 to 0.99) and 0.70 (0.41 to 0.98) at one and two years, respectively (**Figure 3C**). In the external test cohort, the R-model showed an iAUC of 0.92 (0.70 to 0.98) and 0.90 (0.69 to 1.0) at one and two years, respectively (**Figure 3D**).

For the training and external test cohorts, the C-index was 0.781 and 0.778, respectively. All the C-indexes were higher than 0.7, indicating good predictive performance. The calibration curves showed visually good agreement between the predicted and actual labels in the training, internal validation and external test cohorts (**Supplementary Figure 5**).

### Establishment and Assessment of Clinical Factor Model

Kaplan-Meier survival analysis showed that lung nodule type and cM stage were significantly associated with PFS. The PFS was significantly shorter for patients with solid tumors than those with subsolid tumors (p=0.002), and patients with distant metastasis than those without (p=0.011). **Supplementary Figure 1** shows the corresponding Kaplan-Meier curves.

The univariate Cox regression analysis showed that sex, lung nodule type (subsolid or solid), cT, cN, and cM stage were significantly associated with PFS (all p<0.1) (**Supplementary Table 3**), so they were included for multivariate regression analysis to establish the C-model. The C-index based on TNM stage was 0.707 and 0.606 for the training and external test cohorts, respectively (**Supplementary Figure 6**).

**Figure 4:**
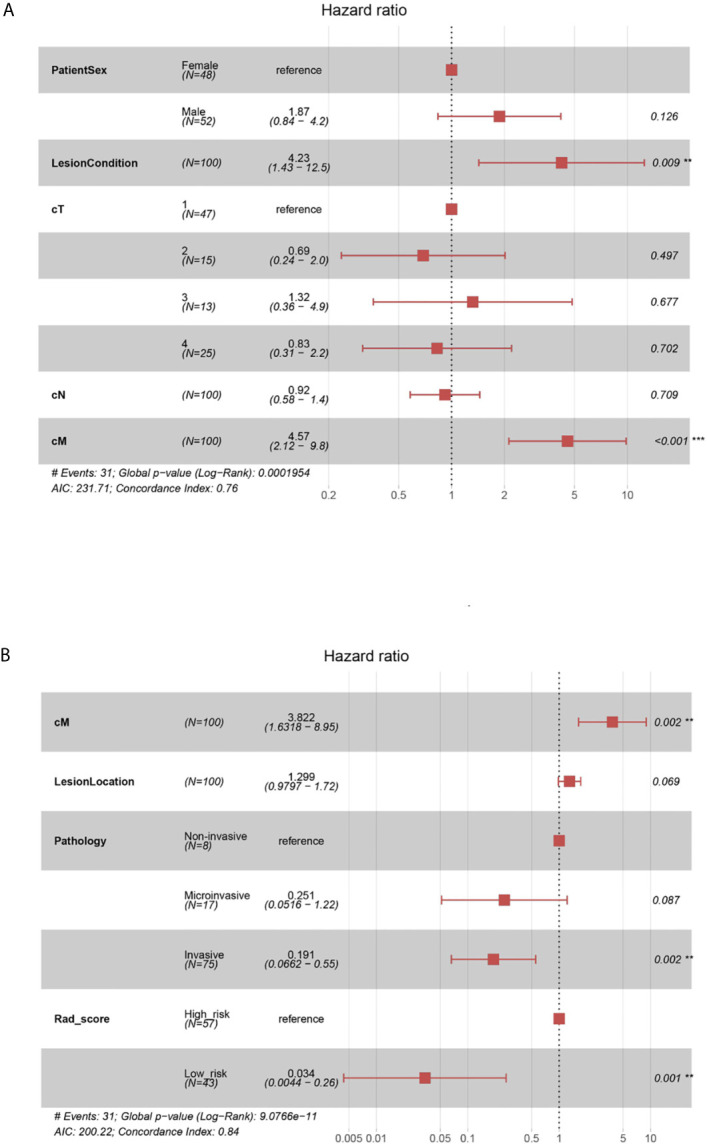
**(A)** Forest plots of C-model. The forest plots of hazard ratio of each selected clinical factor in C-model, established from the training cohort. The likelihood ratio test was used to calculate P-value. **(B)** Forest plots of Combi-model. The forest plots showed each selected clinical factor’s hazard ratio in the combined model, established from the training cohort.

The global Schoenfeld test (p=0.645) showed that the C-model met the proportional hazard assumption requirement (**Supplementary Figure 7**). **Figure 4A** shows the forest plots of the C-model. The calibration curves showed visually good agreement between the predicted and actual labels in the training and validation cohorts (**Supplementary Figure 8**). The C-indexes were 0.755 and 0.739 for the training and internal validation cohorts, respectively. In the multivariate regression analysis, lung nodule type (HR=4.23, p=0.009) and cM stage (HR=4.57, p<0.001) were significantly correlated with PFS, thus were included in the Combi-model.

Schoenfeld individual tests also showed the genetic factors model adhered to the proportional hazard assumption requirement (**Supplementary Figure 9**). The C-indexes of *EGFR* mutations were 0.612 and 0.528 for the training and validation cohorts, respectively. Because of these insignificant results, the genetic factor was not integrated into the Combi-model.

### Establishment and Assessment of the Combined Model

**Figure 5:**
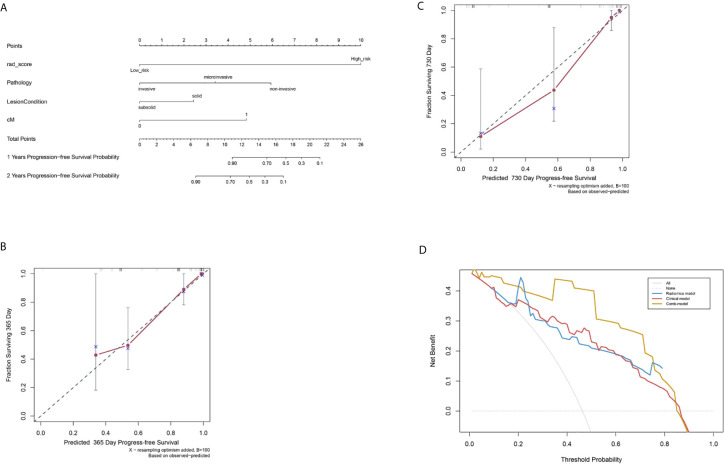
Nomogram to evaluate post-surgical progression-free survival (PFS) of patients with lung adenocarcinoma, and model calibration. **(A)** A nomogram to predict 1- and 2-year PFS probability. The Rad-score was located on the Rad-score axis, where a line was drawn to indicate the PFS probability point scale. After repeating this process for each variable and accumulating all the probability points, the final PFS probability can be established and indicated on the axis line. **(B)** Calibration curve for the estimation of 1-year overall survival predicted by the nomogram. The estimated progress-free rate was plotted on the *x*-axis; the observed progress-free rate was plotted on the *y*-axis. The Diagonal dotted line = a perfect estimation by an ideal model; solid line = the performance of the nomogram. **(C)** Calibration curve for the estimation of 2-year overall survival predicted by the nomogram. The estimated progress-free was plotted on *x*-axis; the observed progress-free rate was plotted on *y*-axis. **(D)** Decision curve analysis **(DCA)** of R-, C-, and Combi-models indicated their clinical net benefits. The blue, red, and yellow lines represented R-, C-, and Combi-models, respectively. DCA showed that the Combi-model gained more clinical net benefit than the other two models.

The Combi-model and nomogram (**Figure 5A**) were established based on Rad-score and the clinically relevant factors (cM stage, lung nodule type and histological type). A multivariate Cox regression model was used to evaluate the significance of Rad-score and these clinical factors in predicting PFS. Forest plots (**Figure 4B**) of the Combi-model illustrated that Rad-score (HR=0.034, p=0.001), cM stage (HR=3.822, p=0.002) and histological type (HR=0.191, p=0.002) were independent factors for PFS. Schoenfeld individual tests showed that the Combi-model fitted the proportional hazard assumption requirement (**Supplementary Figure 10**). The calibration curve showed that the calibration effect of the training and validation cohorts was very good (**Supplementary Figure 11**). The C-indexes of the Combi-model for predicting PFS in the training and external test cohorts were 0.845 and 0.837, respectively.

Finally, a nomogram combining Rad-score and clinical factors (cM stage, lung nodule type and histological type) was established (**Figure 5A**). The calibration curves showed good consistency between the predicted PFS probability and the actual result at one and two years after surgery (**Figures 5B, C**).

### Comparison among the R-, C-, and Combi-models

The pairwise comparison of C-indexes among the R-, C-, and Combi-model is shown in **Supplementary Table 4**. The Combi-model performed better than the R- and C-models. In the training cohort, the C-index of the Combi-model was 0.845, higher than the R- (0.781) and C-models (0.755). In the external test cohort, the C-index for the Combi-model was 0.837, significantly higher than the R- (0.778) and C-models (0.739) (all p<0.01). DCA was used to compare and visualize the clinical net benefits of the three models (**Figure 5D**) and showed that the Combi-model gained more clinical net benefits than the other two models.

### Solid Tumor *vs.* Subsolid Tumor

In the solid and subsolid tumor groups, Kaplan-Meier survival analysis showed that the Rad-score significantly discriminated the patients into high and low risk of progression (p=0.0045 and p<0.001, respectively) (**Supplementary Figure 12**).

## Discussion

In this study, we established machine-learning-derived radiomics models to predict PFS in patients with lung adenocarcinoma. The C-index of the radiomics model (Rad-score) reached 0.781 and 0.860 in the training and external test cohorts, respectively, higher than the TNM staging C-indexes of 0.707 and 0.606. The established nomogram combined Rad-score, cM stage, lung nodule type and histological subtype, and achieved a high C-index of 0.845 and 0.837 in the training and external test cohorts, respectively. According to several previous reports, the C-index of radiomic models for predicting post-surgical prognosis of lung cancer was between 0.60 and 0.67, and increased to 0.72 when combined with clinical and genomic characteristics (12, 23, 24).

The predictive value of radiomics for the prognosis of lung cancer is controversial. Some studies showed that radiomics had great potential in evaluating the prognosis of lung cancer (25, 26). Choe et al. reported that the radiomic features of contrast-enhanced CT had a similar ability to assess prognosis in a clinicopathological model of patients with adenocarcinoma after pneumonectomy (27). Wang et al. showed that radiomic features could successfully stratify patients into low- and high-risk groups of post-surgical recurrence (28). In contrast, Li et al. showed no significant correlation between radiomic features and PFS (29), and no additional benefit for adding clinical factors to the prediction model. Botta et al. found that the combined radiomic and clinical factor model did not improve the performance when compared with a stand-alone radiomic or clinical factor model (30). Although researchers have extracted many radiomic features in oncology imaging (27, 29, 31), the clinical relevance of radiomic features to predict lung cancer survival remains a major question. This study confirmed the predictive value and achieved a high C-index of 0.860 in the external test cohort.

One strength of this study is the availability of an external test cohort. An independent and external test cohort contributes to evaluate the generalizability of predictive models. Some methodological issues have been raised in prior studies on radiomics, including the heterogeneity in quantitative data dependent on different acquisition protocols and reconstruction parameters (32). It has been widely accepted that a comprehensive evaluation is necessary for a clinical biomarker, i.e., internal and external validation (33). Many researchers, such as Choe et al., believed that their risk prediction model needed further external verification to confirm its value for survival stratification (27). Another strength is the 3D lesion segmentation with 1,672 radiomic features. Most previous radiomics-based studies only performed 2D lesion segmentation. In our study, 3D lesion segmentation was the basis for numerous high-dimensional radiomic features, that maximize the potential information underlying the images and thus improved model performance.

The 2-year PFS used in this study is a relevant and widely-applied survival threshold for patients with lung adenocarcinoma (34, 35). Hosny et al. evaluated the application of deep learning in predicting 2-year overall survival rate of NSCLC patients based on CT data (36), and suggested the prognosis-predicting advantage for post-surgical patients. There were other studies investigating 2- to 3-year survival rate of lung cancer. For example, Wang et al. retrospectively analyzed the clinical data of 173 patients with NSCLC (37), and predicted the prognosis and survival time range of patients with clinically significant 3-year survival period as the prediction standard. Khorrami et al. studied 350 patients with NSCLC with an average follow-up time of 35.5 months (range: 1.6-107.5 months) (38). They reported the median time to recurrence was 17.5 months (range: 1.3-75.3 months). Thus, the investigation of 2-year PFS is clinically suitable for determining tumor recurrence, or progression-free survival for resected lung cancer.

The prediction of patient survival based on CT images is commonly used in clinical trials and medical practice. A well-established and -validated prognostic model based on radiomics can make the prediction more objective and accurate than traditional qualitative and quantitative methods (12). Some studies have shown that the radiomics method was more predictive than conventional clinical measurements to noninvasively describe tumor phenotypes (11, 39). Thus, the clinical usage of imaging biomarkers may benefit the clinical practice because they are noninvasive, reproducible, low-cost, and do not require human input (40). At present, there is no consensus on the strategy for post-surgical follow-up interval or follow-up examination method. For example, the National Comprehensive Cancer Network guideline recommends that for patients with stage I-II NSCLC, a medical history collection, chest CT and physical examination should be conducted every 6 months for 2 to 3 years after surgery, and then once a year for history collection, chest CT and physical examination (41). However, the phase III randomized controlled clinical trial (IFCT-0302) was different (42). In the first two years, the patients were followed up every six months, then yearly until the fifth year. In each follow-up examination, patients underwent clinical examination, chest and abdominal CT scanning and bronchoscopy examination. Watanabe et al. studied the post-surgical recurrence pattern of NSCLC (18), and found that the peak of risk curve was at 6-8 months after surgery. The next significant peak occurred at the end of the second year. For women, the peak occurred at 22-24 months after surgery, which was about 16 months later than that for men. Their results suggested that post-surgical recurrence time of lung cancer is variable. Therefore, developing an individualized follow-up strategy for the patients with lung adenocarcinoma is essential in clinical practice. A more frequent follow-up schedule is necessary for patients with high post-surgical progression risk.

We investigated the prognosis of solid and subsolid tumors (pure ground glass or part-solid attenuation), and found that subsolid tumors were associated with high PFS. Wang et al. reported that the overall 5-year survival of patients with stage I lung cancer was 83%, most of which were subsolid tumors (28). Although the image features differ between solid and subsolid tumors, in this study, regardless of tumor density type, the radiomics model demonstrated its ability to discriminate patients into high and low progression risk group (p<0.01). One reason for the model’s success is that the model selected the most relevant image features to predict PFS for both kinds of tumors, thus improving its generalizability.

We did not observe a correlation between *EGFR* mutations and PFS, so the genetic factor was not included in the Combi-model. Song et al. used radiomics to predict PFS in patients with stage IV *EGFR* mutant NSCLC, who received tyrosine kinase inhibitors therapy (7). The predictive value of *EGFR* mutation for survival prediction remains controversial (43). Some authors have found that *EGFR* mutation is a significant predictor of overall survival and relapse-free survival in surgically resected adenocarcinoma (44, 45), while others showed that *EGFR* mutation was insignificant for post-surgical survival (46). The differences between these studies may be due to the heterogeneous nature of lung cancer and the differences between study populations.

There were several limitations to this study. First, although the clinical data were from two large medical centers, more external test cohorts from different hospitals and regions are necessary to improve the robustness of these models. Second, the sample size of this study is not large. Although an external test helps to enhance the generalizability of the conclusions of this study, increasing the sample size would strengthen the robustness of models. Third, we only investigated patients with adenocarcinoma. Other types of lung cancer, such as squamous cell carcinoma, would show different tumor behaviors, which needs further investigation. Fourth, the study samples of this study were collected from the real-world clinical practice, in which a variety of postoperative radiotherapy, chemotherapy and targeted therapy were used. In order to evaluate the value of radiomics in predicting the prognosis of different treatment regimens, it is necessary to conduct well-controlled studies on specific treatment methods.

## Conclusion

The machine-learning-derived radiomics model surpassed only TNM stage in predicting post-surgical PFS in patients with lung adenocarcinoma. The established nomogram comprising CT-derived radiomic features and clinical risk factors demonstrated high performance in predicting post-surgical PFS, which was verified in the external test cohort. Our study shows that the high-dimensional information underlying CT images can be used to evaluate the post-surgical prognosis of lung adenocarcinoma, which may help clinicians to identify high-risk patients, make individualized treatment decisions, plan appropriate adjuvant therapeutic strategies, and personalize the follow-up scheme.

## Data Availability Statement

The raw data supporting the conclusions of this article will be made available by the authors, without undue reservation.

## Ethics Statement

The studies involving human participants were reviewed and approved by IRB of Shanghai General Hospital. The ethics committee waived the requirement of written informed consent for participation. Written informed consent was not obtained from the individual(s) for the publication of any potentially identifiable images or data included in this article.

## Author Contributions

Conception and design: XX. Administrative support: XX. Provision of study materials or patients: GL, ZX, and XX. Collection and assembly of data: GL, ZX, and XX. Data analysis and interpretation: GL, ZX, and XX. All authors contributed to the article and approved the submitted version.

## Funding

This study was sponsored by Ministry of Science and Technology of China (2016YFE0103000), National Natural Science Foundation of China (project no. 81971612 and 82001809), Shanghai Municipal Education Commission – Gaofeng Clinical Medicine Grant Support (20181814), and Clinical Research Innovation Plan of Shanghai General Hospital (CTCCR-2018B04, CTCCR-2019D05).

## Conflict of Interest

Author ZX was employed by company Siemens Healthineers Ltd.

The remaining authors declare that the research was conducted in the absence of any commercial or financial relationships that could be construed as a potential conflict of interest.
