# Supplementary material for: Machine-Learning-Derived Nomogram Based on 3D Radiomic Features and Clinical Factors Predicts Progression-Free Survival in Lung Adenocarcinoma
Source: Front Oncol. 2021 Jun 23;11:692329. doi: 10.3389/fonc.2021.692329 (PMC8260977; doi:10.3389/fonc.2021.692329)
Supplement: Supplementary file 1 [file DataSheet_1.docx]

**Supplementary materials**

**Supplementary Table 1.** CT acquisition protocols and image reconstruction parameters

|  | **Hospital-1** | | | **Hospital-2** | |
| --- | --- | --- | --- | --- | --- |
| **CT equipment** | **Siemens Somatom Force** | **GE Revolution** | **GE HD750** | **Siemens Somatom Flash** | **GE Revolution** |
| Acquisition mode | Helical | Helical | Helical | Helical | Helical |
| Tube voltage, kV | 100 | 120 | 120 | 100 | 120 |
| Tube current, mA | 130 (quality reference) | 100-400 (smart mode) | 100-400 (smart mode) | 130 (quality reference) | 100-400 (smart mode) |
| Collimation, mm | 192×0.6 | 256×0.625 | 64×0.625 | 192×0.6 | 256×0.625 |
| Pitch | 1.2 | 0.984 | 1.39 | 1.2 | 0.984 |
| Rotation time, ms | 500 | 500 | 500 | 500 | 500 |
| Reconstruction kernel | Br44 | Standard | Standard | Br44 | Standard |
| Field of view, mm | 325 | 325 | 325 | 325 | 325 |
| Slice thickness, mm | 0.6 | 0.625 | 0.625 | 0.6 | 0.625 |
| Slice increment, mm | 0.6 | 0.625 | 0.625 | 0.6 | 0.625 |
| Radiation dose, mGy | ≈4.52 | ≈5.20 | ≈10.78 | ≈4.52 | ≈5.20 |

**Supplementary Table 2.** The 597 stable radiomic features out of 1,672 selected by stability analysis

| **Radiomic feature** | **Spearman’s *r*** |
| --- | --- |
| original_shape_MajorAxis | 0.976086435 |
| log.sigma.0.5.mm.3D_glszm_SizeZoneNonUniformity | 0.973205282 |
| log.sigma.4.5.mm.3D_glszm_LargeAreaEmphasis | 0.972533013 |
| log.sigma.4.5.mm.3D_glszm_LargeAreaHighGrayLevelEmphasis | 0.969843938 |
| log.sigma.4.5.mm.3D_glcm_Id | 0.969459784 |
| log.sigma.0.5.mm.3D_glszm_GrayLevelNonUniformity | 0.969267707 |
| exponential_firstorder_Energy | 0.969171669 |
| exponential_firstorder_TotalEnergy | 0.969171669 |
| exponential_glrlm_GrayLevelNonUniformity | 0.96907563 |
| original_shape_SurfaceArea | 0.968307323 |
| log.sigma.4.5.mm.3D_glszm_ZoneVariance | 0.968307323 |
| original_gldm_DependenceNonUniformity | 0.968115246 |
| log.sigma.4.5.mm.3D_glcm_Idm | 0.968019208 |
| original_shape_Maximum3DDiameter | 0.966937982 |
| log.sigma.3.5.mm.3D_glszm_LargeAreaHighGrayLevelEmphasis | 0.966290516 |
| wavelet.LLH_glszm_GrayLevelNonUniformity | 0.965906363 |
| squareroot_firstorder_Energy | 0.965522209 |
| squareroot_firstorder_TotalEnergy | 0.965522209 |
| log.sigma.3.5.mm.3D_gldm_GrayLevelNonUniformity | 0.96542617 |
| square_glrlm_GrayLevelNonUniformity | 0.965330132 |
| logarithm_glszm_GrayLevelNonUniformity | 0.965042017 |
| log.sigma.4.5.mm.3D_gldm_GrayLevelNonUniformity | 0.964465786 |
| log.sigma.0.5.mm.3D_gldm_DependenceNonUniformity | 0.964273709 |
| log.sigma.4.5.mm.3D_glrlm_RunPercentage | 0.963409364 |
| log.sigma.4.5.mm.3D_glrlm_GrayLevelNonUniformity | 0.963313325 |
| log.sigma.4.5.mm.3D_ngtdm_Contrast | 0.962927393 |
| log.sigma.4.5.mm.3D_gldm_LargeDependenceEmphasis | 0.962545018 |
| original_firstorder_90Percentile | 0.962092832 |
| log.sigma.3.5.mm.3D_glrlm_RunVariance | 0.961872749 |
| log.sigma.4.5.mm.3D_ngtdm_Complexity | 0.961680672 |
| logarithm_glszm_LargeAreaLowGrayLevelEmphasis | 0.961392557 |
| wavelet.LHH_gldm_DependenceNonUniformity | 0.96120048 |
| log.sigma.4.5.mm.3D_glrlm_RunLengthNonUniformityNormalized | 0.960816327 |
| wavelet.LHL_glszm_GrayLevelNonUniformity | 0.960336134 |
| log.sigma.4.5.mm.3D_glrlm_RunVariance | 0.959471789 |
| log.sigma.4.5.mm.3D_glrlm_ShortRunEmphasis | 0.959471789 |
| log.sigma.4.5.mm.3D_glrlm_LongRunEmphasis | 0.959279712 |
| log.sigma.3.5.mm.3D_glrlm_GrayLevelNonUniformity | 0.959087635 |
| wavelet.LLL_glrlm_RunLengthNonUniformity | 0.958991597 |
| log.sigma.3.5.mm.3D_glcm_Id | 0.958895558 |
| logarithm_firstorder_Energy | 0.95879952 |
| logarithm_firstorder_TotalEnergy | 0.95879952 |
| log.sigma.3.5.mm.3D_gldm_LargeDependenceHighGrayLevelEmphasis | 0.958703481 |
| log.sigma.1.5.mm.3D_firstorder_RootMeanSquared | 0.958607443 |
| log.sigma.3.5.mm.3D_ngtdm_Contrast | 0.958031212 |
| wavelet.LHH_glszm_GrayLevelNonUniformity | 0.957935174 |
| log.sigma.3.5.mm.3D_glcm_Idm | 0.957647059 |
| logarithm_glszm_LargeAreaHighGrayLevelEmphasis | 0.957454982 |
| wavelet.HLH_glrlm_RunLengthNonUniformity | 0.957358944 |
| log.sigma.3.5.mm.3D_glrlm_LongRunEmphasis | 0.957166867 |
| log.sigma.4.5.mm.3D_glcm_DifferenceAverage | 0.957070828 |
| original_shape_Maximum2DDiameterRow | 0.957067736 |
| log.sigma.3.5.mm.3D_glrlm_RunPercentage | 0.956302521 |
| log.sigma.4.5.mm.3D_ngtdm_Coarseness | 0.956014406 |
| squareroot_glszm_GrayLevelNonUniformity | 0.955822329 |
| wavelet.LLH_glrlm_GrayLevelNonUniformity | 0.955630252 |
| original_glrlm_GrayLevelNonUniformity | 0.955534214 |
| log.sigma.4.5.mm.3D_glszm_LargeAreaLowGrayLevelEmphasis | 0.955534214 |
| square_gldm_GrayLevelNonUniformity | 0.955246098 |
| log.sigma.1.5.mm.3D_ngtdm_Contrast | 0.954884871 |
| original_glszm_ZoneVariance | 0.954861945 |
| logarithm_glrlm_RunLengthNonUniformity | 0.954669868 |
| log.sigma.2.5.mm.3D_ngtdm_Contrast | 0.95461967 |
| wavelet.LHL_glrlm_RunLengthNonUniformity | 0.954477791 |
| original_glszm_LargeAreaEmphasis | 0.954381753 |
| original_ngtdm_Coarseness | 0.954381753 |
| log.sigma.3.5.mm.3D_ngtdm_Complexity | 0.954381753 |
| wavelet.LLH_ngtdm_Coarseness | 0.954093637 |
| log.sigma.0.5.mm.3D_gldm_GrayLevelNonUniformity | 0.953613445 |
| wavelet.LLL_glszm_GrayLevelNonUniformity | 0.952845138 |
| log.sigma.3.5.mm.3D_glszm_ZoneVariance | 0.952845138 |
| log.sigma.3.5.mm.3D_glcm_DifferenceAverage | 0.9527491 |
| log.sigma.2.5.mm.3D_glszm_LargeAreaHighGrayLevelEmphasis | 0.952460984 |
| log.sigma.3.5.mm.3D_glszm_LargeAreaEmphasis | 0.952172869 |
| wavelet.HLL_glrlm_RunLengthNonUniformity | 0.951788715 |
| original_shape_Volume | 0.951692677 |
| exponential_gldm_GrayLevelNonUniformity | 0.951692677 |
| wavelet.HHH_glrlm_RunLengthNonUniformity | 0.951692677 |
| original_gldm_GrayLevelNonUniformity | 0.951116447 |
| squareroot_glszm_LargeAreaHighGrayLevelEmphasis | 0.951116447 |
| log.sigma.4.5.mm.3D_ngtdm_Busyness | 0.951116447 |
| wavelet.LHH_glrlm_RunLengthNonUniformity | 0.951020408 |
| wavelet.HHL_glrlm_RunLengthNonUniformity | 0.95092437 |
| log.sigma.0.5.mm.3D_ngtdm_Coarseness | 0.95092437 |
| log.sigma.3.5.mm.3D_glrlm_RunLengthNonUniformityNormalized | 0.95092437 |
| log.sigma.3.5.mm.3D_ngtdm_Coarseness | 0.95092437 |
| log.sigma.4.5.mm.3D_gldm_DependenceNonUniformityNormalized | 0.950732293 |
| squareroot_gldm_DependenceNonUniformity | 0.950540216 |
| log.sigma.3.5.mm.3D_gldm_SmallDependenceEmphasis | 0.950540216 |
| log.sigma.0.5.mm.3D_glrlm_GrayLevelNonUniformity | 0.950348139 |
| log.sigma.1.5.mm.3D_gldm_DependenceNonUniformity | 0.950348139 |
| log.sigma.2.5.mm.3D_ngtdm_Complexity | 0.950156062 |
| logarithm_glrlm_GrayLevelNonUniformity | 0.950060024 |
| log.sigma.2.5.mm.3D_gldm_GrayLevelNonUniformity | 0.949963986 |
| wavelet.HLH_gldm_DependenceNonUniformity | 0.949483794 |
| log.sigma.0.5.mm.3D_glrlm_RunLengthNonUniformity | 0.948907563 |
| log.sigma.0.5.mm.3D_ngtdm_Contrast | 0.948665003 |
| wavelet.LLH_gldm_DependenceNonUniformity | 0.948523409 |
| log.sigma.1.5.mm.3D_gldm_GrayLevelNonUniformity | 0.948235294 |
| squareroot_glrlm_RunLengthNonUniformity | 0.948139256 |
| log.sigma.1.5.mm.3D_glszm_LargeAreaHighGrayLevelEmphasis | 0.948043217 |
| log.sigma.3.5.mm.3D_glrlm_ShortRunEmphasis | 0.947947179 |
| log.sigma.2.5.mm.3D_glcm_Idm | 0.94785114 |
| wavelet.LLH_gldm_GrayLevelNonUniformity | 0.947466987 |
| log.sigma.2.5.mm.3D_gldm_DependenceNonUniformity | 0.947466987 |
| log.sigma.3.5.mm.3D_gldm_LargeDependenceEmphasis | 0.947466987 |
| log.sigma.2.5.mm.3D_glrlm_GrayLevelNonUniformity | 0.947370948 |
| squareroot_firstorder_90Percentile | 0.947178872 |
| log.sigma.1.5.mm.3D_firstorder_Median | 0.947178872 |
| log.sigma.2.5.mm.3D_glcm_Id | 0.946986795 |
| log.sigma.1.5.mm.3D_firstorder_10Percentile | 0.946890756 |
| log.sigma.4.5.mm.3D_ngtdm_Strength | 0.946890756 |
| log.sigma.1.5.mm.3D_glcm_MaximumProbability | 0.946698679 |
| wavelet.LHL_ngtdm_Coarseness | 0.946314526 |
| wavelet.LHL_gldm_DependenceNonUniformity | 0.946218487 |
| original_shape_Maximum2DDiameterSlice | 0.946088089 |
| log.sigma.3.5.mm.3D_glszm_ZonePercentage | 0.945834334 |
| squareroot_glrlm_GrayLevelNonUniformity | 0.945738295 |
| log.sigma.1.5.mm.3D_glrlm_GrayLevelNonUniformity | 0.945354142 |
| wavelet.HHH_gldm_DependenceNonUniformity | 0.945162065 |
| original_ngtdm_Contrast | 0.944944657 |
| wavelet.LHH_ngtdm_Coarseness | 0.94487395 |
| log.sigma.4.5.mm.3D_glcm_Contrast | 0.944585834 |
| original_firstorder_Energy | 0.944489796 |
| original_firstorder_TotalEnergy | 0.944489796 |
| log.sigma.1.5.mm.3D_firstorder_Mean | 0.944105642 |
| log.sigma.2.5.mm.3D_gldm_DependenceNonUniformityNormalized | 0.944105642 |
| log.sigma.2.5.mm.3D_glcm_DifferenceAverage | 0.944009604 |
| log.sigma.1.5.mm.3D_glrlm_RunLengthNonUniformity | 0.94362545 |
| log.sigma.3.5.mm.3D_gldm_DependenceNonUniformityNormalized | 0.94362545 |
| original_glrlm_RunLengthNonUniformity | 0.943337335 |
| logarithm_gldm_GrayLevelNonUniformity | 0.942953181 |
| log.sigma.0.5.mm.3D_glszm_LargeAreaEmphasis | 0.942857143 |
| log.sigma.4.5.mm.3D_gldm_DependenceVariance | 0.942761104 |
| log.sigma.1.5.mm.3D_ngtdm_Coarseness | 0.942665066 |
| log.sigma.2.5.mm.3D_glrlm_ShortRunEmphasis | 0.942569028 |
| log.sigma.0.5.mm.3D_glszm_ZoneVariance | 0.942376951 |
| log.sigma.1.5.mm.3D_glrlm_LongRunEmphasis | 0.942376951 |
| original_glszm_GrayLevelNonUniformity | 0.941896759 |
| wavelet.LLH_glrlm_RunLengthNonUniformity | 0.94180072 |
| log.sigma.2.5.mm.3D_glrlm_RunLengthNonUniformity | 0.94180072 |
| wavelet.LLH_glszm_LargeAreaLowGrayLevelEmphasis | 0.941704682 |
| log.sigma.2.5.mm.3D_glrlm_RunPercentage | 0.941608643 |
| log.sigma.4.5.mm.3D_gldm_SmallDependenceEmphasis | 0.941608643 |
| log.sigma.4.5.mm.3D_glszm_ZonePercentage | 0.941608643 |
| wavelet.LHL_glszm_SizeZoneNonUniformity | 0.941416567 |
| log.sigma.2.5.mm.3D_gldm_LargeDependenceHighGrayLevelEmphasis | 0.941416567 |
| logarithm_gldm_DependenceNonUniformity | 0.941128451 |
| log.sigma.0.5.mm.3D_firstorder_Mean | 0.941128451 |
| squareroot_gldm_GrayLevelNonUniformity | 0.941032413 |
| wavelet.HHL_glszm_GrayLevelNonUniformity | 0.941032413 |
| log.sigma.2.5.mm.3D_firstorder_Skewness | 0.940936375 |
| wavelet.HHH_glszm_GrayLevelNonUniformity | 0.940744298 |
| log.sigma.1.5.mm.3D_gldm_DependenceNonUniformityNormalized | 0.940744298 |
| log.sigma.1.5.mm.3D_glrlm_RunVariance | 0.940744298 |
| log.sigma.1.5.mm.3D_glszm_ZoneVariance | 0.940168067 |
| wavelet.LLL_gldm_DependenceNonUniformity | 0.940072029 |
| squareroot_ngtdm_Coarseness | 0.939591837 |
| wavelet.HLH_glszm_GrayLevelNonUniformity | 0.938919568 |
| square_glrlm_RunVariance | 0.938631453 |
| log.sigma.0.5.mm.3D_glszm_LargeAreaHighGrayLevelEmphasis | 0.938631453 |
| log.sigma.2.5.mm.3D_glrlm_RunLengthNonUniformityNormalized | 0.938631453 |
| log.sigma.2.5.mm.3D_ngtdm_Coarseness | 0.938343337 |
| log.sigma.1.5.mm.3D_glszm_LargeAreaEmphasis | 0.938151261 |
| log.sigma.3.5.mm.3D_glcm_Contrast | 0.937767107 |
| log.sigma.0.5.mm.3D_ngtdm_Busyness | 0.937094838 |
| log.sigma.2.5.mm.3D_glrlm_LongRunEmphasis | 0.936710684 |
| log.sigma.1.5.mm.3D_glcm_Idn | 0.936422569 |
| original_shape_MinorAxis | 0.936038415 |
| log.sigma.3.5.mm.3D_glcm_DifferenceEntropy | 0.93517407 |
| wavelet.LLH_firstorder_Median | 0.935078031 |
| squareroot_ngtdm_Strength | 0.934597839 |
| log.sigma.1.5.mm.3D_gldm_LargeDependenceEmphasis | 0.934597839 |
| log.sigma.2.5.mm.3D_glrlm_RunVariance | 0.934501801 |
| logarithm_firstorder_Median | 0.934213685 |
| log.sigma.0.5.mm.3D_firstorder_Median | 0.934213685 |
| log.sigma.2.5.mm.3D_glszm_ZoneVariance | 0.934021609 |
| log.sigma.2.5.mm.3D_glcm_Imc1 | 0.93392557 |
| wavelet.HLH_ngtdm_Coarseness | 0.933445378 |
| log.sigma.1.5.mm.3D_glcm_Idm | 0.933157263 |
| log.sigma.2.5.mm.3D_gldm_LargeDependenceEmphasis | 0.932773109 |
| log.sigma.1.5.mm.3D_glcm_Id | 0.932677071 |
| original_shape_Maximum2DDiameterColumn | 0.93245612 |
| log.sigma.1.5.mm.3D_glrlm_RunPercentage | 0.932388956 |
| log.sigma.2.5.mm.3D_glszm_LargeAreaEmphasis | 0.931908764 |
| log.sigma.3.5.mm.3D_glrlm_RunLengthNonUniformity | 0.93152461 |
| log.sigma.3.5.mm.3D_ngtdm_Strength | 0.930372149 |
| log.sigma.1.5.mm.3D_glrlm_ShortRunEmphasis | 0.930180072 |
| log.sigma.1.5.mm.3D_glszm_GrayLevelNonUniformity | 0.929987995 |
| log.sigma.2.5.mm.3D_glcm_ClusterShade | 0.929411765 |
| log.sigma.2.5.mm.3D_glcm_MaximumProbability | 0.929411765 |
| logarithm_ngtdm_Complexity | 0.929219688 |
| wavelet.LLL_ngtdm_Coarseness | 0.928739496 |
| log.sigma.1.5.mm.3D_glcm_JointEnergy | 0.928643457 |
| log.sigma.2.5.mm.3D_ngtdm_Strength | 0.928643457 |
| squareroot_glszm_LargeAreaEmphasis | 0.928163265 |
| exponential_glrlm_RunLengthNonUniformity | 0.928163265 |
| wavelet.LLL_glszm_SizeZoneNonUniformity | 0.928067227 |
| squareroot_glszm_ZoneVariance | 0.927779112 |
| log.sigma.4.5.mm.3D_glrlm_RunLengthNonUniformity | 0.927587035 |
| log.sigma.0.5.mm.3D_glszm_LargeAreaLowGrayLevelEmphasis | 0.927490996 |
| log.sigma.1.5.mm.3D_gldm_DependenceVariance | 0.926914766 |
| original_shape_LeastAxis | 0.926818727 |
| log.sigma.1.5.mm.3D_glrlm_RunLengthNonUniformityNormalized | 0.926818727 |
| log.sigma.2.5.mm.3D_glcm_Contrast | 0.92547419 |
| log.sigma.3.5.mm.3D_glcm_DifferenceVariance | 0.925378151 |
| wavelet.HLH_glszm_SizeZoneNonUniformity | 0.925090036 |
| log.sigma.1.5.mm.3D_glcm_DifferenceAverage | 0.924609844 |
| wavelet.LLH_ngtdm_Contrast | 0.923789858 |
| wavelet.HHH_ngtdm_Coarseness | 0.92364946 |
| log.sigma.2.5.mm.3D_glrlm_LongRunHighGrayLevelEmphasis | 0.923169268 |
| exponential_glrlm_RunVariance | 0.922977191 |
| log.sigma.0.5.mm.3D_ngtdm_Complexity | 0.922881152 |
| exponential_gldm_DependenceNonUniformity | 0.922689076 |
| log.sigma.2.5.mm.3D_firstorder_Energy | 0.922689076 |
| log.sigma.2.5.mm.3D_firstorder_TotalEnergy | 0.922689076 |
| log.sigma.2.5.mm.3D_glcm_Imc2 | 0.92240096 |
| original_glszm_LargeAreaHighGrayLevelEmphasis | 0.922208884 |
| squareroot_ngtdm_Complexity | 0.922208884 |
| squareroot_ngtdm_Contrast | 0.921990924 |
| log.sigma.0.5.mm.3D_firstorder_Uniformity | 0.921728691 |
| square_gldm_DependenceNonUniformity | 0.921056423 |
| wavelet.LLH_glszm_LargeAreaEmphasis | 0.920192077 |
| log.sigma.4.5.mm.3D_glcm_Idn | 0.920192077 |
| wavelet.LHL_glrlm_GrayLevelNonUniformity | 0.919903962 |
| wavelet.LLH_glszm_ZoneVariance | 0.919519808 |
| log.sigma.1.5.mm.3D_gldm_LargeDependenceHighGrayLevelEmphasis | 0.91942377 |
| log.sigma.3.5.mm.3D_gldm_DependenceVariance | 0.918847539 |
| log.sigma.2.5.mm.3D_glcm_DifferenceEntropy | 0.918751501 |
| log.sigma.0.5.mm.3D_firstorder_RootMeanSquared | 0.918655462 |
| wavelet.HHL_gldm_DependenceNonUniformity | 0.918079232 |
| log.sigma.2.5.mm.3D_glcm_Correlation | 0.917791116 |
| log.sigma.0.5.mm.3D_firstorder_10Percentile | 0.917695078 |
| log.sigma.1.5.mm.3D_glszm_LargeAreaLowGrayLevelEmphasis | 0.917118848 |
| log.sigma.2.5.mm.3D_firstorder_10Percentile | 0.916734694 |
| squareroot_ngtdm_Busyness | 0.915966387 |
| log.sigma.2.5.mm.3D_glrlm_RunEntropy | 0.915678271 |
| log.sigma.3.5.mm.3D_gldm_DependenceNonUniformity | 0.915582233 |
| log.sigma.4.5.mm.3D_gldm_DependenceNonUniformity | 0.915198079 |
| log.sigma.2.5.mm.3D_gldm_SmallDependenceEmphasis | 0.914813926 |
| wavelet.LLL_glrlm_GrayLevelNonUniformity | 0.91452581 |
| wavelet.LLL_ngtdm_Complexity | 0.91452581 |
| log.sigma.0.5.mm.3D_glcm_InverseVariance | 0.914429772 |
| square_glszm_ZoneVariance | 0.914333733 |
| square_glrlm_LongRunLowGrayLevelEmphasis | 0.913181273 |
| square_glrlm_LongRunEmphasis | 0.913085234 |
| wavelet.HHL_ngtdm_Coarseness | 0.912797119 |
| log.sigma.1.5.mm.3D_glcm_DifferenceEntropy | 0.912605042 |
| wavelet.LHL_ngtdm_Contrast | 0.912220888 |
| square_glszm_LargeAreaEmphasis | 0.911548619 |
| square_glszm_LargeAreaLowGrayLevelEmphasis | 0.911548619 |
| wavelet.LLH_glszm_SizeZoneNonUniformity | 0.911260504 |
| log.sigma.1.5.mm.3D_glcm_Idmn | 0.911068427 |
| log.sigma.2.5.mm.3D_ngtdm_Busyness | 0.910972389 |
| wavelet.LLL_ngtdm_Contrast | 0.910108043 |
| original_glszm_SizeZoneNonUniformity | 0.910012005 |
| squareroot_glszm_SizeZoneNonUniformity | 0.909435774 |
| logarithm_firstorder_90Percentile | 0.909147659 |
| log.sigma.2.5.mm.3D_glszm_SizeZoneNonUniformity | 0.909147659 |
| logarithm_glszm_LargeAreaEmphasis | 0.908955582 |
| log.sigma.3.5.mm.3D_glcm_Idn | 0.908955582 |
| logarithm_glszm_ZoneVariance | 0.908859544 |
| log.sigma.1.5.mm.3D_glcm_JointEntropy | 0.908283313 |
| log.sigma.2.5.mm.3D_glcm_JointEnergy | 0.908187275 |
| log.sigma.3.5.mm.3D_glrlm_LongRunHighGrayLevelEmphasis | 0.908091236 |
| log.sigma.3.5.mm.3D_gldm_SmallDependenceHighGrayLevelEmphasis | 0.907611044 |
| square_glszm_LargeAreaHighGrayLevelEmphasis | 0.907418968 |
| wavelet.LHL_gldm_GrayLevelNonUniformity | 0.907130852 |
| log.sigma.1.5.mm.3D_ngtdm_Complexity | 0.907130852 |
| log.sigma.2.5.mm.3D_glszm_ZonePercentage | 0.907130852 |
| log.sigma.2.5.mm.3D_glszm_LargeAreaLowGrayLevelEmphasis | 0.907034814 |
| logarithm_ngtdm_Busyness | 0.906842737 |
| log.sigma.0.5.mm.3D_glcm_Idm | 0.90665066 |
| log.sigma.0.5.mm.3D_firstorder_InterquartileRange | 0.906362545 |
| log.sigma.0.5.mm.3D_glcm_JointEnergy | 0.906266507 |
| log.sigma.2.5.mm.3D_gldm_DependenceVariance | 0.905786315 |
| log.sigma.0.5.mm.3D_glcm_DifferenceAverage | 0.905690276 |
| wavelet.HLL_gldm_DependenceNonUniformity | 0.905594238 |
| log.sigma.1.5.mm.3D_glcm_Contrast | 0.905498199 |
| log.sigma.3.5.mm.3D_firstorder_Energy | 0.905402161 |
| log.sigma.3.5.mm.3D_firstorder_TotalEnergy | 0.905402161 |
| log.sigma.3.5.mm.3D_glszm_GrayLevelNonUniformity | 0.90482593 |
| original_ngtdm_Complexity | 0.904729892 |
| log.sigma.1.5.mm.3D_glrlm_LongRunHighGrayLevelEmphasis | 0.904441777 |
| log.sigma.3.5.mm.3D_glcm_MaximumProbability | 0.904441777 |
| log.sigma.3.5.mm.3D_gldm_SmallDependenceLowGrayLevelEmphasis | 0.904153661 |
| log.sigma.4.5.mm.3D_glszm_GrayLevelNonUniformity | 0.904057623 |
| logarithm_glszm_SizeZoneNonUniformity | 0.903865546 |
| log.sigma.0.5.mm.3D_ngtdm_Strength | 0.903865546 |
| log.sigma.3.5.mm.3D_glcm_JointEntropy | 0.903769508 |
| log.sigma.0.5.mm.3D_firstorder_RobustMeanAbsoluteDeviation | 0.903193277 |
| log.sigma.3.5.mm.3D_glszm_SizeZoneNonUniformity | 0.9030012 |
| log.sigma.2.5.mm.3D_glcm_Idn | 0.902617047 |
| log.sigma.1.5.mm.3D_firstorder_Energy | 0.902136855 |
| log.sigma.1.5.mm.3D_firstorder_TotalEnergy | 0.902136855 |
| log.sigma.4.5.mm.3D_gldm_SmallDependenceLowGrayLevelEmphasis | 0.902136855 |
| log.sigma.0.5.mm.3D_glcm_Id | 0.901848739 |
| logarithm_ngtdm_Coarseness | 0.901752701 |
| log.sigma.0.5.mm.3D_glrlm_LongRunEmphasis | 0.901752701 |
| log.sigma.4.5.mm.3D_glcm_MaximumProbability | 0.901368547 |
| exponential_glrlm_RunEntropy | 0.901176471 |
| log.sigma.0.5.mm.3D_glrlm_RunVariance | 0.901080432 |
| log.sigma.0.5.mm.3D_glcm_JointEntropy | 0.900696279 |
| log.sigma.1.5.mm.3D_gldm_SmallDependenceEmphasis | 0.899831933 |
| log.sigma.1.5.mm.3D_glcm_Imc1 | 0.899735894 |
| log.sigma.4.5.mm.3D_gldm_LargeDependenceHighGrayLevelEmphasis | 0.899159664 |
| wavelet.LLH_gldm_SmallDependenceEmphasis | 0.898871549 |
| wavelet.LLL_gldm_GrayLevelNonUniformity | 0.89877551 |
| original_shape_Flatness | 0.898487395 |
| log.sigma.0.5.mm.3D_firstorder_MeanAbsoluteDeviation | 0.898487395 |
| log.sigma.0.5.mm.3D_gldm_LargeDependenceEmphasis | 0.898295318 |
| log.sigma.0.5.mm.3D_firstorder_Entropy | 0.897911164 |
| log.sigma.2.5.mm.3D_firstorder_RootMeanSquared | 0.897334934 |
| log.sigma.3.5.mm.3D_glszm_LargeAreaLowGrayLevelEmphasis | 0.897238896 |
| log.sigma.2.5.mm.3D_glcm_JointEntropy | 0.896854742 |
| log.sigma.0.5.mm.3D_glrlm_GrayLevelNonUniformityNormalized | 0.896182473 |
| log.sigma.0.5.mm.3D_glcm_SumEntropy | 0.895798319 |
| log.sigma.0.5.mm.3D_glcm_DifferenceEntropy | 0.895702281 |
| log.sigma.4.5.mm.3D_firstorder_Energy | 0.895702281 |
| log.sigma.4.5.mm.3D_firstorder_TotalEnergy | 0.895702281 |
| log.sigma.4.5.mm.3D_glcm_DifferenceEntropy | 0.895702281 |
| log.sigma.0.5.mm.3D_glcm_Contrast | 0.895510204 |
| log.sigma.0.5.mm.3D_glrlm_RunPercentage | 0.895414166 |
| wavelet.LHH_glrlm_GrayLevelNonUniformity | 0.895222089 |
| wavelet.LLH_glszm_ZonePercentage | 0.894645858 |
| squareroot_firstorder_Median | 0.89454982 |
| log.sigma.2.5.mm.3D_gldm_SmallDependenceLowGrayLevelEmphasis | 0.89454982 |
| log.sigma.3.5.mm.3D_gldm_DependenceEntropy | 0.893493397 |
| log.sigma.1.5.mm.3D_glszm_SizeZoneNonUniformity | 0.893301321 |
| log.sigma.3.5.mm.3D_glcm_InverseVariance | 0.893013205 |
| logarithm_gldm_LargeDependenceHighGrayLevelEmphasis | 0.892917167 |
| log.sigma.0.5.mm.3D_glrlm_ShortRunEmphasis | 0.892533013 |
| log.sigma.4.5.mm.3D_glcm_JointEnergy | 0.891956783 |
| log.sigma.0.5.mm.3D_firstorder_Energy | 0.891860744 |
| log.sigma.0.5.mm.3D_firstorder_TotalEnergy | 0.891860744 |
| log.sigma.0.5.mm.3D_gldm_SmallDependenceEmphasis | 0.891764706 |
| log.sigma.4.5.mm.3D_glcm_Idmn | 0.891668667 |
| logarithm_ngtdm_Contrast | 0.891572629 |
| log.sigma.0.5.mm.3D_glrlm_RunLengthNonUniformityNormalized | 0.891092437 |
| log.sigma.2.5.mm.3D_glszm_GrayLevelNonUniformity | 0.891092437 |
| wavelet.LHH_glszm_SizeZoneNonUniformity | 0.890996399 |
| exponential_firstorder_90Percentile | 0.89090036 |
| log.sigma.1.5.mm.3D_glszm_ZonePercentage | 0.888979592 |
| square_firstorder_InterquartileRange | 0.888403361 |
| log.sigma.0.5.mm.3D_glcm_SumSquares | 0.888307323 |
| wavelet.HLH_glrlm_GrayLevelNonUniformity | 0.8872509 |
| squareroot_firstorder_RootMeanSquared | 0.887154862 |
| wavelet.HLL_glszm_GrayLevelNonUniformity | 0.886866747 |
| log.sigma.1.5.mm.3D_glrlm_RunEntropy | 0.886482593 |
| log.sigma.2.5.mm.3D_glcm_Idmn | 0.886482593 |
| original_glrlm_LongRunEmphasis | 0.886386555 |
| log.sigma.1.5.mm.3D_firstorder_Skewness | 0.886194478 |
| original_glrlm_RunVariance | 0.885330132 |
| log.sigma.0.5.mm.3D_glcm_DifferenceVariance | 0.884945978 |
| original_gldm_LargeDependenceEmphasis | 0.88484994 |
| log.sigma.4.5.mm.3D_gldm_SmallDependenceHighGrayLevelEmphasis | 0.884753902 |
| exponential_glrlm_LongRunEmphasis | 0.884465786 |
| exponential_glrlm_LongRunHighGrayLevelEmphasis | 0.884465786 |
| exponential_glrlm_LongRunLowGrayLevelEmphasis | 0.884465786 |
| logarithm_glcm_Imc1 | 0.884081633 |
| log.sigma.0.5.mm.3D_glcm_MaximumProbability | 0.883121248 |
| square_gldm_LargeDependenceLowGrayLevelEmphasis | 0.882545018 |
| log.sigma.3.5.mm.3D_glcm_Imc1 | 0.882256903 |
| log.sigma.1.5.mm.3D_gldm_SmallDependenceLowGrayLevelEmphasis | 0.881968788 |
| log.sigma.2.5.mm.3D_glcm_DifferenceVariance | 0.881968788 |
| square_glrlm_RunPercentage | 0.881776711 |
| logarithm_glrlm_LongRunHighGrayLevelEmphasis | 0.881776711 |
| square_gldm_LargeDependenceEmphasis | 0.881584634 |
| log.sigma.3.5.mm.3D_glcm_Idmn | 0.881488595 |
| log.sigma.0.5.mm.3D_glcm_Idn | 0.881296519 |
| log.sigma.3.5.mm.3D_firstorder_10Percentile | 0.880720288 |
| log.sigma.0.5.mm.3D_glszm_ZonePercentage | 0.880432173 |
| log.sigma.0.5.mm.3D_glcm_ClusterTendency | 0.880048019 |
| log.sigma.3.5.mm.3D_ngtdm_Busyness | 0.879087635 |
| wavelet.LHH_gldm_GrayLevelNonUniformity | 0.878703481 |
| original_gldm_DependenceVariance | 0.878607443 |
| wavelet.HHH_glszm_LargeAreaHighGrayLevelEmphasis | 0.878127251 |
| log.sigma.3.5.mm.3D_glcm_JointEnergy | 0.87755102 |
| wavelet.LLH_ngtdm_Complexity | 0.877358944 |
| original_glrlm_RunPercentage | 0.876398559 |
| log.sigma.0.5.mm.3D_glrlm_GrayLevelVariance | 0.876110444 |
| log.sigma.2.5.mm.3D_glcm_InverseVariance | 0.87515006 |
| log.sigma.0.5.mm.3D_firstorder_Variance | 0.874957983 |
| log.sigma.2.5.mm.3D_gldm_DependenceEntropy | 0.874861945 |
| square_glrlm_RunEntropy | 0.874093637 |
| original_glrlm_ShortRunEmphasis | 0.873805522 |
| wavelet.HLH_gldm_GrayLevelNonUniformity | 0.873613445 |
| log.sigma.0.5.mm.3D_gldm_GrayLevelVariance | 0.87332533 |
| square_firstorder_RobustMeanAbsoluteDeviation | 0.873229292 |
| exponential_glszm_LargeAreaEmphasis | 0.873133253 |
| exponential_glszm_LargeAreaHighGrayLevelEmphasis | 0.873133253 |
| exponential_glszm_LargeAreaLowGrayLevelEmphasis | 0.873133253 |
| square_gldm_DependenceEntropy | 0.872845138 |
| original_ngtdm_Strength | 0.8727491 |
| original_glrlm_RunLengthNonUniformityNormalized | 0.871884754 |
| square_firstorder_Median | 0.871596639 |
| original_glcm_Id | 0.8715006 |
| original_glcm_Idm | 0.871308523 |
| original_glszm_ZonePercentage | 0.871308523 |
| exponential_firstorder_RobustMeanAbsoluteDeviation | 0.871020408 |
| log.sigma.2.5.mm.3D_firstorder_Median | 0.870444178 |
| log.sigma.4.5.mm.3D_glcm_JointEntropy | 0.869291717 |
| wavelet.HLH_gldm_SmallDependenceLowGrayLevelEmphasis | 0.868715486 |
| log.sigma.0.5.mm.3D_glcm_Idmn | 0.868619448 |
| wavelet.LLH_firstorder_10Percentile | 0.868331333 |
| log.sigma.2.5.mm.3D_firstorder_Kurtosis | 0.868043217 |
| log.sigma.1.5.mm.3D_firstorder_Uniformity | 0.867755102 |
| logarithm_ngtdm_Strength | 0.867370948 |
| log.sigma.4.5.mm.3D_glszm_SizeZoneNonUniformity | 0.867082833 |
| original_glcm_DifferenceAverage | 0.866794718 |
| log.sigma.4.5.mm.3D_glcm_Imc1 | 0.866314526 |
| wavelet.HLL_ngtdm_Coarseness | 0.866218487 |
| original_gldm_SmallDependenceEmphasis | 0.866026411 |
| exponential_firstorder_InterquartileRange | 0.865930372 |
| log.sigma.4.5.mm.3D_gldm_DependenceEntropy | 0.865162065 |
| log.sigma.0.5.mm.3D_firstorder_Kurtosis | 0.865066026 |
| wavelet.LLL_gldm_SmallDependenceLowGrayLevelEmphasis | 0.864969988 |
| square_glrlm_RunLengthNonUniformityNormalized | 0.862761104 |
| wavelet.LHH_glszm_LargeAreaHighGrayLevelEmphasis | 0.862761104 |
| logarithm_gldm_HighGrayLevelEmphasis | 0.862088836 |
| square_gldm_LargeDependenceHighGrayLevelEmphasis | 0.86180072 |
| logarithm_gldm_SmallDependenceLowGrayLevelEmphasis | 0.86180072 |
| log.sigma.0.5.mm.3D_gldm_SmallDependenceLowGrayLevelEmphasis | 0.861608643 |
| log.sigma.3.5.mm.3D_firstorder_Variance | 0.861416567 |
| log.sigma.3.5.mm.3D_gldm_GrayLevelVariance | 0.861128451 |
| wavelet.HLL_gldm_SmallDependenceLowGrayLevelEmphasis | 0.860936375 |
| log.sigma.1.5.mm.3D_glcm_DifferenceVariance | 0.860648259 |
| logarithm_glcm_JointAverage | 0.860456182 |
| logarithm_glcm_SumAverage | 0.860456182 |
| original_ngtdm_Busyness | 0.860360144 |
| logarithm_glrlm_HighGrayLevelRunEmphasis | 0.860264106 |
| logarithm_glrlm_ShortRunHighGrayLevelEmphasis | 0.859591837 |
| log.sigma.4.5.mm.3D_glcm_DifferenceVariance | 0.858439376 |
| logarithm_glszm_HighGrayLevelZoneEmphasis | 0.858343337 |
| log.sigma.1.5.mm.3D_glcm_InverseVariance | 0.858055222 |
| original_gldm_DependenceNonUniformityNormalized | 0.857382953 |
| square_glrlm_LongRunHighGrayLevelEmphasis | 0.857286915 |
| wavelet.LLH_ngtdm_Busyness | 0.857190876 |
| log.sigma.3.5.mm.3D_firstorder_Uniformity | 0.856806723 |
| logarithm_glcm_Autocorrelation | 0.856614646 |
| logarithm_glszm_SmallAreaHighGrayLevelEmphasis | 0.856230492 |
| log.sigma.1.5.mm.3D_firstorder_90Percentile | 0.856134454 |
| log.sigma.3.5.mm.3D_glrlm_ShortRunLowGrayLevelEmphasis | 0.855654262 |
| wavelet.LLL_firstorder_Energy | 0.854885954 |
| wavelet.LLL_firstorder_TotalEnergy | 0.854885954 |
| original_glszm_LargeAreaLowGrayLevelEmphasis | 0.854117647 |
| log.sigma.3.5.mm.3D_firstorder_InterquartileRange | 0.854021609 |
| exponential_gldm_DependenceVariance | 0.853541417 |
| log.sigma.3.5.mm.3D_gldm_LowGrayLevelEmphasis | 0.853445378 |
| log.sigma.0.5.mm.3D_gldm_DependenceVariance | 0.853061224 |
| log.sigma.3.5.mm.3D_glrlm_GrayLevelVariance | 0.852869148 |
| log.sigma.2.5.mm.3D_glcm_JointAverage | 0.852484994 |
| log.sigma.2.5.mm.3D_glcm_SumAverage | 0.852484994 |
| log.sigma.3.5.mm.3D_firstorder_RobustMeanAbsoluteDeviation | 0.852292917 |
| log.sigma.1.5.mm.3D_glcm_Correlation | 0.851812725 |
| squareroot_gldm_SmallDependenceLowGrayLevelEmphasis | 0.851620648 |
| wavelet.LLH_gldm_LargeDependenceEmphasis | 0.85152461 |
| log.sigma.3.5.mm.3D_firstorder_MeanAbsoluteDeviation | 0.85152461 |
| squareroot_glszm_LargeAreaLowGrayLevelEmphasis | 0.851332533 |
| original_firstorder_RootMeanSquared | 0.851236495 |
| wavelet.HLH_glszm_LargeAreaHighGrayLevelEmphasis | 0.851140456 |
| wavelet.LHH_ngtdm_Contrast | 0.851044418 |
| exponential_glrlm_RunPercentage | 0.850852341 |
| logarithm_gldm_LowGrayLevelEmphasis | 0.850564226 |
| square_firstorder_Mean | 0.85027611 |
| log.sigma.2.5.mm.3D_firstorder_Mean | 0.849987995 |
| log.sigma.3.5.mm.3D_glrlm_GrayLevelNonUniformityNormalized | 0.849507803 |
| exponential_gldm_LargeDependenceEmphasis | 0.849315726 |
| exponential_gldm_LargeDependenceHighGrayLevelEmphasis | 0.849315726 |
| exponential_gldm_LargeDependenceLowGrayLevelEmphasis | 0.849315726 |
| square_gldm_DependenceVariance | 0.848835534 |
| wavelet.LLH_glrlm_RunVariance | 0.848739496 |
| logarithm_glrlm_ShortRunLowGrayLevelEmphasis | 0.847971188 |
| wavelet.LLH_glrlm_RunPercentage | 0.847971188 |
| original_shape_SurfaceVolumeRatio | 0.84787515 |
| logarithm_firstorder_Mean | 0.847683073 |
| squareroot_glszm_ZonePercentage | 0.847587035 |
| logarithm_glrlm_LowGrayLevelRunEmphasis | 0.847587035 |
| wavelet.LLH_glrlm_LongRunEmphasis | 0.846818727 |
| logarithm_glrlm_LongRunLowGrayLevelEmphasis | 0.84605042 |
| wavelet.LHL_firstorder_Energy | 0.845954382 |
| wavelet.LHL_firstorder_TotalEnergy | 0.845954382 |
| log.sigma.0.5.mm.3D_firstorder_Skewness | 0.845378151 |
| wavelet.HLL_glszm_SizeZoneNonUniformity | 0.845090036 |
| logarithm_firstorder_RootMeanSquared | 0.844993998 |
| log.sigma.0.5.mm.3D_gldm_DependenceNonUniformityNormalized | 0.844993998 |
| log.sigma.1.5.mm.3D_ngtdm_Busyness | 0.844129652 |
| wavelet.LLH_glrlm_ShortRunEmphasis | 0.84364946 |
| wavelet.LLL_ngtdm_Strength | 0.843265306 |
| square_ngtdm_Contrast | 0.842593037 |
| log.sigma.3.5.mm.3D_firstorder_Entropy | 0.842593037 |
| squareroot_gldm_SmallDependenceEmphasis | 0.84240096 |
| original_firstorder_Median | 0.842263339 |
| log.sigma.3.5.mm.3D_glcm_Imc2 | 0.842208884 |
| wavelet.LHL_firstorder_Median | 0.841632653 |
| log.sigma.2.5.mm.3D_gldm_LowGrayLevelEmphasis | 0.841344538 |
| exponential_gldm_DependenceNonUniformityNormalized | 0.840864346 |
| exponential_glrlm_RunLengthNonUniformityNormalized | 0.840672269 |
| wavelet.HLL_gldm_LargeDependenceHighGrayLevelEmphasis | 0.840384154 |
| wavelet.LLH_glrlm_RunLengthNonUniformityNormalized | 0.839231693 |
| square_gldm_SmallDependenceLowGrayLevelEmphasis | 0.838655462 |
| square_gldm_DependenceNonUniformityNormalized | 0.838367347 |
| exponential_firstorder_MeanAbsoluteDeviation | 0.837983193 |
| log.sigma.4.5.mm.3D_glrlm_LongRunHighGrayLevelEmphasis | 0.837983193 |
| log.sigma.0.5.mm.3D_glcm_Imc2 | 0.837503001 |
| logarithm_glszm_LowGrayLevelZoneEmphasis | 0.836926771 |
| wavelet.LLL_glcm_Idmn | 0.836926771 |
| squareroot_glrlm_ShortRunLowGrayLevelEmphasis | 0.836254502 |
| exponential_gldm_DependenceEntropy | 0.835870348 |
| original_glcm_DifferenceEntropy | 0.834717887 |
| wavelet.LLL_gldm_SmallDependenceEmphasis | 0.833373349 |
| log.sigma.3.5.mm.3D_glcm_Correlation | 0.833085234 |
| square_glrlm_RunLengthNonUniformity | 0.832797119 |
| log.sigma.4.5.mm.3D_firstorder_10Percentile | 0.83270108 |
| wavelet.LLH_glszm_LargeAreaHighGrayLevelEmphasis | 0.832605042 |
| square_firstorder_90Percentile | 0.832220888 |
| logarithm_glszm_SmallAreaLowGrayLevelEmphasis | 0.831836735 |
| log.sigma.2.5.mm.3D_glcm_Autocorrelation | 0.831164466 |
| exponential_firstorder_RootMeanSquared | 0.830684274 |
| log.sigma.1.5.mm.3D_ngtdm_Strength | 0.829819928 |
| wavelet.HLH_ngtdm_Contrast | 0.829243697 |
| wavelet.LLH_firstorder_Entropy | 0.828955582 |
| wavelet.LLH_glrlm_GrayLevelNonUniformityNormalized | 0.82847539 |
| square_firstorder_10Percentile | 0.828091236 |
| wavelet.LHL_glszm_ZoneVariance | 0.827803121 |
| wavelet.LLH_firstorder_RobustMeanAbsoluteDeviation | 0.827707083 |
| wavelet.HHL_glszm_SizeZoneNonUniformity | 0.826362545 |
| log.sigma.3.5.mm.3D_firstorder_RootMeanSquared | 0.826362545 |
| log.sigma.2.5.mm.3D_firstorder_90Percentile | 0.825882353 |
| wavelet.HHL_ngtdm_Contrast | 0.825806142 |
| log.sigma.3.5.mm.3D_firstorder_Minimum | 0.825402161 |
| log.sigma.1.5.mm.3D_firstorder_RobustMeanAbsoluteDeviation | 0.825018007 |
| wavelet.LLH_glcm_Idm | 0.82482593 |
| wavelet.HHH_glrlm_GrayLevelNonUniformity | 0.824729892 |
| log.sigma.1.5.mm.3D_gldm_DependenceEntropy | 0.824441777 |
| wavelet.LLH_glcm_DifferenceEntropy | 0.824057623 |
| wavelet.LLH_gldm_DependenceNonUniformityNormalized | 0.823961585 |
| log.sigma.2.5.mm.3D_firstorder_Variance | 0.823769508 |
| wavelet.LHL_glszm_LargeAreaEmphasis | 0.8230012 |
| log.sigma.2.5.mm.3D_firstorder_MeanAbsoluteDeviation | 0.822713085 |
| wavelet.LLH_glcm_Id | 0.822617047 |
| log.sigma.2.5.mm.3D_glrlm_ShortRunLowGrayLevelEmphasis | 0.822617047 |
| wavelet.LLL_ngtdm_Busyness | 0.82242497 |
| log.sigma.1.5.mm.3D_firstorder_InterquartileRange | 0.821944778 |
| log.sigma.2.5.mm.3D_gldm_GrayLevelVariance | 0.821080432 |
| squareroot_glrlm_LowGrayLevelRunEmphasis | 0.820792317 |
| log.sigma.0.5.mm.3D_gldm_DependenceEntropy | 0.820792317 |
| wavelet.LLH_firstorder_Uniformity | 0.820504202 |
| wavelet.LLH_glcm_DifferenceAverage | 0.819831933 |
| log.sigma.2.5.mm.3D_glrlm_LowGrayLevelRunEmphasis | 0.819255702 |
| wavelet.LHL_firstorder_Mean | 0.819159664 |
| squareroot_glcm_Imc1 | 0.818391357 |
| log.sigma.0.5.mm.3D_glszm_GrayLevelNonUniformityNormalized | 0.818103241 |
| log.sigma.3.5.mm.3D_glrlm_LowGrayLevelRunEmphasis | 0.818103241 |
| original_firstorder_RobustMeanAbsoluteDeviation | 0.817719088 |
| wavelet.LLL_glszm_ZoneVariance | 0.817623049 |
| wavelet.LLL_glszm_ZonePercentage | 0.817430972 |
| log.sigma.0.5.mm.3D_gldm_LargeDependenceHighGrayLevelEmphasis | 0.817334934 |
| wavelet.LLL_glszm_LargeAreaEmphasis | 0.81695078 |
| wavelet.LLL_glszm_LargeAreaHighGrayLevelEmphasis | 0.816758703 |
| logarithm_glcm_Imc2 | 0.816566627 |
| squareroot_glcm_Idn | 0.816470588 |
| log.sigma.3.5.mm.3D_firstorder_Median | 0.816278511 |
| square_firstorder_Energy | 0.815606242 |
| square_firstorder_TotalEnergy | 0.815606242 |
| log.sigma.2.5.mm.3D_glcm_SumSquares | 0.815510204 |
| squareroot_gldm_LowGrayLevelEmphasis | 0.815414166 |
| wavelet.LLL_glszm_SmallAreaLowGrayLevelEmphasis | 0.814741897 |
| wavelet.LLH_glcm_SumEntropy | 0.813973589 |
| square_firstorder_MeanAbsoluteDeviation | 0.813877551 |
| square_gldm_SmallDependenceEmphasis | 0.813493397 |
| log.sigma.2.5.mm.3D_firstorder_Entropy | 0.813205282 |
| wavelet.HHL_glrlm_GrayLevelNonUniformity | 0.813013205 |
| wavelet.LLH_glcm_InverseVariance | 0.813013205 |
| wavelet.LHL_ngtdm_Complexity | 0.812533013 |
| wavelet.LLH_glcm_JointEntropy | 0.812244898 |
| wavelet.LLH_firstorder_MeanAbsoluteDeviation | 0.811860744 |
| log.sigma.2.5.mm.3D_gldm_SmallDependenceHighGrayLevelEmphasis | 0.811860744 |
| log.sigma.0.5.mm.3D_glcm_ClusterProminence | 0.811668667 |
| original_glcm_InverseVariance | 0.81090036 |
| log.sigma.4.5.mm.3D_glcm_InverseVariance | 0.810708283 |
| log.sigma.3.5.mm.3D_glcm_ClusterProminence | 0.810420168 |
| wavelet.LLH_firstorder_InterquartileRange | 0.81032413 |
| log.sigma.3.5.mm.3D_glcm_SumSquares | 0.807539016 |
| logarithm_gldm_LargeDependenceLowGrayLevelEmphasis | 0.8072509 |
| wavelet.HHL_glszm_LargeAreaHighGrayLevelEmphasis | 0.807154862 |
| wavelet.LLL_glcm_Idn | 0.806386555 |
| original_glcm_Idmn | 0.806002401 |
| exponential_firstorder_Variance | 0.804657863 |
| wavelet.HHH_gldm_GrayLevelNonUniformity | 0.804561825 |
| log.sigma.0.5.mm.3D_glcm_Imc1 | 0.804561825 |
| exponential_firstorder_Median | 0.803793517 |
| log.sigma.4.5.mm.3D_gldm_LowGrayLevelEmphasis | 0.803313325 |
| square_glszm_ZonePercentage | 0.802160864 |
| log.sigma.3.5.mm.3D_glcm_JointAverage | 0.801392557 |
| log.sigma.3.5.mm.3D_glcm_SumAverage | 0.801392557 |
| logarithm_firstorder_Maximum | 0.800336134 |

**Supplementary Table 3.** Univariate Cox regression analysis of the clinical factors and progressive-free survival

| **Characteristic** | **Hazard ratio** | **95% confidence interval** | **p-value** |
| --- | --- | --- | --- |
| cT | - | - | - |
| 1 | Reference | - | - |
| 2 | 2.24 | 0.9-5.58 | 0.082 |
| 3 | 1.66 | 0.45-6.08 | 0.444 |
| 4 | 1.9 | 0.77-4.69 | 0.162 |
| cN | - | - | - |
| 0 | Reference | - | - |
| 1 | 1.39 | 0.98-1.96 | 0.062 |
| cM | - | - | - |
| 0 | Reference | - | - |
| 1 | 4.02 | 1.96-8.28 | <0.001 |
| Smoking Status | - | - | - |
| No | Reference | - | - |
| Yes | 1.26 | 0.44-3.6 | 0.673 |
| Lesion location | 1.18 | 0.89-1.56 | 0.247 |
| Lesion condition | - | - | - |
| Subsolid | Reference | - | - |
| Solid | 3.85 | 1.57-9.4 | 0.003 |
| Pathology | - | - | - |
| Non-invasive | Reference | - | - |
| Microinvasive | 0.16 | 0.04-0.66 | 0.011 |
| Invasive | 0.35 | 0.14-0.88 | 0.025 |
| Sex |  |  |  |
| Female | Reference | - | - |
| Male | 2.07 | 0.97-4.41 | 0.058 |
| Age | 1.02 | 0.98-1.06 | 0.280 |

**Supplementary Table 4.** Pairwise comparison of C-index in R-, C-, and Combi-models

|  | **Train cohort** | | |  | **External test cohort** | | | |
| --- | --- | --- | --- | --- | --- | --- | --- | --- |
|  | **R-model** | **C-model** | **Combi-model** |  | **R-model** | | **C-model** | **Combi-model** |
| C-index | 0.781 | 0.755 | 0.845 |  | 0.778 | | 0.739 | 0.837 |
| Variance | 0.0013 | 0.0023 | 0.0009 |  | 0.0015 | | 0.003 | 0.0024 |
| p-value | 0.584 | |  |  | 0.740 | | |  |
|  |  | 0.049 | |  |  | 0.008 | | |
|  | 0.080 | | |  | 0.003 | | | |

Variance represents the estimated variance of the C-index. The p-value was calculated by Student *t* test for dependent samples.

**Supplementary Fig.1**  Kaplan-Meier curves between the follow-up time to progress-free survival

| 1. Age   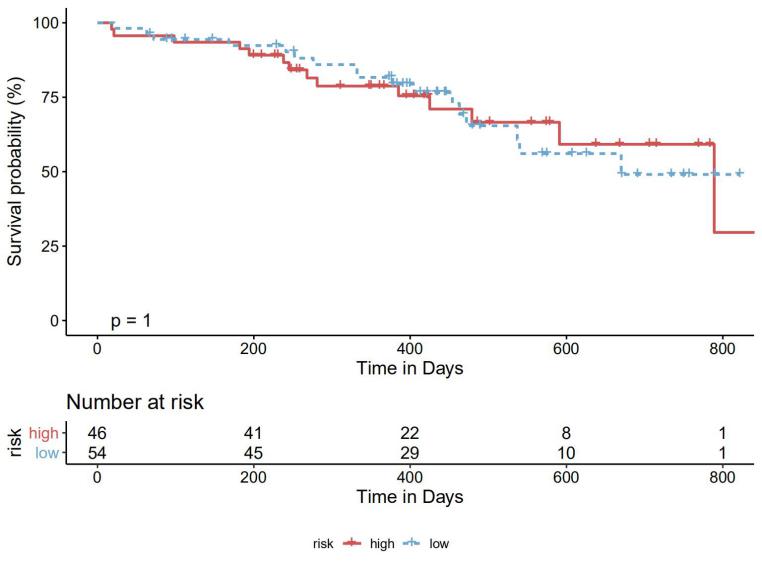 | 1. Sex   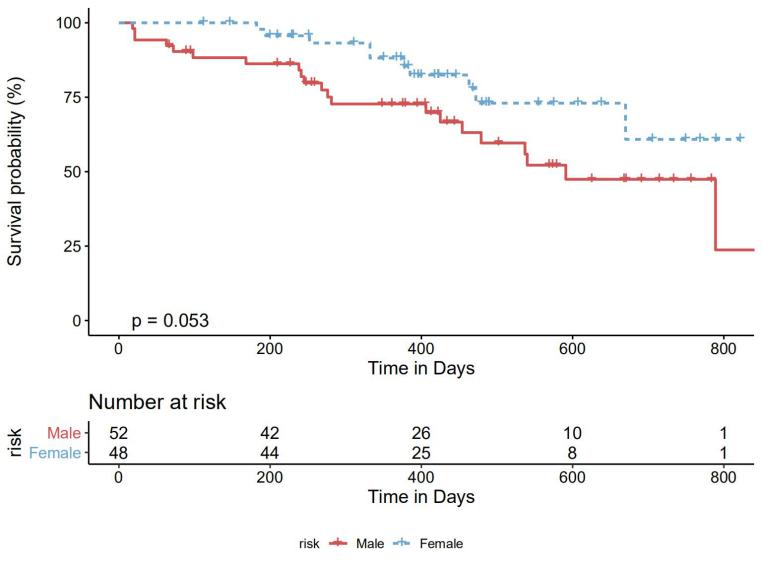 |
| --- | --- |
| 1. Smoking status   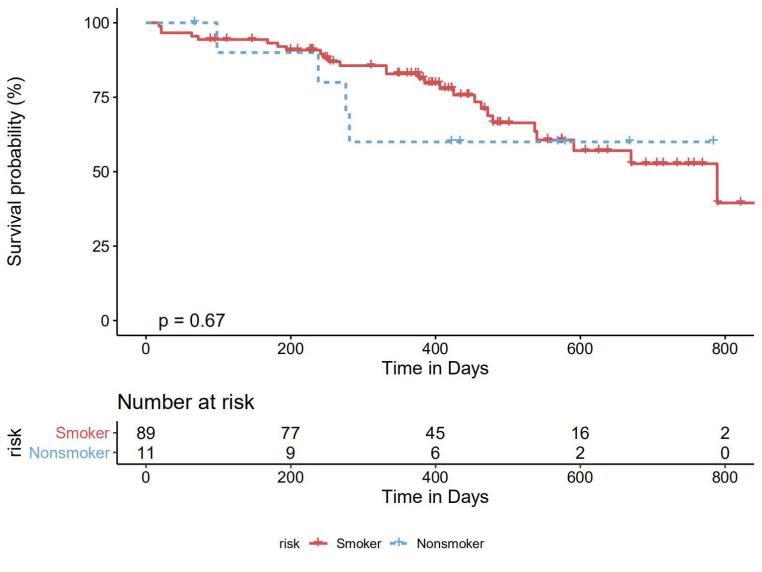 | 1. Lung nodule type   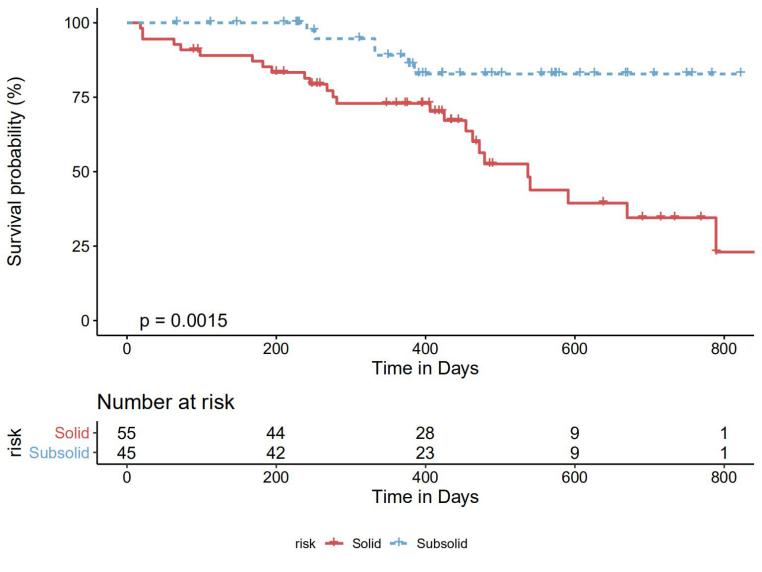 |
| 1. Lesion location   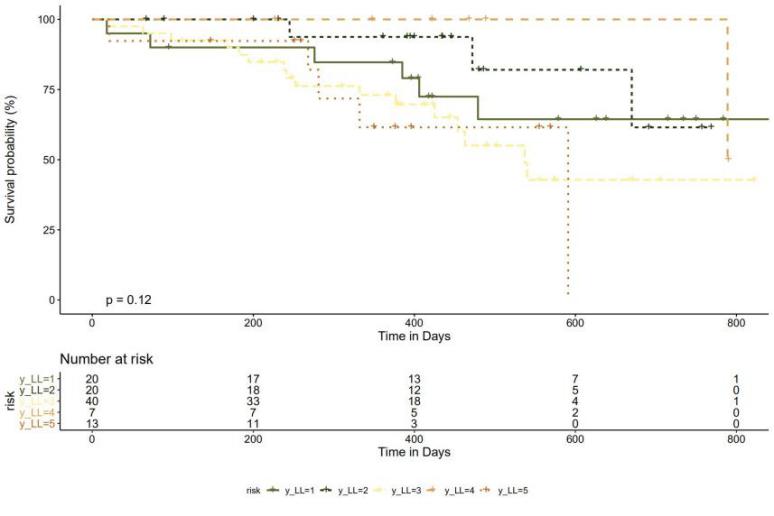 | 1. Histological type   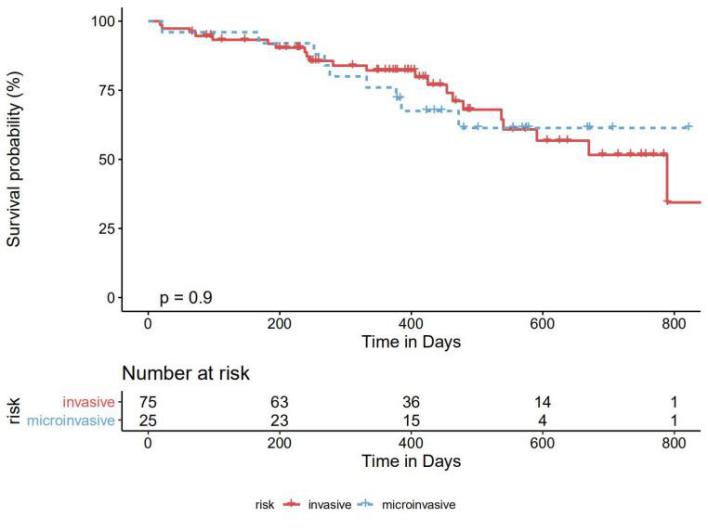 |
| 1. T stage   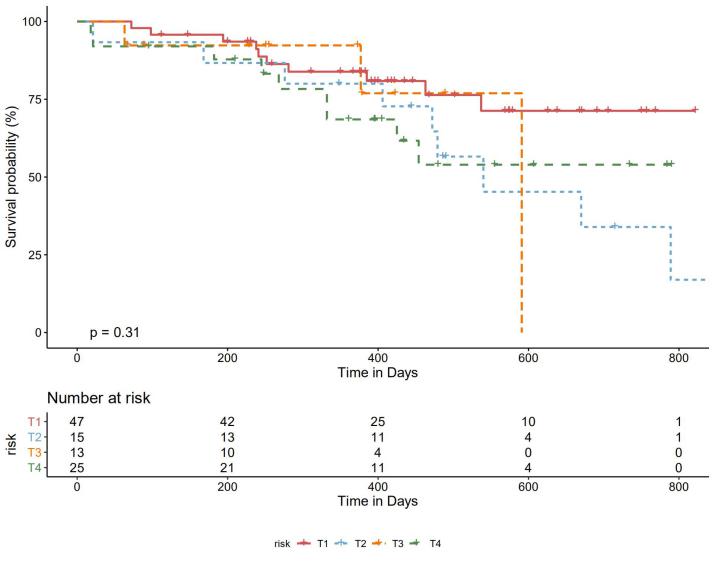 | 1. N stage   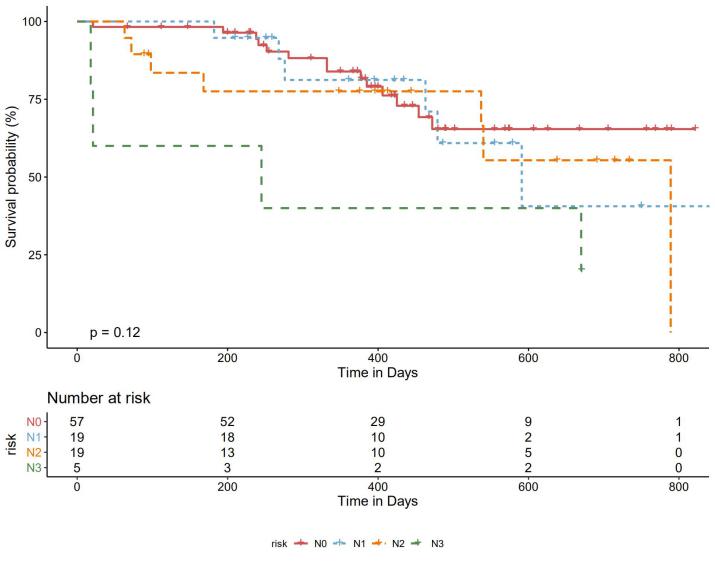 |
| 1. M stage   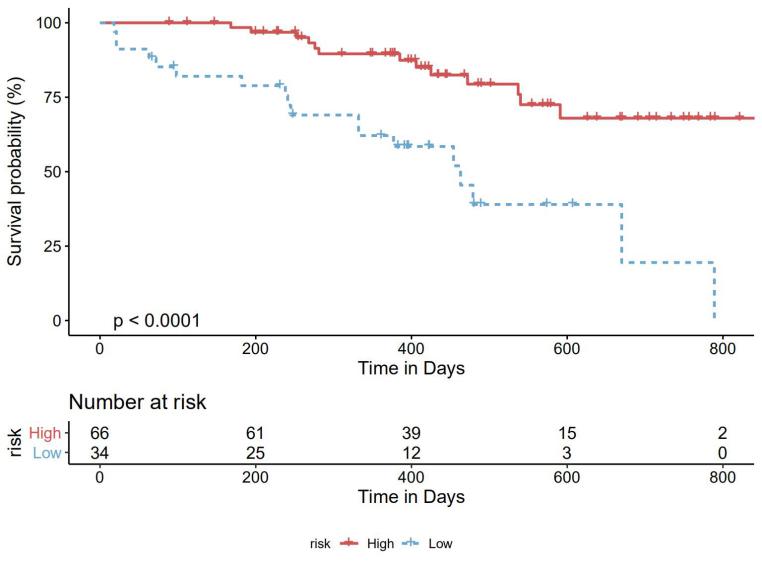 |  |

**Supplementary Fig.2** A Schoenfeld individual test for verifying whether R-model fitted for the survival analysis between the radiomic features and progression free survival


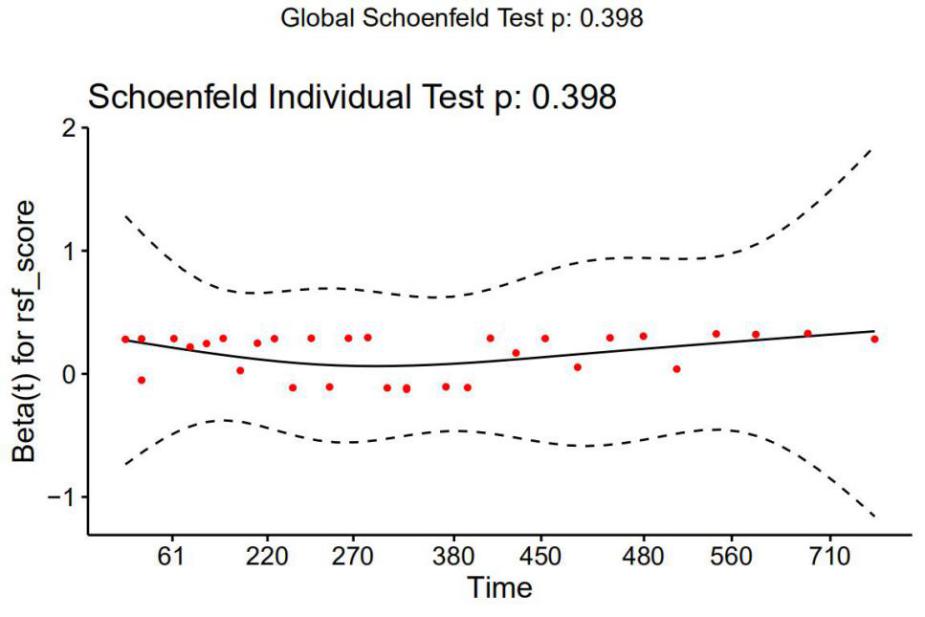


**Supplementary Fig.3** Feature importance in R-model: the most-contributive 20 features to progression free survival. The higher the importance coefficient, the greater contribution to the model


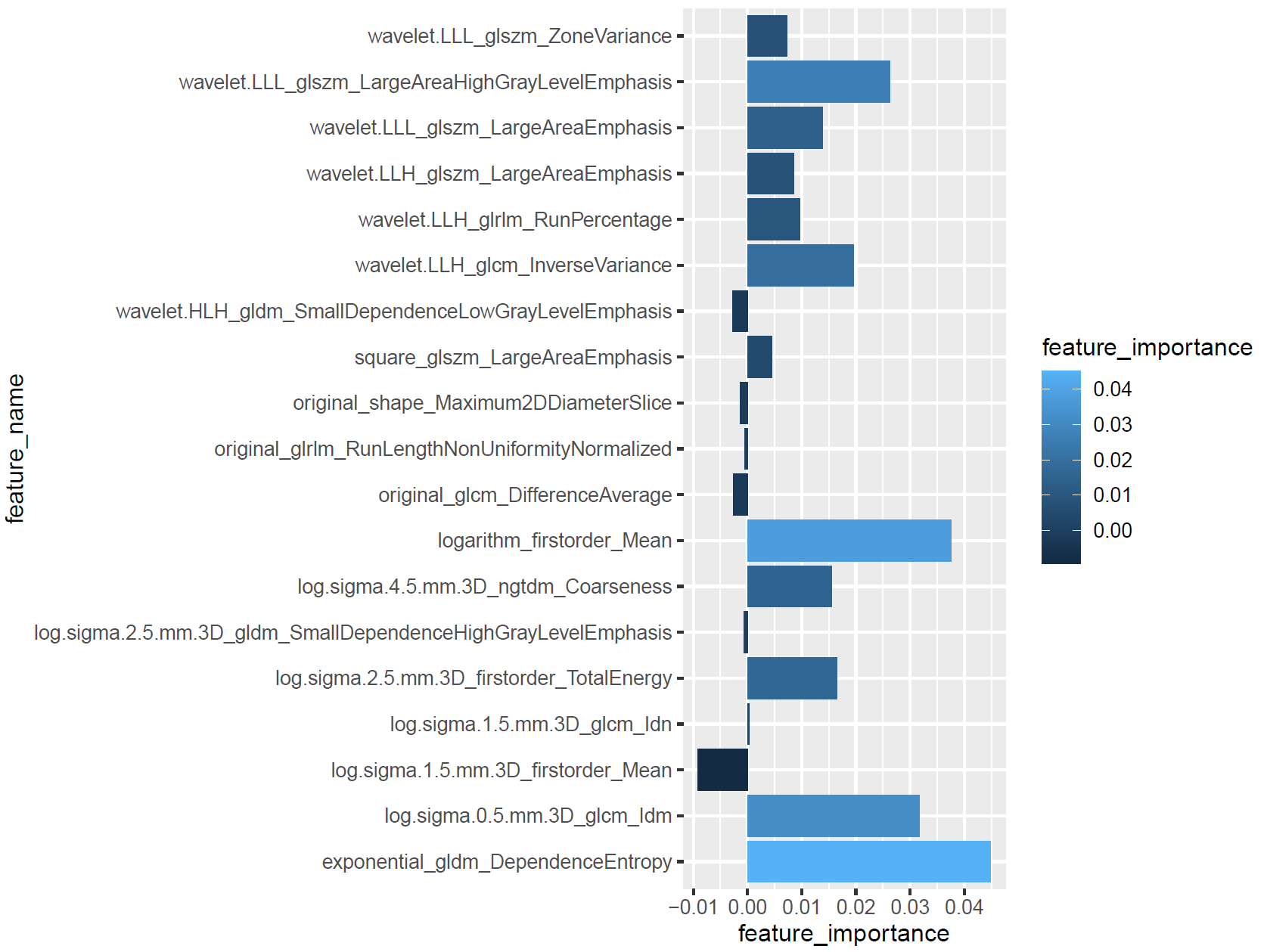


**Supplementary Fig.4** Kaplan-Meier curves of R-model

1. Training cohort


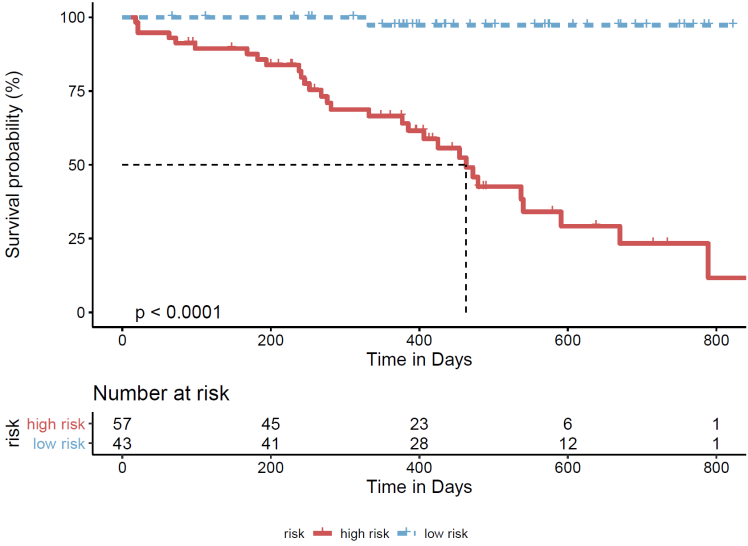


1. Internal validation cohort


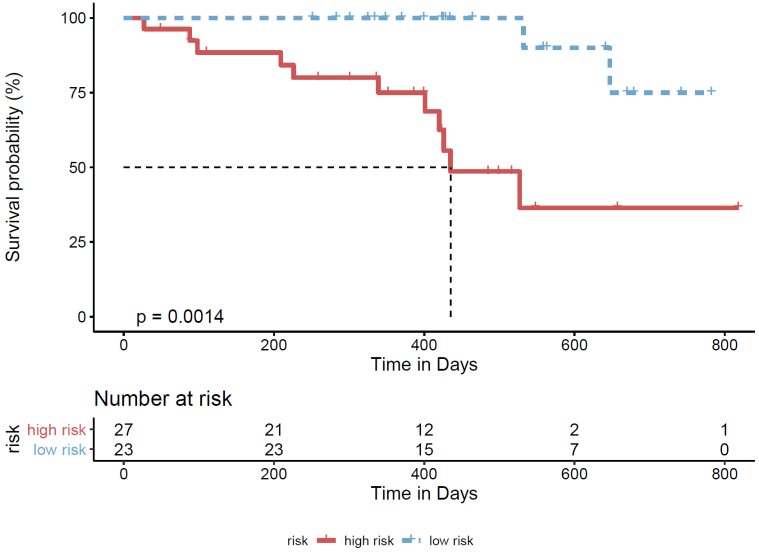


C) External test cohort


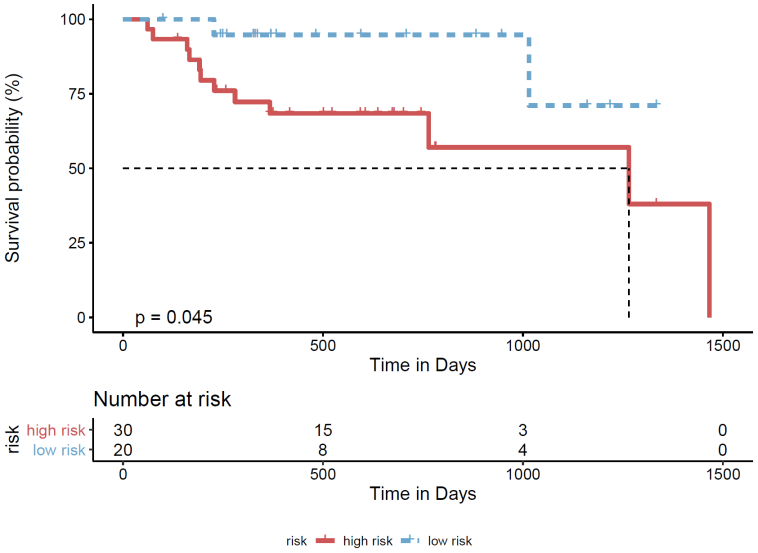


**Supplementary Fig.5** Calibration curves of R-model

1. Training cohort


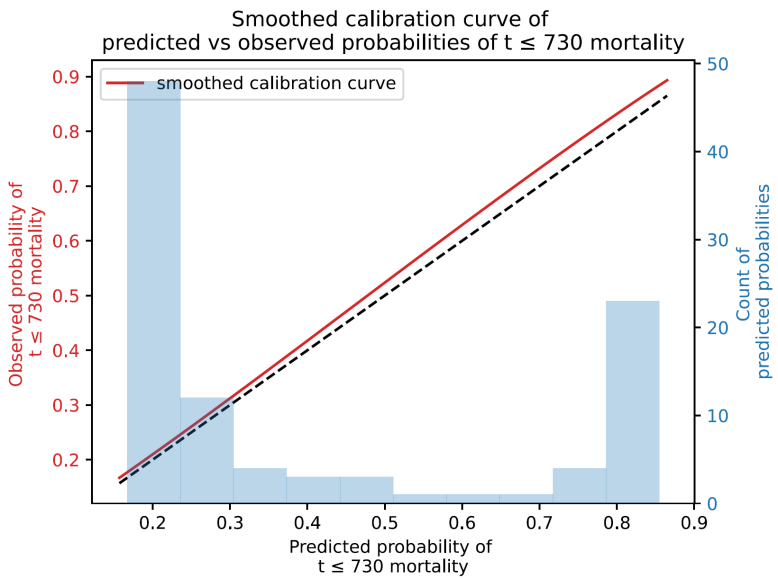


1. Internal validation cohort


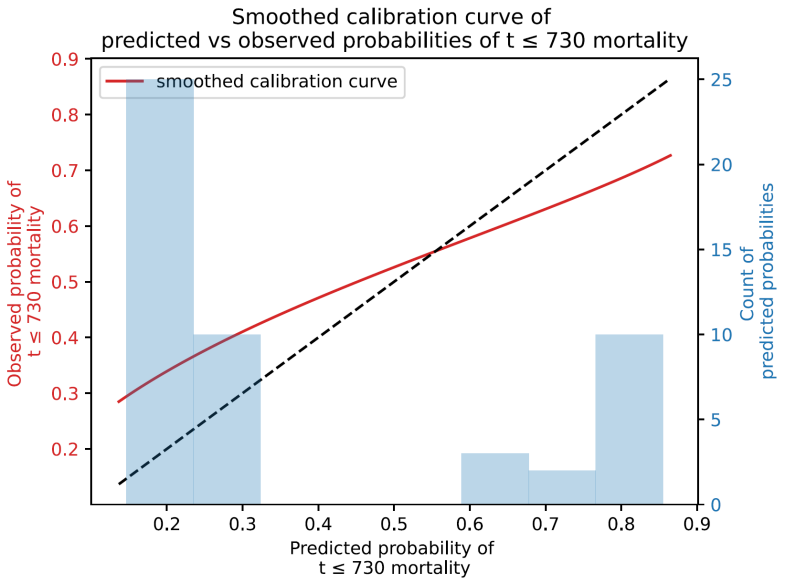


1. External test cohort


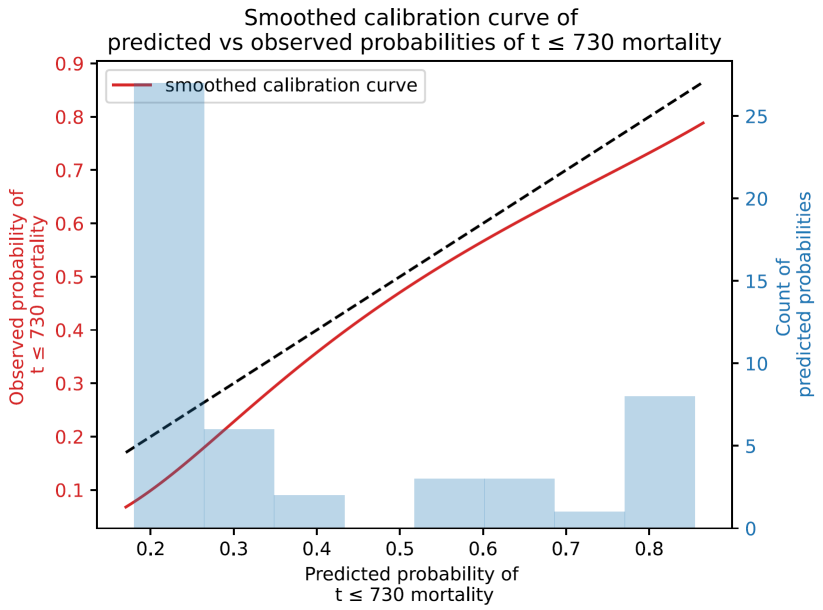


**Supplementary Fig.6** Forest plots of TNM-model. The forest plots of hazard ratio for TNM stage.


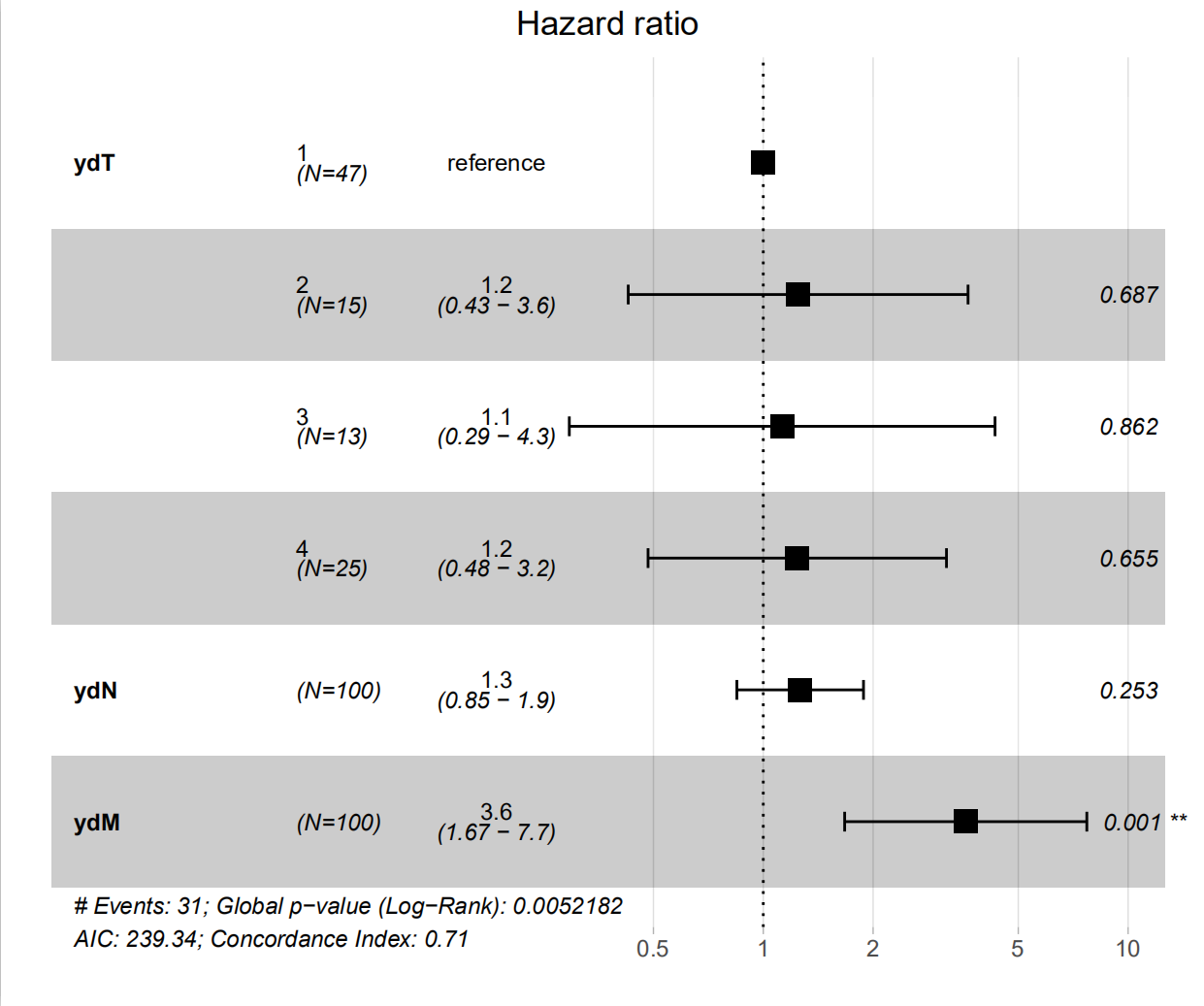


**Supplementary Fig.7**  Schoenfeld individual tests for verifying whether C-model fitted for the survival analysis between the clinical factors and progression free survival


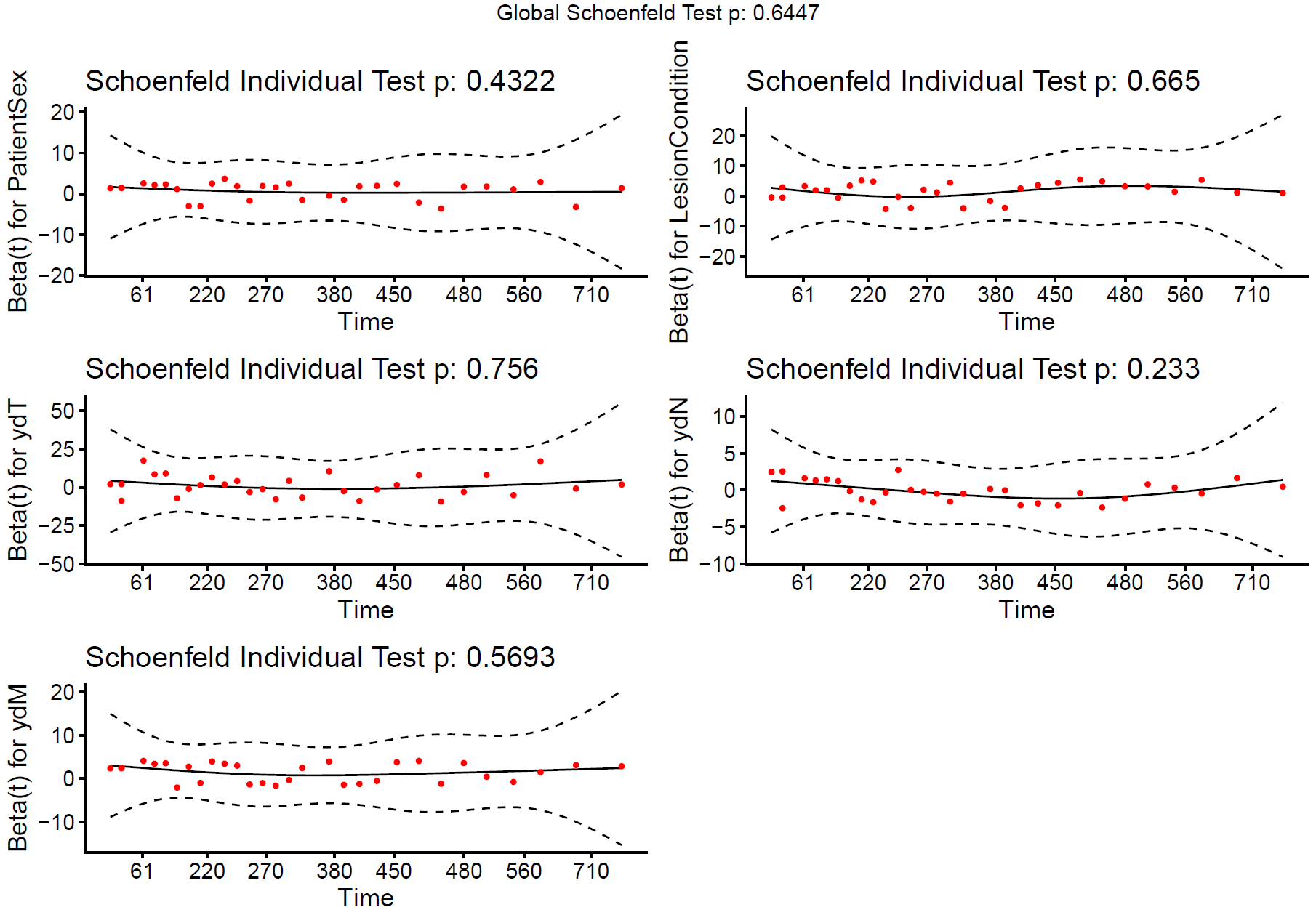


**Supplementary Fig.8** Calibration curves of C-model

1. Training cohort


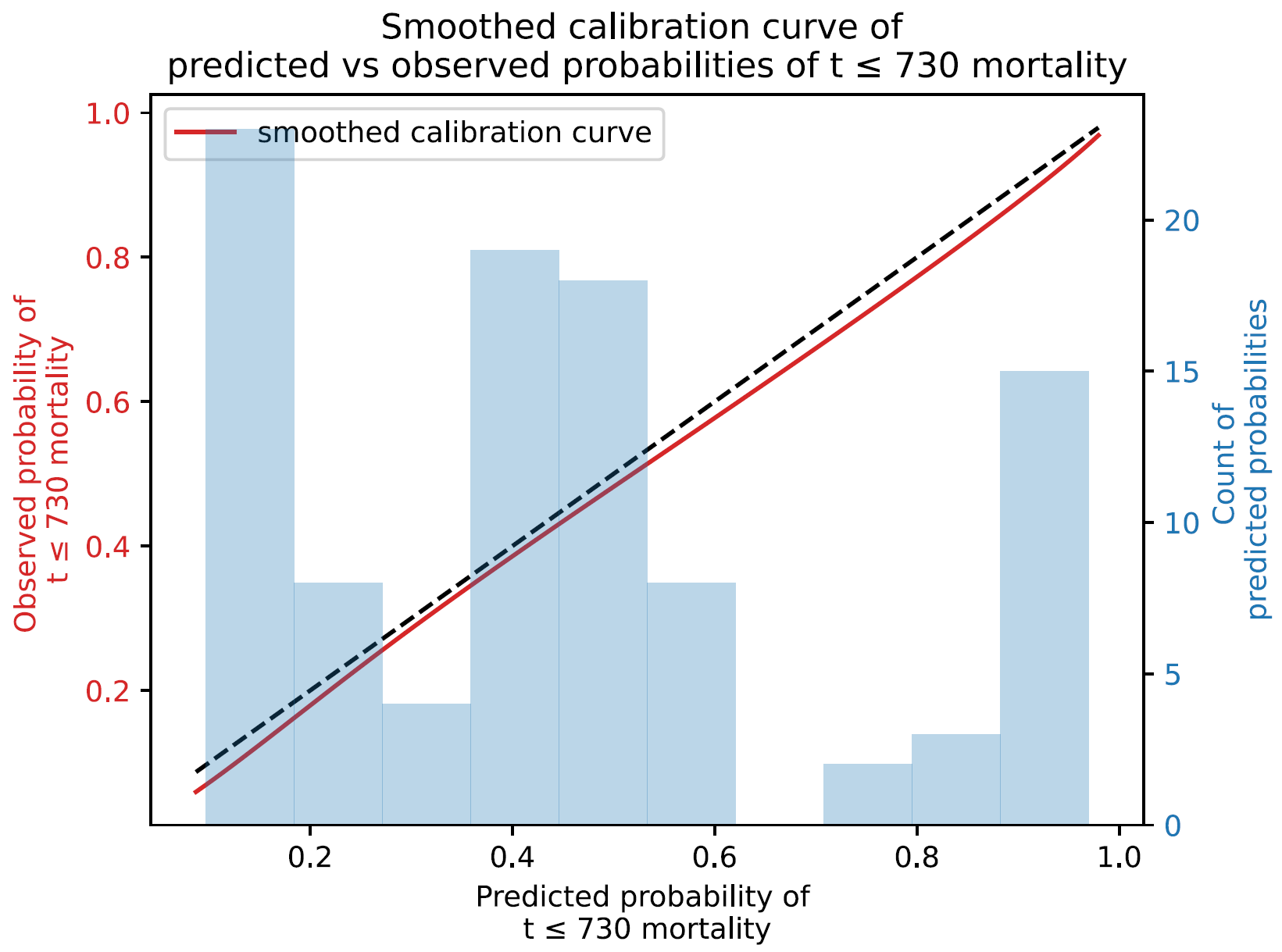


1. External test cohort


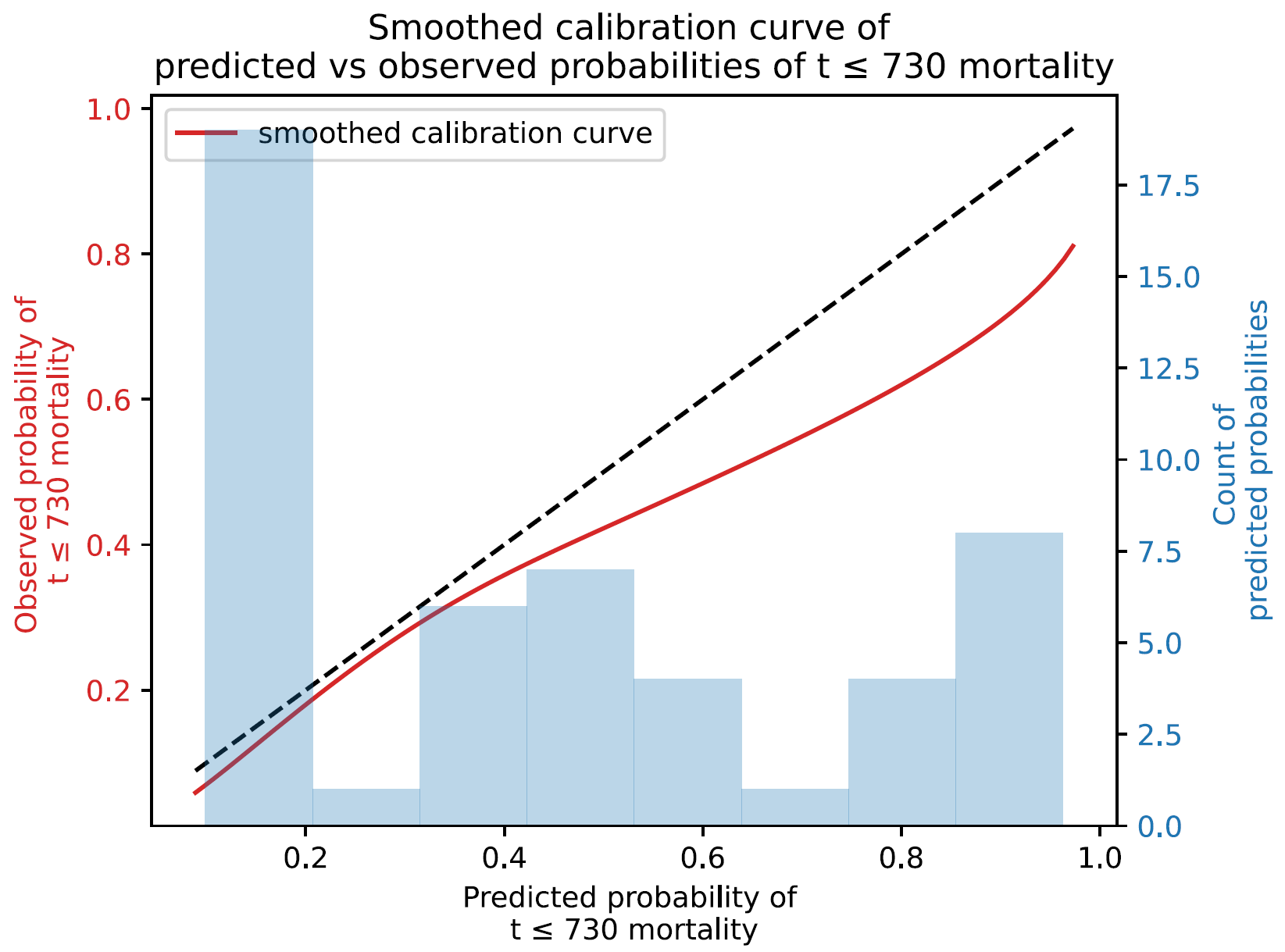


**Supplementary Fig.9** Schoenfeld individual tests for verifying whether genetic factors fitted for the survival analysis between *EGFR* mutations and progression-free survival


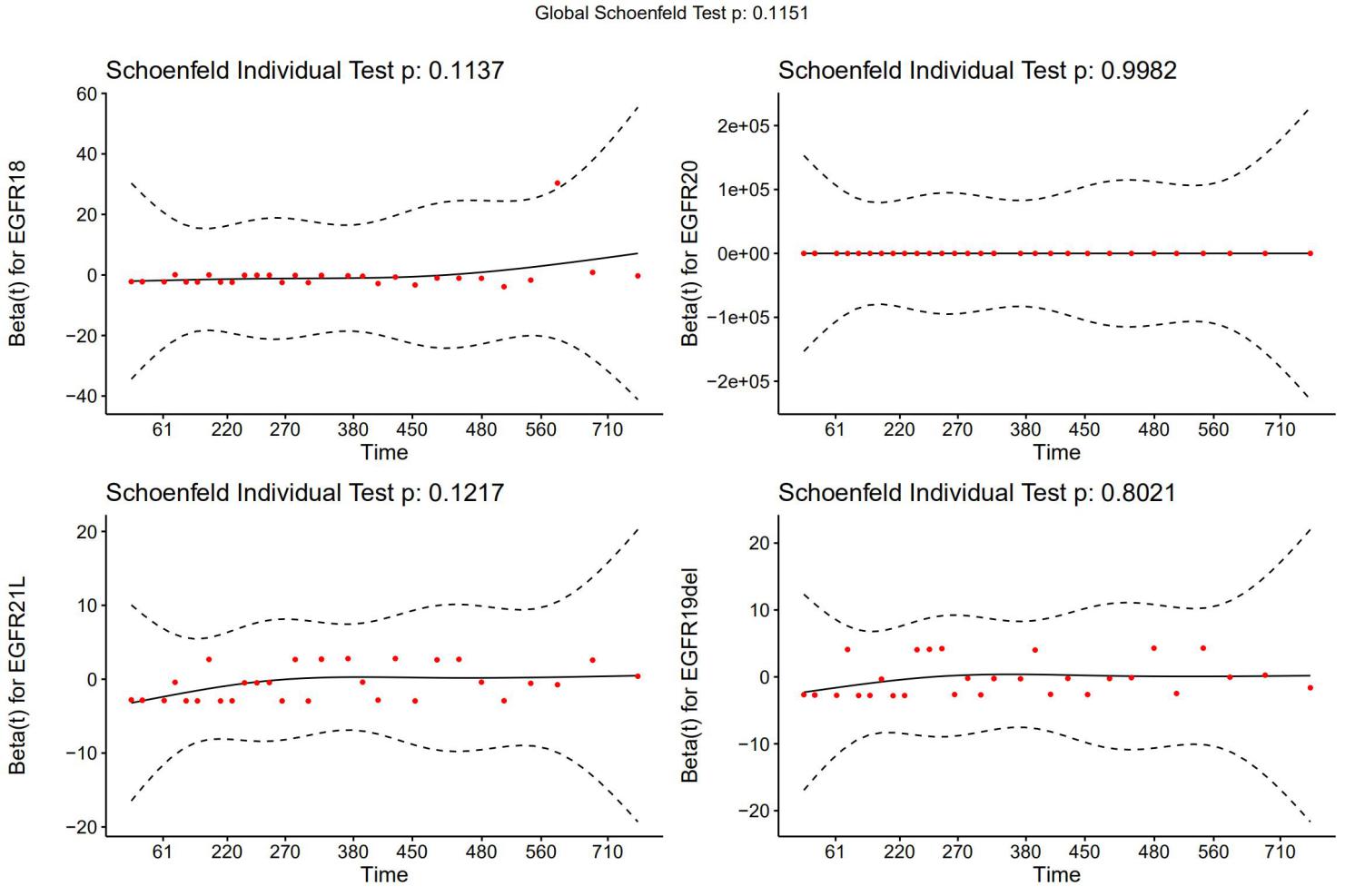


**Supplementary Fig.10** Schoenfeld individual tests for verifying whether Combi-model fitted for the survival analysis between the integrated factors and progression-free survival


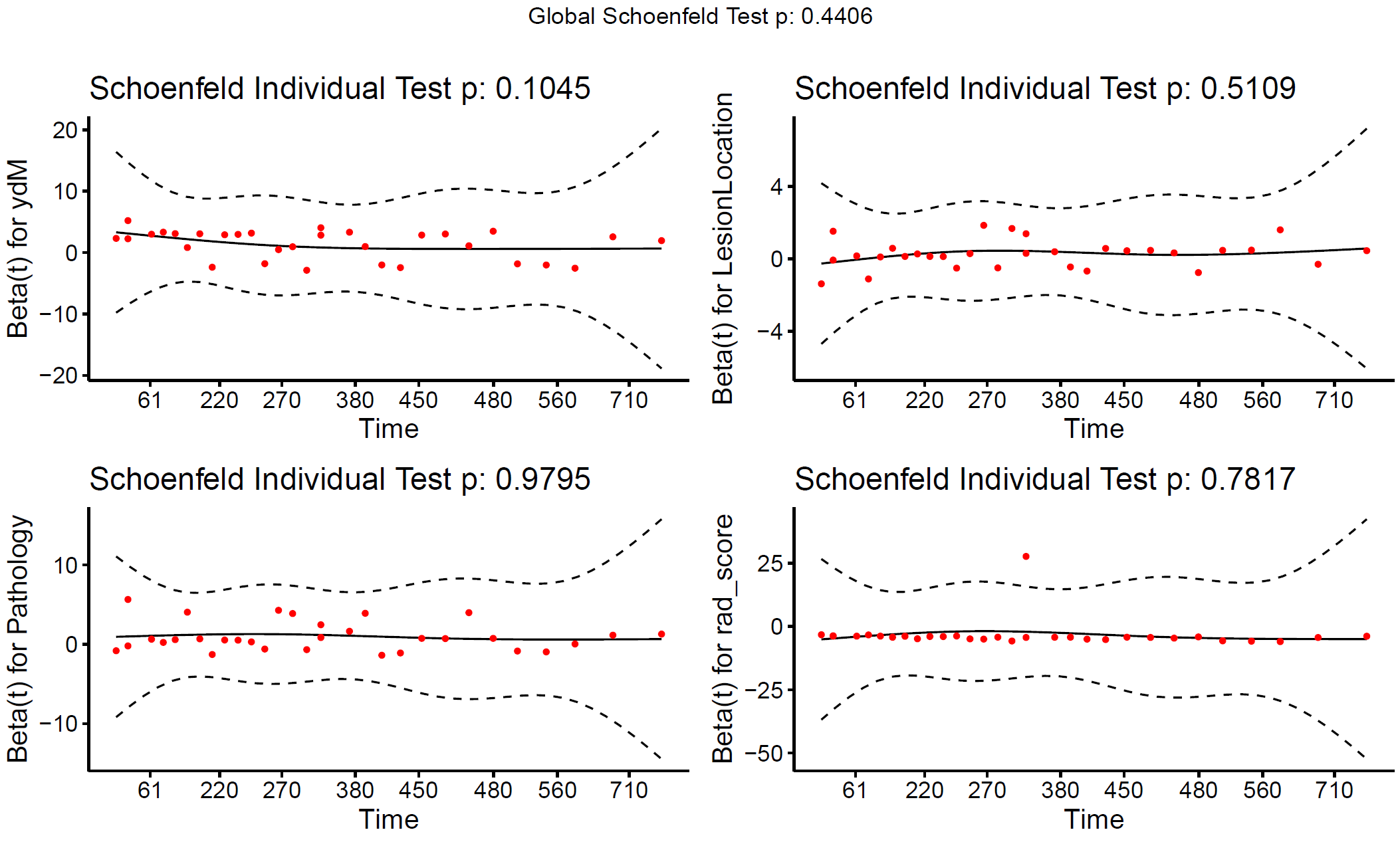


**Supplementary Fig.11** Calibration curves of Combi-model

1. Training cohort


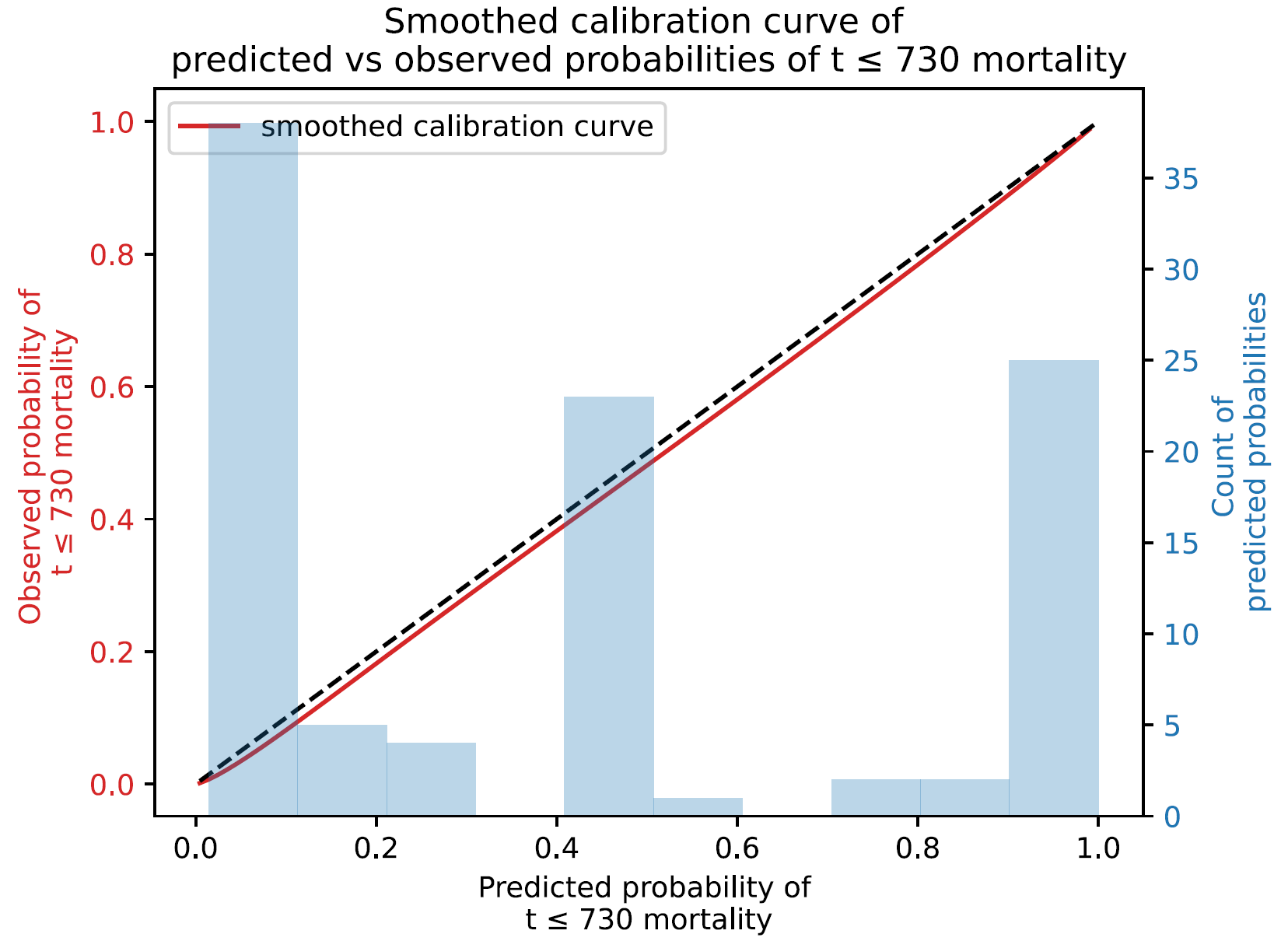


B) External test cohort


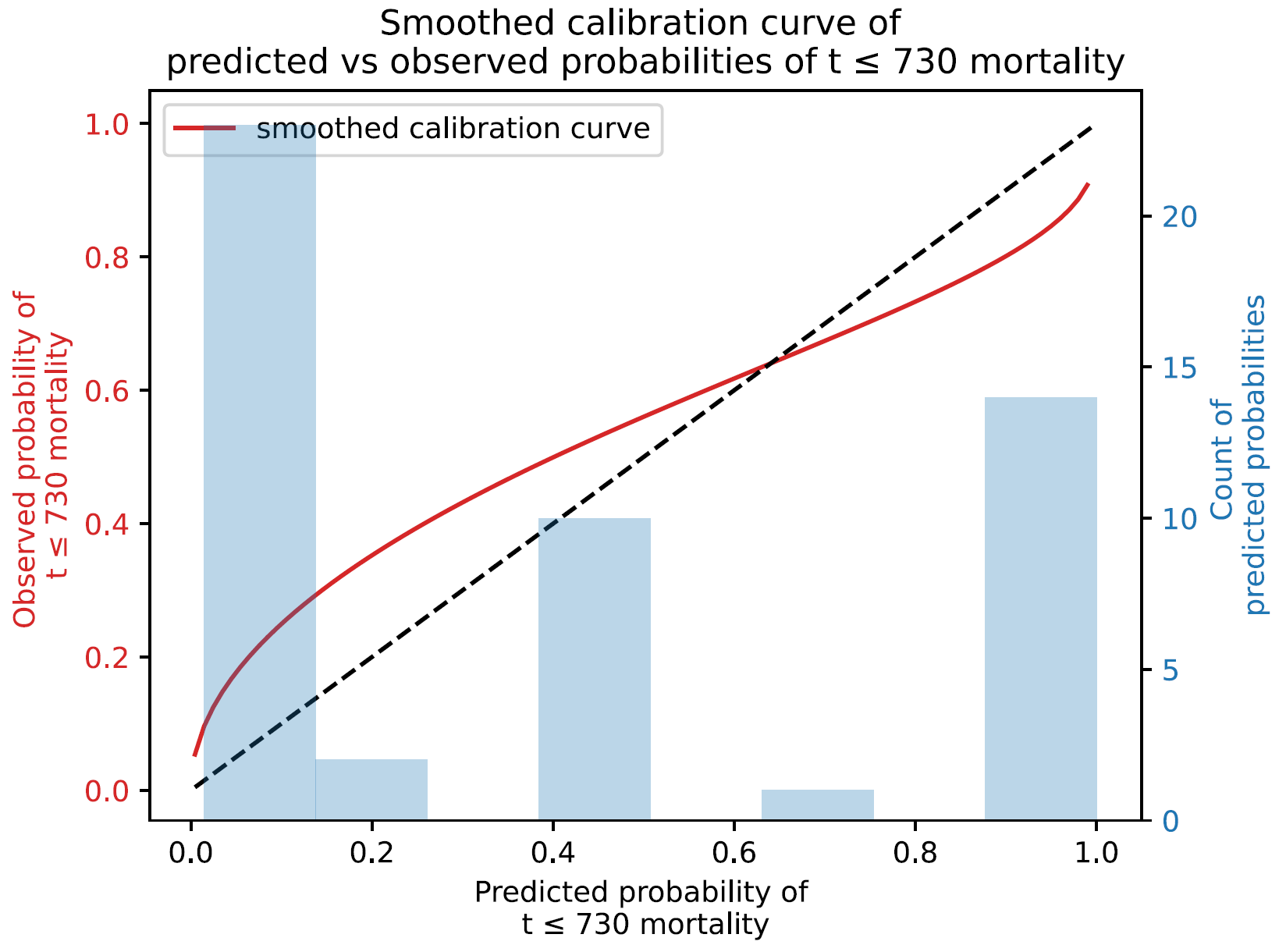


**Supplementary Fig.12** Kaplan-Meier curves between solid and nonsolid tumors

1. Solid tumors


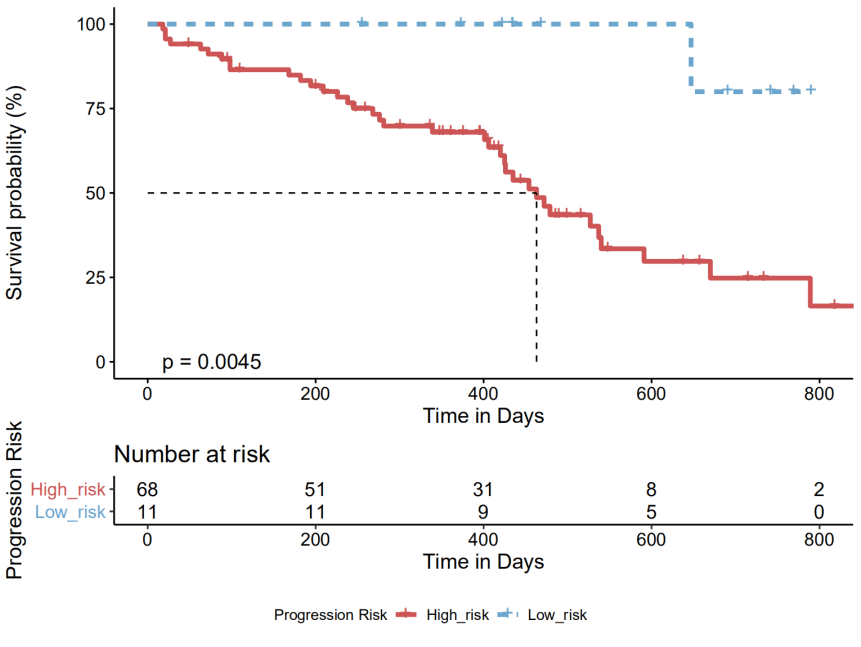


B) Subsolid tumors


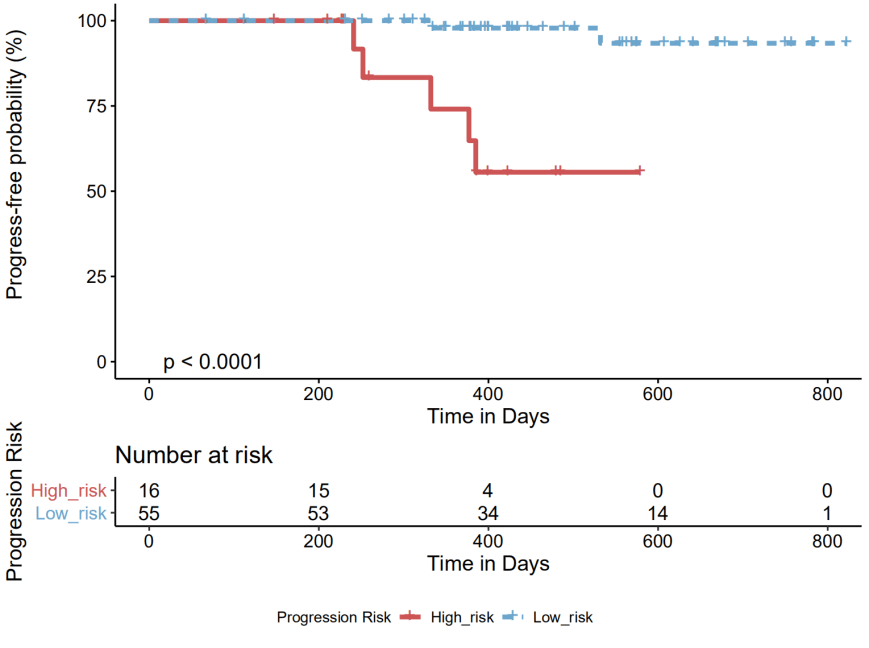


**Supplementary Methods:**

Software packages and functions used in this study:

Rad-score was generated using random survival forest method by implementing the ‘scikit-survival’ Python package ([https://scikit-survival.readthedocs.io](https://scikit-survival.readthedocs.io/en/latest/)). We used the function ’cumulative dynamic auc’ in this package to achieve the dynamic time-dependence ROC_AUC. The ‘Lifelines’ Python package ([https://lifelines.readthedocs.io](https://lifelines.readthedocs.io/en/latest/)) was applied to obtain the calibration curve for R-, C- and Combi-model. The ‘rms’ package (https://cran.r-project.org/web/packages/rms/) was applied for multivariable Cox regression, nomogram generation and calibration. The proportional hazards assumption test for all Cox models was tested by the R function ‘cox.zph’. Regarding to the comparison of C-index among all the three models, the function “concordance.index” and ‘cindex.comp’ from ‘survcomp’ package (https://cran.r-project.org/src/contrib/Archive/survcomp) were implemented. The ‘dca’ (https://cran.r-project.org/web/packages/DCA/) and ‘timeROC’ (https://cran.r-project.org/web/packages/timeROC/) packages were employed to perform the decision curve analysis and generate time-dependent ROC curve.

**Radiomic features interpretation**

1. **Major Axis**（original_shape_MajorAxis)

$$major\begin{matrix} \\ \end{matrix}axis=4\sqrt{\lambda_{major}}$$

This feature yield the largest axis length of the ROI-enclosing ellipsoid and is calculated using the largest principal component λmajorλmajor.

The principal component analysis is performed using the physical coordinates of the voxel centers defining the ROI. It therefore takes spacing into account, but does not make use of the shape mesh.

1. **Size-ZoneNon-Uniformity (SZN) (**log.sigma.0.5.mm.3D_glszm_SizeZoneNonUniformity，wavelet.LHL_glszm_SizeZoneNonUniformity，wavelet.LLL_glszm_SizeZoneNonUniformity，wavelet.HLH_glszm_SizeZoneNonUniformity，wavelet.LLH_glszm_SizeZoneNonUniformity，original_glszm_SizeZoneNonUniformity，squareroot_glszm_SizeZoneNonUniformity，log.sigma.2.5.mm.3D_glszm_SizeZoneNonUniformity，logarithm_glszm_SizeZoneNonUniformity，log.sigma.3.5.mm.3D_glszm_SizeZoneNonUniformity，log.sigma.1.5.mm.3D_glszm_SizeZoneNonUniformity，wavelet.LHH_glszm_SizeZoneNonUniformity，log.sigma.4.5.mm.3D_glszm_SizeZoneNonUniformity，wavelet.HLL_glszm_SizeZoneNonUniformity，wavelet.HHL_glszm_SizeZoneNonUniformity)

$$SZN=\frac{\sum_{j=1}^{N_{s}} (\sum_{i=1}^{N_{g}} P(i,j))^{2}}{N_{z}}$$

SZN measures the variability of size zone volumes in the image, with a lower value indicating more homogeneity in size zone volumes.

1. **LargeAreaEmphasis (LAE)** (log.sigma.4.5.mm.3D_glszm_LargeAreaEmphasis, original_glszm_LargeAreaEmphasis, log.sigma.3.5.mm.3D_glszm_LargeAreaEmphasis, log.sigma.0.5.mm.3D_glszm_LargeAreaEmphasis, log.sigma.1.5.mm.3D_glszm_LargeAreaEmphasis, log.sigma.2.5.mm.3D_glszm_LargeAreaEmphasis, squareroot_glszm_LargeAreaEmphasis, wavelet.LLH_glszm_LargeAreaEmphasis, square_glszm_LargeAreaEmphasis, logarithm_glszm_LargeAreaEmphasis, exponential_glszm_LargeAreaEmphasis, wavelet.LHL_glszm_LargeAreaEmphasis, wavelet.LLL_glszm_LargeAreaEmphasis)

$$LAE=\frac{\sum_{i=1}^{N_{g}} \sum_{j=1}^{N_{s}} P(i,j)j^{2}}{N_{z}}$$

LAE is a measure of the distribution of large area size zones, with a greater value indicative of more larger size zones and more coarse textures.

1. **Large Area High Gray Level Emphasis  (LAHGLE)** ( log.sigma.4.5.mm.3D_glszm_LargeAreaHighGrayLevelEmphasis, log.sigma.3.5.mm.3D_glszm_LargeAreaHighGrayLevelEmphasis. logarithm_glszm_LargeAreaHighGrayLevelEmphasis, log.sigma.2.5.mm.3D_glszm_LargeAreaHighGrayLevelEmphasis, squareroot_glszm_LargeAreaHighGrayLevelEmphasis, log.sigma.1.5.mm.3D_glszm_LargeAreaHighGrayLevelEmphasis, log.sigma.0.5.mm.3D_glszm_LargeAreaHighGrayLevelEmphasis, original_glszm_LargeAreaHighGrayLevelEmphasis, square_glszm_LargeAreaHighGrayLevelEmphasis, wavelet.HHH_glszm_LargeAreaHighGrayLevelEmphasis, exponential_glszm_LargeAreaHighGrayLevelEmphasis, wavelet.LHH_glszm_LargeAreaHighGrayLevelEmphasis, wavelet.HLH_glszm_LargeAreaHighGrayLevelEmphasis, wavelet.LLH_glszm_LargeAreaHighGrayLevelEmphasis, wavelet.LLL_glszm_LargeAreaHighGrayLevelEmphasis, wavelet.HHL_glszm_LargeAreaHighGrayLevelEmphasis)

$$LAHGLE=\frac{\sum_{i=1}^{N_{g}} \sum_{j=1}^{N_{s}} P(i,j)i^{2}j^{2}}{N_{z}}$$

LAHGLE measures the proportion in the image of the joint distribution of larger size zones with higher gray-level values.

1. **Inverse Difference (ID)** (log.sigma.4.5.mm.3D_glcm_Id, log.sigma.3.5.mm.3D_glcm_Id, log.sigma.2.5.mm.3D_glcm_Id, log.sigma.1.5.mm.3D_glcm_Id, log.sigma.0.5.mm.3D_glcm_Id, original_glcm_Id, wavelet.LLH_glcm_Id)

$$ID=\sum_{k=0}^{N_{g}-1} \frac{p_{x-y}(k)}{1+k}$$

ID (a.k.a. Homogeneity 1) is another measure of the local homogeneity of an image. With more uniform gray levels, the denominator will remain low, resulting in a higher overall value.

1. **Gray Level Non-Uniformity  (GLN)**  (log.sigma.0.5.mm.3D_glszm_GrayLevelNonUniformity, exponential_glrlm_GrayLevelNonUniformity, wavelet.LLH_glszm_GrayLevelNonUniformity, log.sigma.3.5.mm.3D_gldm_GrayLevelNonUniformity, square_glrlm_GrayLevelNonUniformity, logarithm_glszm_GrayLevelNonUniformity, log.sigma.4.5.mm.3D_gldm_GrayLevelNonUniformity, log.sigma.4.5.mm.3D_glrlm_GrayLevelNonUniformity, wavelet.LHL_glszm_GrayLevelNonUniformity, log.sigma.3.5.mm.3D_glrlm_GrayLevelNonUniformity, wavelet.LHH_glszm_GrayLevelNonUniformity, squareroot_glszm_GrayLevelNonUniformity, wavelet.LLH_glrlm_GrayLevelNonUniformity, original_glrlm_GrayLevelNonUniformity, square_gldm_GrayLevelNonUniformity, log.sigma.0.5.mm.3D_gldm_GrayLevelNonUniformity, wavelet.LLL_glszm_GrayLevelNonUniformity, exponential_gldm_GrayLevelNonUniformity, original_gldm_GrayLevelNonUniformity, log.sigma.0.5.mm.3D_glrlm_GrayLevelNonUniformity, logarithm_glrlm_GrayLevelNonUniformity, log.sigma.2.5.mm.3D_gldm_GrayLevelNonUniformity, log.sigma.1.5.mm.3D_gldm_GrayLevelNonUniformity, wavelet.LLH_gldm_GrayLevelNonUniformity, log.sigma.2.5.mm.3D_glrlm_GrayLevelNonUniformity, squareroot_glrlm_GrayLevelNonUniformity, log.sigma.1.5.mm.3D_glrlm_GrayLevelNonUniformity, logarithm_gldm_GrayLevelNonUniformity, original_glszm_GrayLevelNonUniformity, squareroot_gldm_GrayLevelNonUniformity, wavelet.HHL_glszm_GrayLevelNonUniformity, wavelet.HHH_glszm_GrayLevelNonUniformity, wavelet.HLH_glszm_GrayLevelNonUniformity, log.sigma.1.5.mm.3D_glszm_GrayLevelNonUniformity, wavelet.LHL_glrlm_GrayLevelNonUniformity, wavelet.LLL_glrlm_GrayLevelNonUniformity, wavelet.LHL_gldm_GrayLevelNonUniformity, log.sigma.3.5.mm.3D_glszm_GrayLevelNonUniformity, log.sigma.4.5.mm.3D_glszm_GrayLevelNonUniformity, wavelet.LLL_gldm_GrayLevelNonUniformity, log.sigma.0.5.mm.3D_glrlm_GrayLevelNonUniformityNormalized, wavelet.LHH_glrlm_GrayLevelNonUniformity, log.sigma.2.5.mm.3D_glszm_GrayLevelNonUniformity, wavelet.HLH_glrlm_GrayLevelNonUniformity, wavelet.HLL_glszm_GrayLevelNonUniformity, , wavelet.LHH_gldm_GrayLevelNonUniformity, wavelet.HLH_gldm_GrayLevelNonUniformity, wavelet.HHL_glrlm_GrayLevelNonUniformity, wavelet.HHH_gldm_GrayLevelNonUniformity)

$$GLN=\frac{\sum_{i=1}^{N_{g}} (\sum_{j=1}^{N_{s}} P(i,j))^{2}}{N_{z}}$$

GLN measures the variability of gray-level intensity values in the image, with a lower value indicating more homogeneity in intensity values.

1. **Energy** (exponential_firstorder_Energy, squareroot_firstorder_Energy, logarithm_firstorder_Energy, original_firstorder_Energy, log.sigma.2.5.mm.3D_firstorder_Energy, log.sigma.3.5.mm.3D_firstorder_Energy, log.sigma.1.5.mm.3D_firstorder_Energy,log.sigma.4.5.mm.3D_firstorder_Energy, log.sigma.0.5.mm.3D_firstorder_Energy, wavelet.LLL_firstorder_Energy, wavelet.LHL_firstorder_Energy, square_firstorder_Energy)

$$\text{e}nergy=\sum_{i=1}^{N_{p}} (X(i)+c)^{2}$$

Here, c is optional value, defined by **voxelArrayShift**, which shifts the intensities to prevent negative values in X. This ensures that voxels with the lowest gray values contribute the least to Energy, instead of voxels with gray level intensity closest to 0.

Energy is a measure of the magnitude of voxel values in an image. A larger values implies a greater sum of the squares of these values.

1. **Total Energy**（exponential_firstorder_TotalEnergy, squareroot_firstorder_TotalEnergy, logarithm_firstorder_TotalEnergy, original_firstorder_TotalEnergy, log.sigma.2.5.mm.3D_firstorder_TotalEnergy, log.sigma.3.5.mm.3D_firstorder_TotalEnergy, log.sigma.1.5.mm.3D_firstorder_TotalEnergy, log.sigma.4.5.mm.3D_firstorder_TotalEnergy, log.sigma.0.5.mm.3D_firstorder_TotalEnergy, wavelet.LLL_firstorder_TotalEnergy, wavelet.LHL_firstorder_TotalEnergy, square_firstorder_TotalEnergy)

$$total\begin{matrix} \\ \end{matrix}energy=V_{voxel}\sum_{i=1}^{N_{p}} (X(i)+c)^{2}$$

Here, c is optional value, defined by **voxelArrayShift**, which shifts the intensities to prevent negative values in X. This ensures that voxels with the lowest gray values contribute the least to Energy, instead of voxels with gray level intensity closest to 0.

Total Energy is the value of Energy feature scaled by the volume of the voxel in cubic mm.

1. **Zone Variance (ZV)** (log.sigma.4.5.mm.3D_glszm_ZoneVariance, original_glszm_ZoneVariance, log.sigma.3.5.mm.3D_glszm_ZoneVariance, log.sigma.0.5.mm.3D_glszm_ZoneVariance, log.sigma.1.5.mm.3D_glszm_ZoneVariance, log.sigma.2.5.mm.3D_glszm_ZoneVariance, squareroot_glszm_ZoneVariance, wavelet.LLH_glszm_ZoneVariance, square_glszm_ZoneVariance, logarithm_glszm_ZoneVariance, wavelet.LHL_glszm_ZoneVariance, wavelet.LLL_glszm_ZoneVariance)

$$ZV=\sum_{i=1}^{N_{g}} \sum_{j=1}^{N_{s}} p(i,j)(j-u)^{2}$$

Here, $u=\sum_{i=1}^{N_{g}} \sum_{j=1}^{N_{s}} p(i,j)j$

ZV measures the variance in zone size volumes for the zones.

1. **SurfaceArea** (original_shape_SurfaceArea)

$$\begin{matrix} A_{i}=\frac{1}{2}|a_{i}b_{i}\times a_{i}c_{i}|(1) \\ \end{matrix}$$

$$A=\sum_{i=1}^{N_{f}} A_{i}(2)$$

where:

 $a_{i}b_{i}$ and $a_{i}c_{i}$ are edges of the $i^{th}$ triangle in the mesh, formed by vertices $a_{i}$, $b_{i}$ and $c_{i}$.

To calculate the surface area, first the surface area $A_{i}$ of each triangle in the mesh is calculated (1). The total surface area is then obtained by taking the sum of all calculated sub-areas (2).

1. **Dependence Non-Uniformity (DN)** (original_gldm_DependenceNonUniformity, log.sigma.0.5.mm.3D_gldm_DependenceNonUniformity, wavelet.LHH_gldm_DependenceNonUniformity, squareroot_gldm_DependenceNonUniformity, log.sigma.1.5.mm.3D_gldm_DependenceNonUniformity, wavelet.HLH_gldm_DependenceNonUniformity, wavelet.LLH_gldm_DependenceNonUniformity, log.sigma.2.5.mm.3D_gldm_DependenceNonUniformity, wavelet.LHL_gldm_DependenceNonUniformity, wavelet.HHH_gldm_DependenceNonUniformity, logarithm_gldm_DependenceNonUniformity, wavelet.LLL_gldm_DependenceNonUniformity, exponential_gldm_DependenceNonUniformity, square_gldm_DependenceNonUniformity, wavelet.HHL_gldm_DependenceNonUniformity, log.sigma.3.5.mm.3D_gldm_DependenceNonUniformity, log.sigma.4.5.mm.3D_gldm_DependenceNonUniformity, wavelet.HLL_gldm_DependenceNonUniformity)

$$DN=\frac{\sum_{j=1}^{N_{d}} (\sum_{i=1}^{N_{g}} P(i,j))^{2}}{N_{z}}$$

Measures the similarity of dependence throughout the image, with a lower value indicating more homogeneity among dependencies in the image.

1. **Inverse Difference Moment (IDM)** (log.sigma.4.5.mm.3D_glcm_Idm, log.sigma.3.5.mm.3D_glcm_Idm, log.sigma.2.5.mm.3D_glcm_Idm, log.sigma.1.5.mm.3D_glcm_Idm, log.sigma.0.5.mm.3D_glcm_Idm, original_glcm_Idm, wavelet.LLH_glcm_Idm)

$$IDM=\sum_{k=0}^{N_{g}-1} \frac{p_{x-y}(k)}{1+k^{2}}$$

IDM (a.k.a Homogeneity 2) is a measure of the local homogeneity of an image. IDM weights are the inverse of the Contrast weights (decreasing exponentially from the diagonal i=j in the GLCM).

1. **Maximum 3D Diameter** (original_shape_Maximum3DDiameter)

Maximum 3D diameter is defined as the largest pairwise Euclidean distance between tumor surface mesh vertices.

Also known as Feret Diameter.

1. **Run Percentage (RP)** ( log.sigma.4.5.mm.3D_glrlm_RunPercentage, log.sigma.3.5.mm.3D_glrlm_RunPercentage, log.sigma.2.5.mm.3D_glrlm_RunPercentage, log.sigma.1.5.mm.3D_glrlm_RunPercentage, log.sigma.0.5.mm.3D_glrlm_RunPercentage, square_glrlm_RunPercentage, original_glrlm_RunPercentage, exponential_glrlm_RunPercentage, wavelet.LLH_glrlm_RunPercentage)

$$RP=\frac{N_{r}(\theta)}{N_{p}}$$

RP measures the coarseness of the texture by taking the ratio of number of runs and number of voxels in the ROI.

Values are in range $\frac{1}{N_{p}}\leq RP\leq1$, with higher values indicating a larger portion of the ROI consists of short runs (indicates a more fine texture).

1. **Contrast** (log.sigma.4.5.mm.3D_ngtdm_Contrast, log.sigma.3.5.mm.3D_ngtdm_Contrast, log.sigma.1.5.mm.3D_ngtdm_Contrast, log.sigma.2.5.mm.3D_ngtdm_Contrast, log.sigma.0.5.mm.3D_ngtdm_Contrast, original_ngtdm_Contrast, log.sigma.4.5.mm.3D_glcm_Contrast, log.sigma.3.5.mm.3D_glcm_Contrast, log.sigma.2.5.mm.3D_glcm_Contrast, wavelet.LLH_ngtdm_Contrast, squareroot_ngtdm_Contrast, wavelet.LHL_ngtdm_Contrast, wavelet.LLL_ngtdm_Contrast, log.sigma.1.5.mm.3D_glcm_Contrast, log.sigma.0.5.mm.3D_glcm_Contrast, logarithm_ngtdm_Contrast, wavelet.LHH_ngtdm_Contrast, square_ngtdm_Contrast, wavelet.HLH_ngtdm_Contrast, wavelet.HHL_ngtdm_Contrast)

$$contrast=\sum_{i=1}^{N_{g}} \sum_{j=1}^{N_{g}} (i-j)^{2}p(i,j)$$

Contrast is a measure of the local intensity variation, favoring values away from the diagonal $(i=j)$. A larger value correlates with a greater disparity in intensity values among neighboring voxels.

1. **LargeDependenceEmphasis (LDE)** (log.sigma.4.5.mm.3D_gldm_LargeDependenceEmphasis, log.sigma.3.5.mm.3D_gldm_LargeDependenceEmphasis, log.sigma.1.5.mm.3D_gldm_LargeDependenceEmphasis, log.sigma.2.5.mm.3D_gldm_LargeDependenceEmphasis, log.sigma.0.5.mm.3D_gldm_LargeDependenceEmphasis, original_gldm_LargeDependenceEmphasis, square_gldm_LargeDependenceEmphasis, wavelet.LLH_gldm_LargeDependenceEmphasis, exponential_gldm_LargeDependenceEmphasis)

$$LDE=\frac{\sum_{i=1}^{N_{g}} \sum_{j=1}^{N_{d}} P(i,j)j^{2}}{N_{z}}$$

A measure of the distribution of large dependencies, with a greater value indicative of larger dependence and more homogeneous textures.

1. **90th percentile** ( original_firstorder_90Percentile, squareroot_firstorder_90Percentile, logarithm_firstorder_90Percentile, exponential_firstorder_90Percentile, log.sigma.1.5.mm.3D_firstorder_90Percentile, square_firstorder_90Percentile, log.sigma.2.5.mm.3D_firstorder_90Percentile)

The $90^{th}$ percentile of X.

1. **Run Variance  (RV)** (log.sigma.3.5.mm.3D_glrlm_RunVariance, log.sigma.4.5.mm.3D_glrlm_RunVariance, log.sigma.1.5.mm.3D_glrlm_RunVariance, square_glrlm_RunVariance, log.sigma.2.5.mm.3D_glrlm_RunVariance, exponential_glrlm_RunVariance, log.sigma.0.5.mm.3D_glrlm_RunVariance, original_glrlm_RunVariance, wavelet.LLH_glrlm_RunVariance)

$$RV=\sum_{i=1}^{N_{g}} \sum_{j=1}^{N_{r}} p(i,j|\theta)(j-u)^{2}$$

Here, $u=\sum_{i=1}^{N_{g}} \sum_{j=1}^{N_{r}} p(i,j|\theta)j$

RV is a measure of the variance in runs for the run lengths.

1. Complexity (log.sigma.4.5.mm.3D_ngtdm_Complexity, log.sigma.3.5.mm.3D_ngtdm_Complexity, log.sigma.2.5.mm.3D_ngtdm_Complexity, logarithm_ngtdm_Complexity, log.sigma.0.5.mm.3D_ngtdm_Complexity, squareroot_ngtdm_Complexity, wavelet.LLL_ngtdm_Complexity, log.sigma.1.5.mm.3D_ngtdm_Complexity, original_ngtdm_Complexity, wavelet.LLH_ngtdm_Complexity, wavelet.LHL_ngtdm_Complexity)

$Complexity=\frac{1}{N_{v,p}}\sum_{i=1}^{N_{g}} \sum_{j=1}^{N_{g}} |i-j|\frac{p_{i}s_{i}+p_{j}s_{j}}{p_{i}+p_{j}}$ ,where $p_{i}\neq0$, $p_{j}\neq0$

An image is considered complex when there are many primitive components in the image, i.e. the image is non-uniform and there are many rapid changes in gray level intensity.

1. **Large Area Low Gray Level Emphasis (LALGLE)** (logarithm_glszm_LargeAreaLowGrayLevelEmphasis, log.sigma.4.5.mm.3D_glszm_LargeAreaLowGrayLevelEmphasis, wavelet.LLH_glszm_LargeAreaLowGrayLevelEmphasis, log.sigma.0.5.mm.3D_glszm_LargeAreaLowGrayLevelEmphasis, log.sigma.1.5.mm.3D_glszm_LargeAreaLowGrayLevelEmphasis, square_glszm_LargeAreaLowGrayLevelEmphasis, log.sigma.2.5.mm.3D_glszm_LargeAreaLowGrayLevelEmphasis, log.sigma.3.5.mm.3D_glszm_LargeAreaLowGrayLevelEmphasis, exponential_glszm_LargeAreaLowGrayLevelEmphasis, original_glszm_LargeAreaLowGrayLevelEmphasis, squareroot_glszm_LargeAreaLowGrayLevelEmphasis,

$$LALGLE=\frac{\sum_{i=1}^{N_{g}} \sum_{j=1}^{N_{s}} \frac{P(i,j)j^{2}}{i^{2}}}{N_{z}}$$

LALGLE measures the proportion in the image of the joint distribution of larger size zones with lower gray-level values.

1. **Run Length Non-Uniformity Normalized (RLNN)** (log.sigma.4.5.mm.3D_glrlm_RunLengthNonUniformityNormalized, log.sigma.3.5.mm.3D_glrlm_RunLengthNonUniformityNormalized, log.sigma.2.5.mm.3D_glrlm_RunLengthNonUniformityNormalized, log.sigma.1.5.mm.3D_glrlm_RunLengthNonUniformityNormalized, log.sigma.0.5.mm.3D_glrlm_RunLengthNonUniformityNormalized, original_glrlm_RunLengthNonUniformityNormalized, square_glrlm_RunLengthNonUniformityNormalized, exponential_glrlm_RunLengthNonUniformityNormalized, wavelet.LLH_glrlm_RunLengthNonUniformityNormalized)

$$RLNN=\frac{\sum_{j=1}^{N_{r}} (\sum_{i=1}^{N_{g}} P(i,j|\theta))^{2}}{N_{r}(\theta)^{2}}$$

RLNN measures the similarity of run lengths throughout the image, with a lower value indicating more homogeneity among run lengths in the image. This is the normalized version of the RLN formula.

1. **Short Run Emphasis  (SRE)** (log.sigma.4.5.mm.3D_glrlm_ShortRunEmphasis, log.sigma.3.5.mm.3D_glrlm_ShortRunEmphasis, log.sigma.2.5.mm.3D_glrlm_ShortRunEmphasis, log.sigma.1.5.mm.3D_glrlm_ShortRunEmphasis, log.sigma.0.5.mm.3D_glrlm_ShortRunEmphasis, original_glrlm_ShortRunEmphasis, wavelet.LLH_glrlm_ShortRunEmphasis)

$$SRE=\frac{\sum_{i=1}^{N_{g}} \sum_{j=1}^{N_{r}} \frac{P(i,j|\theta)}{j^{2}}}{N_{r}(\theta)}$$

SRE is a measure of the distribution of short run lengths, with a greater value indicative of shorter run lengths and more fine textural textures.

1. **Long Run Emphasis  (LRE)** (log.sigma.4.5.mm.3D_glrlm_LongRunEmphasis, log.sigma.3.5.mm.3D_glrlm_LongRunEmphasis, log.sigma.1.5.mm.3D_glrlm_LongRunEmphasis, log.sigma.2.5.mm.3D_glrlm_LongRunEmphasis, square_glrlm_LongRunEmphasis, log.sigma.0.5.mm.3D_glrlm_LongRunEmphasis, original_glrlm_LongRunEmphasis, exponential_glrlm_LongRunEmphasis, wavelet.LLH_glrlm_LongRunEmphasis)

$$LRE=\frac{\sum_{i=1}^{N_{g}} \sum_{j=1}^{N_{r}} P(i,j|\theta)j^{2}}{N_{r}(\theta)}$$

LRE is a measure of the distribution of long run lengths, with a greater value indicative of longer run lengths and more coarse structural textures.

1. **Run Length Non-Uniformity (RLN)** (wavelet.LLL_glrlm_RunLengthNonUniformity, wavelet.HLH_glrlm_RunLengthNonUniformity, logarithm_glrlm_RunLengthNonUniformity, wavelet.LHL_glrlm_RunLengthNonUniformity, wavelet.HLL_glrlm_RunLengthNonUniformity, wavelet.HHH_glrlm_RunLengthNonUniformity, wavelet.LHH_glrlm_RunLengthNonUniformity, wavelet.HHL_glrlm_RunLengthNonUniformity, log.sigma.0.5.mm.3D_glrlm_RunLengthNonUniformity, squareroot_glrlm_RunLengthNonUniformity, log.sigma.1.5.mm.3D_glrlm_RunLengthNonUniformity, original_glrlm_RunLengthNonUniformity, log.sigma.2.5.mm.3D_glrlm_RunLengthNonUniformity, wavelet.LLH_glrlm_RunLengthNonUniformity, log.sigma.3.5.mm.3D_glrlm_RunLengthNonUniformity, exponential_glrlm_RunLengthNonUniformity, log.sigma.4.5.mm.3D_glrlm_RunLengthNonUniformity, square_glrlm_RunLengthNonUniformity)

$$RLN=\frac{\sum_{j=1}^{N_{r}} (\sum_{i=1}^{N_{g}} P(i,j|\theta))^{2}}{N_{r}(\theta)}$$

RLN measures the similarity of run lengths throughout the image, with a lower value indicating more homogeneity among run lengths in the image.

1. **Large Dependence High Gray Level Emphasis (LDHGLE)**（log.sigma.3.5.mm.3D_gldm_LargeDependenceHighGrayLevelEmphasis, log.sigma.2.5.mm.3D_gldm_LargeDependenceHighGrayLevelEmphasis, log.sigma.1.5.mm.3D_gldm_LargeDependenceHighGrayLevelEmphasis, log.sigma.4.5.mm.3D_gldm_LargeDependenceHighGrayLevelEmphasis, logarithm_gldm_LargeDependenceHighGrayLevelEmphasis, square_gldm_LargeDependenceHighGrayLevelEmphasis, exponential_gldm_LargeDependenceHighGrayLevelEmphasis, wavelet.HLL_gldm_LargeDependenceHighGrayLevelEmphasis, log.sigma.0.5.mm.3D_gldm_LargeDependenceHighGrayLevelEmphasis)

$$LDHGLE=\frac{\sum_{i=1}^{N_{g}} \sum_{j=1}^{N_{d}} P(i,j)i^{2}j^{2}}{N_{z}}$$

Measures the joint distribution of large dependence with higher gray-level values.

1. **Root Mean Squared (RMS)** (log.sigma.1.5.mm.3D_firstorder_RootMeanSquared, log.sigma.0.5.mm.3D_firstorder_RootMeanSquared, log.sigma.2.5.mm.3D_firstorder_RootMeanSquared, squareroot_firstorder_RootMeanSquared, original_firstorder_RootMeanSquared, logarithm_firstorder_RootMeanSquared, exponential_firstorder_RootMeanSquared, log.sigma.3.5.mm.3D_firstorder_RootMeanSquared)

$$RMS=\sqrt{\frac{1}{N_{p}}\sum_{i=1}^{N_{p}} (X(i)+c)^{2}}$$

Here, c is optional value, defined by **voxelArrayShift**, which shifts the intensities to prevent negative values in X. This ensures that voxels with the lowest gray values contribute the least to RMS, instead of voxels with gray level intensity closest to 0.

RMS is the square-root of the mean of all the squared intensity values. It is another measure of the magnitude of the image values. This feature is volume-confounded, a larger value of cc increases the effect of volume-confounding.

1. **Difference Average** (log.sigma.4.5.mm.3D_glcm_DifferenceAverage, log.sigma.3.5.mm.3D_glcm_DifferenceAverage, log.sigma.2.5.mm.3D_glcm_DifferenceAverage, log.sigma.1.5.mm.3D_glcm_DifferenceAverage, log.sigma.0.5.mm.3D_glcm_DifferenceAverage, original_glcm_DifferenceAverage, wavelet.LLH_glcm_DifferenceAverage)

$$differece\begin{matrix} \\ \end{matrix}average=\sum_{k=0}^{N_{g}-1} kp_{x-y}(k)$$

Difference Average measures the relationship between occurrences of pairs with similar intensity values and occurrences of pairs with differing intensity values.

1. **Maximum 2D diameter (Row) (original_shape_Maximum2DDiameterRow)**

Maximum 2D diameter (Row) is defined as the largest pairwise Euclidean distance between tumor surface mesh vertices in the column-slice (usually the sagittal) plane.

1. **Maximum 2D diameter (Slice) (original_shape_Maximum2DDiameterSlice)**

Maximum 2D diameter (Slice) is defined as the largest pairwise Euclidean distance between tumor surface mesh vertices in the row-column (generally the axial) plane.

1. **Maximum 2D diameter (Column) (original_shape_Maximum2DDiameterColumn)**

Maximum 2D diameter (Column) is defined as the largest pairwise Euclidean distance between tumor surface mesh vertices in the row-slice (usually the coronal) plane.

1. **Coarseness** (log.sigma.4.5.mm.3D_ngtdm_Coarseness, original_ngtdm_Coarseness, wavelet.LLH_ngtdm_Coarseness, log.sigma.0.5.mm.3D_ngtdm_Coarseness, log.sigma.3.5.mm.3D_ngtdm_Coarseness, wavelet.LHL_ngtdm_Coarseness, wavelet.LHH_ngtdm_Coarseness, log.sigma.1.5.mm.3D_ngtdm_Coarseness, squareroot_ngtdm_Coarseness, log.sigma.2.5.mm.3D_ngtdm_Coarseness, wavelet.HLH_ngtdm_Coarseness, wavelet.LLL_ngtdm_Coarseness, wavelet.HHH_ngtdm_Coarseness, wavelet.HHL_ngtdm_Coarseness, logarithm_ngtdm_Coarseness, wavelet.HLL_ngtdm_Coarseness)

$$Coarseness=\frac{1}{\sum_{i=1}^{N_{g}} p_{i}s_{i}}$$

Coarseness is a measure of average difference between the center voxel and its neighbourhood and is an indication of the spatial rate of change. A higher value indicates a lower spatial change rate and a locally more uniform texture.

1. B. $\sum_{i=1}^{N_{g}} p_{i}s_{i}$ potentially evaluates to 0 (in case of a completely homogeneous image). If this is the case, an arbitrary value of $10^{6}$ is returned.
2. **Busyness** (log.sigma.4.5.mm.3D_ngtdm_Busyness, log.sigma.0.5.mm.3D_ngtdm_Busyness, squareroot_ngtdm_Busyness, log.sigma.2.5.mm.3D_ngtdm_Busyness, logarithm_ngtdm_Busyness, log.sigma.3.5.mm.3D_ngtdm_Busyness, original_ngtdm_Busyness, wavelet.LLH_ngtdm_Busyness, log.sigma.1.5.mm.3D_ngtdm_Busyness, wavelet.LLL_ngtdm_Busyness)

$Busyness=\frac{\sum_{i=1}^{N_{g}} p_{i}s_{i}}{\sum_{i=1}^{N_{g}} \sum_{j=1}^{N_{g}} |ip_{i}-jp_{j}|}$ , where $p_{i}\neq0,p_{j}\neq0$

A measure of the change from a pixel to its neighbour. A high value for busyness indicates a ‘busy’ image, with rapid changes of intensity between pixels and its neighbourhood.

1. B. if $N_{g,p}=1$, then $busyness=\frac{0}{0}$. If this is the case, 0 is returned, as it concerns a fully homogeneous region.
2. **Dependence Non-Uniformity Normalized  (DNN)** (log.sigma.4.5.mm.3D_gldm_DependenceNonUniformityNormalized, log.sigma.2.5.mm.3D_gldm_DependenceNonUniformityNormalized, log.sigma.3.5.mm.3D_gldm_DependenceNonUniformityNormalized, log.sigma.1.5.mm.3D_gldm_DependenceNonUniformityNormalized, original_gldm_DependenceNonUniformityNormalized, log.sigma.0.5.mm.3D_gldm_DependenceNonUniformityNormalized, exponential_gldm_DependenceNonUniformityNormalized, square_gldm_DependenceNonUniformityNormalized, wavelet.LLH_gldm_DependenceNonUniformityNormalized)

$$DNN=\frac{\sum_{j=1}^{N_{d}} (\sum_{i=1}^{N_{g}} P(i,j))^{2}}{N_{z}^{2}}$$

Measures the similarity of dependence throughout the image, with a lower value indicating more homogeneity among dependencies in the image. This is the normalized version of the DLN formula.

1. **Small Dependence Emphasis (SDE)** (log.sigma.3.5.mm.3D_gldm_SmallDependenceEmphasis, log.sigma.4.5.mm.3D_gldm_SmallDependenceEmphasis, log.sigma.2.5.mm.3D_gldm_SmallDependenceEmphasis, log.sigma.1.5.mm.3D_gldm_SmallDependenceEmphasis, wavelet.LLH_gldm_SmallDependenceEmphasis, log.sigma.0.5.mm.3D_gldm_SmallDependenceEmphasis, original_gldm_SmallDependenceEmphasis, squareroot_gldm_SmallDependenceEmphasis, wavelet.LLL_gldm_SmallDependenceEmphasis, square_gldm_SmallDependenceEmphasis)

$$SDE=\frac{\sum_{i=1}^{N_{g}} \sum_{j=1}^{N_{d}} \frac{P(i,j)}{i^{2}}}{N_{z}}$$

A measure of the distribution of small dependencies, with a greater value indicative of smaller dependence and less homogeneous textures.

1. Median (log.sigma.1.5.mm.3D_firstorder_Median, wavelet.LLH_firstorder_Median, log.sigma.0.5.mm.3D_firstorder_Median, logarithm_firstorder_Median, squareroot_firstorder_Median, square_firstorder_Median, log.sigma.2.5.mm.3D_firstorder_Median, original_firstorder_Median, wavelet.LHL_firstorder_Median, log.sigma.3.5.mm.3D_firstorder_Median, exponential_firstorder_Median)

The median gray level intensity within the ROI.

1. **10 Percentile** ( log.sigma.1.5.mm.3D_firstorder_10Percentile, log.sigma.0.5.mm.3D_firstorder_10Percentile, log.sigma.2.5.mm.3D_firstorder_10Percentile, log.sigma.3.5.mm.3D_firstorder_10Percentile, wavelet.LLH_firstorder_10Percentile, log.sigma.4.5.mm.3D_firstorder_10Percentile, square_firstorder_10Percentile)

The $10^{\text{th}}$ percentile of X.

1. **Strength** (log.sigma.4.5.mm.3D_ngtdm_Strength, squareroot_ngtdm_Strength, log.sigma.3.5.mm.3D_ngtdm_Strength, log.sigma.2.5.mm.3D_ngtdm_Strength, log.sigma.0.5.mm.3D_ngtdm_Strength, original_ngtdm_Strength, logarithm_ngtdm_Strength, wavelet.LLL_ngtdm_Strength, log.sigma.1.5.mm.3D_ngtdm_Strength)

$$Strength=\frac{\sum_{i=1}^{N_{g}} \sum_{j=1}^{N_{g}} (p_{i}+p_{j})(i-j)^{2}}{\sum_{i=1}^{N_{g}} s_{i}},where\begin{matrix} \\ \end{matrix}p_{i}\neq0,p_{j}\neq0$$

Strength is a measure of the primitives in an image. Its value is high when the primitives are easily defined and visible, i.e. an image with slow change in intensity but more large coarse differences in gray level intensities.

N.B. $\sum_{i=1}^{N_{g}} s_{i}$ potentially evaluates to 0 (in case of a completely homogeneous image). If this is the case, 0 is returned.

38. **Maximum Probability** (log.sigma.1.5.mm.3D_glcm_MaximumProbability, log.sigma.2.5.mm.3D_glcm_MaximumProbability, log.sigma.3.5.mm.3D_glcm_MaximumProbability, log.sigma.4.5.mm.3D_glcm_MaximumProbability, log.sigma.0.5.mm.3D_glcm_MaximumProbability)

$$\max imum\begin{matrix} \\ \end{matrix}probability=\max(p(i,j))$$

Maximum Probability is occurrences of the most predominant pair of neighboring intensity values.

1. **Zone Percentage (ZP)** (log.sigma.3.5.mm.3D_glszm_ZonePercentage, log.sigma.4.5.mm.3D_glszm_ZonePercentage, log.sigma.2.5.mm.3D_glszm_ZonePercentage, wavelet.LLH_glszm_ZonePercentage, log.sigma.1.5.mm.3D_glszm_ZonePercentage, log.sigma.0.5.mm.3D_glszm_ZonePercentage, original_glszm_ZonePercentage, squareroot_glszm_ZonePercentage, wavelet.LLL_glszm_ZonePercentage, square_glszm_ZonePercentage)

$$ZP=\frac{N_{z}}{N_{p}}$$

ZP measures the coarseness of the texture by taking the ratio of number of zones and number of voxels in the ROI.

Values are in range $\frac{1}{N_{p}}\leq ZP\leq1$, with higher values indicating a larger portion of the ROI consists of small zones (indicates a more fine texture).

1. **Mean** (log.sigma.1.5.mm.3D_firstorder_Mean, log.sigma.0.5.mm.3D_firstorder_Mean, square_firstorder_Mean, log.sigma.2.5.mm.3D_firstorder_Mean, logarithm_firstorder_Mean, wavelet.LHL_firstorder_Mean)

$$mean=\frac{1}{N_{p}}\sum_{i=1}^{N_{p}} X(i)$$

The average gray level intensity within the ROI.

1. **Dependence Variance  (DV)** (log.sigma.4.5.mm.3D_gldm_DependenceVariance, log.sigma.1.5.mm.3D_gldm_DependenceVariance, log.sigma.3.5.mm.3D_gldm_DependenceVariance, log.sigma.2.5.mm.3D_gldm_DependenceVariance, original_gldm_DependenceVariance, exponential_gldm_DependenceVariance, log.sigma.0.5.mm.3D_gldm_DependenceVariance, square_gldm_DependenceVariance)

$$DV=\sum_{i=1}^{N_{g}} \sum_{j=1}^{N_{d}} p(i,j)(j-u)^{2},where\begin{matrix} \\ \end{matrix}u=\sum_{i=1}^{N_{g}} \sum_{j=1}^{N_{d}} jp(i,j)$$

Measures the variance in dependence size in the image.

1. **Skewness** ( log.sigma.2.5.mm.3D_firstorder_Skewness, log.sigma.1.5.mm.3D_firstorder_Skewness, log.sigma.0.5.mm.3D_firstorder_Skewness,

$$skewness=\frac{u_{3}}{\sigma^{3}}=\frac{\frac{1}{N_{p}}\sum_{i=1}^{N_{p}} (X(i)-\overline{X})^{3}}{(\sqrt{\frac{1}{N_{p}}\sum_{i=1}^{N_{p}} (X(i)-\overline{X})^{2})^{3}}}$$

Where $u_{3}$ is the $3^{rd}$ central moment.

Skewness measures the asymmetry of the distribution of values about the Mean value. Depending on where the tail is elongated and the mass of the distribution is concentrated, this value can be positive or negative.

Related links:

<https://en.wikipedia.org/wiki/Skewness>

1. **Inverse Difference Normalized (IDN)** (log.sigma.1.5.mm.3D_glcm_Idn, log.sigma.4.5.mm.3D_glcm_Idn, log.sigma.3.5.mm.3D_glcm_Idn, log.sigma.2.5.mm.3D_glcm_Idn, log.sigma.0.5.mm.3D_glcm_Idn, squareroot_glcm_Idn, wavelet.LLL_glcm_Idn)

$$IDN=\sum_{k=0}^{N_{g}-1} \frac{p_{x-y}(k)}{1+\text{（}\frac{k}{N_{g}})}$$

IDN (inverse difference normalized) is another measure of the local homogeneity of an image. Unlike Homogeneity1, IDN normalizes the difference between the neighboring intensity values by dividing over the total number of discrete intensity values.

1. **Minor Axis** (original_shape_MinorAxis)

$\min or\begin{matrix} \\ \end{matrix}axis=4\sqrt{\lambda_{\min or}}$

This feature yield the second-largest axis length of the ROI-enclosing ellipsoid and is calculated using the largest principal component $\lambda_{\min or}$.

The principal component analysis is performed using the physical coordinates of the voxel centers defining the ROI. It therefore takes spacing into account, but does not make use of the shape mesh.

1. **Difference Entropy** ( log.sigma.3.5.mm.3D_glcm_DifferenceEntropy, log.sigma.2.5.mm.3D_glcm_DifferenceEntropy, log.sigma.1.5.mm.3D_glcm_DifferenceEntropy, log.sigma.0.5.mm.3D_glcm_DifferenceEntropy, log.sigma.4.5.mm.3D_glcm_DifferenceEntropy, original_glcm_DifferenceEntropy, wavelet.LLH_glcm_DifferenceEntropy)

$$difference\begin{matrix} \\ \end{matrix}entropy=\sum_{k=0}^{N_{g}-1} p_{x-y}(k)\log_{2}(p_{x-y}(k)+\varepsilon)$$

Difference Entropy is a measure of the randomness/variability in neighborhood intensity value differences.

1. **Informational Measure of Correlation (IMC) 1** (log.sigma.2.5.mm.3D_glcm_Imc1, log.sigma.1.5.mm.3D_glcm_Imc1, logarithm_glcm_Imc1, log.sigma.3.5.mm.3D_glcm_Imc1, log.sigma.4.5.mm.3D_glcm_Imc1, squareroot_glcm_Imc1, squareroot_glcm_Imc1)

$$IMC\begin{matrix} \\ \end{matrix}1=\frac{HXY-HXY1}{\max\{HX,HY\}}$$

IMC1 assesses the correlation between the probability distributions of ii and jj (quantifying the complexity of the texture), using mutual information I(x, y):

$$\begin{matrix} I(i,j)=\sum_{i=1}^{N_{g}} \sum_{j=1}^{N_{g}} p(i,j)\log_{2}\text{（}\frac{p(i,j)}{p_{x}(i)p_{y}(y)}) \\ =\sum_{i=1}^{N_{g}} \sum_{j=1}^{N_{g}} p(i,j)(\log_{2}(p(i,j))-\log_{2}(p_{x}(i)p_{y}(j))) \\ =\sum_{i=1}^{N_{g}} \sum_{j=1}^{N_{g}} p(i,j)\log_{2}(p(i,j))-\sum_{i=1}^{N_{g}} \sum_{j=1}^{N_{g}} p(i,j)\log_{2}(p_{x}(i)p_{y}(j)) \\ =-HXY+HXY1 \end{matrix}$$

However, in this formula, the numerator is defined as $HXY-HXY1$ (i.e. $-I(x,y)$), and is therefore $\leq0$. This reflects how this feature is defined in the original Haralick paper.

In the case where the distributions are independent, there is no mutual information and the result will therefore be 0. In the case of uniform distribution with complete dependence, mutual information will be equal to $\log_{2}(N_{g})$.

Finally, $HXY-HXY1$ is divided by the maximum of the 2 marginal entropies, where in the latter case of complete dependence (not necessarily uniform; low complexity) it will result in $IMC1=-1$, as $HX=HY=I(i,j)$

1. **Cluster Shade** (log.sigma.2.5.mm.3D_glcm_ClusterShade)

$$cluster\begin{matrix} \\ \end{matrix}shade=\sum_{i=1}^{N_{g}} \sum_{j=1}^{N_{g}} (i+j-u_{x}-u_{y})^{3}p(i,j)$$

Cluster Shade is a measure of the skewness and uniformity of the GLCM. A higher cluster shade implies greater asymmetry about the mean.

1. **Joint Energy** (log.sigma.1.5.mm.3D_glcm_JointEnergy, log.sigma.2.5.mm.3D_glcm_JointEnergy, log.sigma.3.5.mm.3D_glcm_JointEnergy, log.sigma.4.5.mm.3D_glcm_JointEnergy, log.sigma.0.5.mm.3D_glcm_JointEnergy)

$$jo\mathrm{int}\begin{matrix} \\ \end{matrix}energy=\sum_{i=1}^{N_{g}} \sum_{j=1}^{N_{g}} (p(i,j))^{2}$$

Energy is a measure of homogeneous patterns in the image. A greater Energy implies that there are more instances of intensity value pairs in the image that neighbor each other at higher frequencies.

1. **Least Axis** (original_shape_LeastAxis)

$$least\begin{matrix} \\ \end{matrix}axis=4\sqrt{\lambda_{least}}$$

This feature yield the smallest axis length of the ROI-enclosing ellipsoid and is calculated using the largest principal component $\lambda_{least}$. In case of a 2D segmentation, this value will be 0.

The principal component analysis is performed using the physical coordinates of the voxel centers defining the ROI. It therefore takes spacing into account, but does not make use of the shape mesh.

1. **Difference Variance** (log.sigma.3.5.mm.3D_glcm_DifferenceVariance,log.sigma.0.5.mm.3D_glcm_DifferenceVariance, log.sigma.2.5.mm.3D_glcm_DifferenceVariance, log.sigma.1.5.mm.3D_glcm_DifferenceVariance, log.sigma.4.5.mm.3D_glcm_DifferenceVariance)

$$difference\begin{matrix} \\ \end{matrix}\mathrm{var}iance=\sum_{k=0}^{N_{g}-1} (k-DA)^{2}p_{x-y}(k)$$

Difference Variance is a measure of heterogeneity that places higher weights on differing intensity level pairs that deviate more from the mean.

1. **Long Run High Gray Level Emphasis  (LRHGLE)** (log.sigma.2.5.mm.3D_glrlm_LongRunHighGrayLevelEmphasis, log.sigma.3.5.mm.3D_glrlm_LongRunHighGrayLevelEmphasis, log.sigma.1.5.mm.3D_glrlm_LongRunHighGrayLevelEmphasis, exponential_glrlm_LongRunHighGrayLevelEmphasis, logarithm_glrlm_LongRunHighGrayLevelEmphasis, square_glrlm_LongRunHighGrayLevelEmphasis, log.sigma.4.5.mm.3D_glrlm_LongRunHighGrayLevelEmphasis)

$$LRHGLRE=\frac{\sum_{i=1}^{N_{g}} \sum_{j=1}^{N_{r}} P(i,j|\theta)i^{2}j^{2}}{N_{r}(\theta)}$$

LRHGLRE measures the joint distribution of long run lengths with higher gray-level values.

1. **Informational Measure of Correlation (IMC) 2** (log.sigma.2.5.mm.3D_glcm_Imc2, log.sigma.3.5.mm.3D_glcm_Imc2, log.sigma.0.5.mm.3D_glcm_Imc2, logarithm_glcm_Imc2)

$$IMC\begin{matrix} \\ \end{matrix}2=\sqrt{\text{1-}e^{-2(HXY2-HXY)}}$$

IMC2 also assesses the correlation between the probability distributions of  i and j (quantifying the complexity of the texture). Of interest is to note that $HXY1=HXY2$ and that $HXY2-HXY\geq0$ represents the mutual information of the 2 distributions. Therefore, the range of IMC2 = [0, 1), with 0 representing the case of 2 independent distributions (no mutual information) and the maximum value representing the case of 2 fully dependent and uniform distributions (maximal mutual information, equal to $\log_{2}(N_{g})$). In this latter case, the maximum value is then equal to $\sqrt{\text{1-}e^{-2\log_{2}(N_{g})}}$, approaching 1.

1. **Uniformity** (log.sigma.0.5.mm.3D_firstorder_Uniformity, log.sigma.1.5.mm.3D_firstorder_Uniformity, log.sigma.3.5.mm.3D_firstorder_Uniformity, wavelet.LLH_firstorder_Uniformity)

$$uniformity=\sum_{i=1}^{N_{g}} p(i)^{2}$$

Uniformity is a measure of the sum of the squares of each intensity value. This is a measure of the homogeneity of the image array, where a greater uniformity implies a greater homogeneity or a smaller range of discrete intensity values.

1. **Correlation** (log.sigma.2.5.mm.3D_glcm_Correlation, log.sigma.1.5.mm.3D_glcm_Correlation, log.sigma.3.5.mm.3D_glcm_Correlation)

$$correlation=\frac{\sum_{i=1}^{N_{g}} \sum_{j=1}^{N_{g}} p(i,j)ij-u_{x}u_{y}}{\sigma_{x}(i)\sigma_{y}(j)}$$

Correlation is a value between 0 (uncorrelated) and 1 (perfectly correlated) showing the linear dependency of gray level values to their respective voxels in the GLCM.

1. **Run Entropy (RE)** (log.sigma.2.5.mm.3D_glrlm_RunEntropy, exponential_glrlm_RunEntropy, log.sigma.1.5.mm.3D_glrlm_RunEntropy, square_glrlm_RunEntropy)

$$RE=-\sum_{i=1}^{N_{g}} \sum_{j=1}^{N_{r}} p(i,j|\theta)\log_{2}(p(i,j|\theta)+\varepsilon)$$

Here, $\varepsilon$ is an arbitrarily small positive number $(\approx2.2\times10^{\text{-16}})$.

RE measures the uncertainty/randomness in the distribution of run lengths and gray levels. A higher value indicates more heterogeneity in the texture patterns.

1. **Inverse Variance** (log.sigma.0.5.mm.3D_glcm_InverseVariance, log.sigma.3.5.mm.3D_glcm_InverseVariance, log.sigma.2.5.mm.3D_glcm_InverseVariance, log.sigma.1.5.mm.3D_glcm_InverseVariance, wavelet.LLH_glcm_InverseVariance, original_glcm_InverseVariance, log.sigma.4.5.mm.3D_glcm_InverseVariance)

$$inverse\begin{matrix} \\ \end{matrix}\mathrm{var}iance=\sum_{k=1}^{N_{g}-1} \frac{p_{x-y}(k)}{k^{2}}$$

Note that $k=0$ is skipped, as this would result in a division by 0.

1. **Long Run Low Gray Level Emphasis (LRLGLE)** (square_glrlm_LongRunLowGrayLevelEmphasis, exponential_glrlm_LongRunLowGrayLevelEmphasis, logarithm_glrlm_LongRunLowGrayLevelEmphasis)

$$LRLGLRE=\frac{\sum_{i=1}^{N_{g}} \sum_{j=1}^{N_{r}} \frac{P(i,j|\theta)j^{2}}{i^{2}}}{N_{r}(\theta)}$$

LRLGLRE measures the joint distribution of long run lengths with lower gray-level values.

1. **Inverse Difference Moment Normalized (IDMN)** (log.sigma.1.5.mm.3D_glcm_Idmn, log.sigma.4.5.mm.3D_glcm_Idmn, log.sigma.2.5.mm.3D_glcm_Idmn, log.sigma.3.5.mm.3D_glcm_Idmn, log.sigma.0.5.mm.3D_glcm_Idmn, wavelet.LLL_glcm_Idmn, original_glcm_Idmn)

$$IDMN=\sum_{k=0}^{N_{g}-1} \frac{p_{x-y}(k)}{1+(\frac{k^{2}}{N_{g}^{2}})}$$

IDMN (inverse difference moment normalized) is a measure of the local homogeneity of an image. IDMN weights are the inverse of the Contrast weights (decreasing exponentially from the diagonal $i=j$in the GLCM). Unlike Homogeneity2, IDMN normalizes the square of the difference between neighboring intensity values by dividing over the square of the total number of discrete intensity values.

1. **Joint Entropy** (log.sigma.1.5.mm.3D_glcm_JointEntropy, log.sigma.3.5.mm.3D_glcm_JointEntropy, log.sigma.0.5.mm.3D_glcm_JointEntropy, log.sigma.2.5.mm.3D_glcm_JointEntropy, log.sigma.4.5.mm.3D_glcm_JointEntropy, wavelet.LLH_glcm_JointEntropy)

$$jo\mathrm{int}\begin{matrix} \\ \end{matrix}entropy=-\sum_{i=1}^{N_{g}} \sum_{j=1}^{N_{g}} p(i,j)\log_{2}(p(i,j)+\varepsilon$$

Joint entropy is a measure of the randomness/variability in neighborhood intensity values.

1. **Small Dependence High Gray Level Emphasis  (SDHGLE)**

(log.sigma.3.5.mm.3D_gldm_SmallDependenceHighGrayLevelEmphasis, log.sigma.4.5.mm.3D_gldm_SmallDependenceHighGrayLevelEmphasis, log.sigma.2.5.mm.3D_gldm_SmallDependenceHighGrayLevelEmphasis)

Measures the joint distribution of small dependence with higher gray-level values.

1. **Interquartile Range** (log.sigma.0.5.mm.3D_firstorder_InterquartileRange, square_firstorder_InterquartileRange, exponential_firstorder_InterquartileRange, log.sigma.3.5.mm.3D_firstorder_InterquartileRange, log.sigma.1.5.mm.3D_firstorder_InterquartileRange, wavelet.LLH_firstorder_InterquartileRange)

$$\mathrm{int}erquartile\begin{matrix} \\ \end{matrix}range=P_{75}-P_{25}$$

Here $P_{25}$ and $P_{75}$ are the $25^{th}$ and $75^{\text{th}}$ percentile of the image array, respectively.

1. **Small Dependence Low Gray Level Emphasis  (SDLGLE)** (log.sigma.3.5.mm.3D_gldm_SmallDependenceLowGrayLevelEmphasis, log.sigma.4.5.mm.3D_gldm_SmallDependenceLowGrayLevelEmphasis, log.sigma.2.5.mm.3D_gldm_SmallDependenceLowGrayLevelEmphasis, log.sigma.1.5.mm.3D_gldm_SmallDependenceLowGrayLevelEmphasis, wavelet.HLH_gldm_SmallDependenceLowGrayLevelEmphasis, wavelet.LLL_gldm_SmallDependenceLowGrayLevelEmphasis, logarithm_gldm_SmallDependenceLowGrayLevelEmphasis, log.sigma.0.5.mm.3D_gldm_SmallDependenceLowGrayLevelEmphasis, wavelet.HLL_gldm_SmallDependenceLowGrayLevelEmphasis, squareroot_gldm_SmallDependenceLowGrayLevelEmphasis, square_gldm_SmallDependenceLowGrayLevelEmphasis)

$$SDLGLE=\frac{\sum_{i=1}^{N_{g}} \sum_{j=1}^{N_{d}} \frac{P(i,j)}{i^{2}j^{2}}}{N_{z}}$$

Measures the joint distribution of small dependence with lower gray-level values.

1. **Robust Mean Absolute Deviation  (rMAD)**（log.sigma.0.5.mm.3D_firstorder_RobustMeanAbsoluteDeviation, square_firstorder_RobustMeanAbsoluteDeviation, exponential_firstorder_RobustMeanAbsoluteDeviation, log.sigma.3.5.mm.3D_firstorder_RobustMeanAbsoluteDeviation, wavelet.LLH_firstorder_RobustMeanAbsoluteDeviation, log.sigma.1.5.mm.3D_firstorder_RobustMeanAbsoluteDeviation, original_firstorder_RobustMeanAbsoluteDeviation)

$$rMAD=\frac{1}{N_{10-90}}\sum_{i=1}^{N{}_{10-90}} |X_{10-90}(i)-\overline{X}_{10-90}|$$

Robust Mean Absolute Deviation is the mean distance of all intensity values from the Mean Value calculated on the subset of image array with gray levels in between, or equal to the $10^{th}$ and $90^{th}$ percentile.

1. **Mean Absolute Deviation** **(MAD)** (log.sigma.0.5.mm.3D_firstorder_MeanAbsoluteDeviation, log.sigma.3.5.mm.3D_firstorder_MeanAbsoluteDeviation, exponential_firstorder_MeanAbsoluteDeviation, log.sigma.2.5.mm.3D_firstorder_MeanAbsoluteDeviation, square_firstorder_MeanAbsoluteDeviation, wavelet.LLH_firstorder_MeanAbsoluteDeviation)

$$MAD=\frac{1}{N_{p}}\sum_{i=1}^{N_{p}} |X(i)-\overline{X}|$$

Mean Absolute Deviation is the mean distance of all intensity values from the Mean Value of the image array.

1. **Flatness** (original_shape_Flatness)

Flatness shows the relationship between the largest and smallest principal components in the ROI shape. For computational reasons, this feature is defined as the inverse of true flatness.

$$flatness=\sqrt{\frac{\lambda_{least}}{\lambda_{major}}}$$

Here, $\lambda_{major}$ and $\lambda_{least}$ are the lengths of the largest and smallest principal component axes. The values range between 1 (non-flat, sphere-like) and 0 (a flat object, or single-slice segmentation).

The principal component analysis is performed using the physical coordinates of the voxel centers defining the ROI. It therefore takes spacing into account, but does not make use of the shape mesh.

1. **Entropy** (log.sigma.0.5.mm.3D_firstorder_Entropy, log.sigma.3.5.mm.3D_firstorder_Entropy, wavelet.LLH_firstorder_Entropy, log.sigma.2.5.mm.3D_firstorder_Entropy)

$$entropy=-\sum_{i=1}^{N_{g}} p(i)\log_{2}(p(i)+\varepsilon)$$

Here, $\varepsilon$ is an arbitrarily small positive number ($\approx2.2\times10^{\text{-16}}$).

Entropy specifies the uncertainty/randomness in the image values. It measures the average amount of information required to encode the image values.

1. **Sum Entropy** (log.sigma.0.5.mm.3D_glcm_SumEntropy, wavelet.LLH_glcm_SumEntropy)

$$sum\begin{matrix} \\ \end{matrix}entropy=\sum_{k=2}^{2N_{g}} p_{x+y}(k)\log_{2}(p_{x+y}(k)+\varepsilon)$$

Sum Entropy is a sum of neighborhood intensity value differences.

1. **Dependence Entropy** **(DE)** (log.sigma.3.5.mm.3D_gldm_DependenceEntropy, log.sigma.2.5.mm.3D_gldm_DependenceEntropy, square_gldm_DependenceEntropy, log.sigma.4.5.mm.3D_gldm_DependenceEntropy, exponential_gldm_DependenceEntropy, log.sigma.1.5.mm.3D_gldm_DependenceEntropy, log.sigma.0.5.mm.3D_gldm_DependenceEntropy)

$$Dependence\begin{matrix} \\ \end{matrix}Entropy=-\sum_{i=1}^{N_{g}} \sum_{j=1}^{N_{d}} p(i,j)\log_{2}(p(i,j)+\varepsilon)$$

1. **Sum Squares** (log.sigma.0.5.mm.3D_glcm_SumSquares, log.sigma.2.5.mm.3D_glcm_SumSquares, log.sigma.3.5.mm.3D_glcm_SumSquares)

$$sum\begin{matrix} \\ \end{matrix}squares=\sum_{i=1}^{N_{g}} \sum_{j=1}^{N_{g}} (i-u_{x})^{2}p(i,j)$$

Sum of Squares or Variance is a measure in the distribution of neigboring intensity level pairs about the mean intensity level in the GLCM.

1. **Large Dependence Low Gray Level Emphasis  (LDLGLE)** (square_gldm_LargeDependenceLowGrayLevelEmphasis, exponential_gldm_LargeDependenceLowGrayLevelEmphasis, logarithm_gldm_LargeDependenceLowGrayLevelEmphasis)

$$LDLGLE=\frac{\sum_{i=1}^{N_{g}} \sum_{j=1}^{N_{d}} \frac{P(i,j)j^{2}}{i^{2}}}{N_{z}}$$

Measures the joint distribution of large dependence with lower gray-level values.

1. **Cluster Tendency** (log.sigma.0.5.mm.3D_glcm_ClusterTendency)

$$cluster\begin{matrix} \\ \end{matrix}tendency=\sum_{i=1}^{N_{g}} \sum_{j=1}^{N_{g}} (i+j-u_{x}-u_{y})^{2}p(i,j)$$

Cluster Tendency is a measure of groupings of voxels with similar gray-level values.

1. **Gray Level Variance (GLV)**  (log.sigma.0.5.mm.3D_glrlm_GrayLevelVariance, log.sigma.0.5.mm.3D_gldm_GrayLevelVariance, log.sigma.3.5.mm.3D_gldm_GrayLevelVariance, log.sigma.3.5.mm.3D_glrlm_GrayLevelVariance, log.sigma.2.5.mm.3D_gldm_GrayLevelVariance)

$$GLV=\sum_{i=1}^{N_{g}} \sum_{j=1}^{N_{r}} p(i,j|\theta)(i-u)^{2}$$

Here, $u=\sum_{i=1}^{N_{g}} \sum_{j=1}^{N_{r}} p(i,j|\theta)i$

GLV measures the variance in gray level intensity for the runs.

1. **Variance** (log.sigma.0.5.mm.3D_firstorder_Variance, log.sigma.3.5.mm.3D_firstorder_Variance, log.sigma.2.5.mm.3D_firstorder_Variance, exponential_firstorder_Variance)

$$\mathrm{var}iance=\frac{1}{N_{p}}\sum_{i=1}^{N_{p}} (X(i)-\overline{X})^{2}$$

Variance is the the mean of the squared distances of each intensity value from the Mean value. This is a measure of the spread of the distribution about the mean. By definition, $\mathrm{var}iance=\sigma^{2}$.

1. **Kurtosis** (log.sigma.2.5.mm.3D_firstorder_Kurtosis, log.sigma.0.5.mm.3D_firstorder_Kurtosis)

$$kurtosis=\frac{u_{4}}{\sigma^{4}}=\frac{\frac{1}{N_{p}}\sum_{i=1}^{N_{p}} (X(i)-\overline{X})^{4}}{(\frac{1}{N_{p}}\sum_{i=1}^{N_{p}} (X(i)-\overline{X})^{2})^{2}}$$

Where $u_{4}$ is the $4^{th}$ central moment.

Kurtosis is a measure of the ‘peakedness’ of the distribution of values in the image ROI. A higher kurtosis implies that the mass of the distribution is concentrated towards the tail(s) rather than towards the mean. A lower kurtosis implies the reverse: that the mass of the distribution is concentrated towards a spike near the Mean value.

Related links:

<https://en.wikipedia.org/wiki/Kurtosis>

1. **High Gray Level Emphasis  (HGLE)** (logarithm_gldm_HighGrayLevelEmphasis, logarithm_glrlm_ShortRunHighGrayLevelEmphasis, logarithm_glszm_SmallAreaHighGrayLevelEmphasis)

$$HGLE=\frac{\sum_{i=1}^{N_{g}} \sum_{j=1}^{N_{d}} P(i,j)i^{2}}{N_{z}}$$

Measures the distribution of the higher gray-level values, with a higher value indicating a greater concentration of high gray-level values in the image.

1. **Joint Average** (logarithm_glcm_JointAverage, log.sigma.2.5.mm.3D_glcm_JointAverage, log.sigma.3.5.mm.3D_glcm_JointAverage)

$$jo\mathrm{int}\begin{matrix} \\ \end{matrix}average=u_{x}=\sum_{i=1}^{N_{g}} \sum_{j=1}^{N_{g}} p(i,j)i$$

Returns the mean gray level intensity of the  i distribution.

1. **Sum Average** (logarithm_glcm_SumAverage, log.sigma.2.5.mm.3D_glcm_SumAverage, log.sigma.3.5.mm.3D_glcm_SumAverage)

$$sum\begin{matrix} \\ \end{matrix}average=\sum_{k=2}^{2N_{g}} p_{x+y}(k)k$$

Sum Average measures the relationship between occurrences of pairs with lower intensity values and occurrences of pairs with higher intensity values.

1. **High Gray Level Run Emphasis (HGLRE)** (logarithm_glrlm_HighGrayLevelRunEmphasis)

$$HGLRE=\frac{\sum_{i=1}^{N_{g}} \sum_{j=1}^{N_{r}} P(i,j|\theta)i^{2}}{N_{r}(\theta)}$$

HGLRE measures the distribution of the higher gray-level values, with a higher value indicating a greater concentration of high gray-level values in the image.

1. **High Gray Level Zone Emphasis**  **(HGLZE)** (logarithm_glszm_HighGrayLevelZoneEmphasis)

$$HGLZE=\frac{\sum_{i=1}^{N_{g}} \sum_{j=1}^{N_{s}} P(i,j)i^{2}}{N_{z}}$$

HGLZE measures the distribution of the higher gray-level values, with a higher value indicating a greater proportion of higher gray-level values and size zones in the image.

1. **Autocorrelation** (logarithm_glcm_Autocorrelation, log.sigma.2.5.mm.3D_glcm_Autocorrelation)

$$autocorrelation=\sum_{i=1}^{N_{g}} \sum_{j=1}^{N_{g}} p(i,j)ij$$

Autocorrelation is a measure of the magnitude of the fineness and coarseness of texture.

1. **Short Run Low Gray Level Emphasis (SRLGLE)** (log.sigma.3.5.mm.3D_glrlm_ShortRunLowGrayLevelEmphasis, logarithm_glrlm_ShortRunLowGrayLevelEmphasis, squareroot_glrlm_ShortRunLowGrayLevelEmphasis, log.sigma.2.5.mm.3D_glrlm_ShortRunLowGrayLevelEmphasis)

$$SRLGLE=\frac{\sum_{i=1}^{N_{g}} \sum_{j=1}^{N_{r}} \frac{P(i,j|\theta)}{i^{2}j^{2}}}{N_{r}(\theta)}$$

SRLGLE measures the joint distribution of shorter run lengths with lower gray-level values.

1. **Low Gray Level Emphasis (LGLE)** (log.sigma.3.5.mm.3D_gldm_LowGrayLevelEmphasis, logarithm_gldm_LowGrayLevelEmphasis, log.sigma.2.5.mm.3D_gldm_LowGrayLevelEmphasis, squareroot_gldm_LowGrayLevelEmphasis, log.sigma.4.5.mm.3D_gldm_LowGrayLevelEmphasis)

$$LGLE=\frac{\sum_{i=1}^{N_{g}} \sum_{j=1}^{N_{d}} \frac{P(i,j)}{i^{2}}}{N_{z}}$$

Measures the distribution of low gray-level values, with a higher value indicating a greater concentration of low gray-level values in the image.

1. **Gray Level Non-Uniformity Normalized  (GLNN)** (log.sigma.3.5.mm.3D_glrlm_GrayLevelNonUniformityNormalized, wavelet.LLH_glrlm_GrayLevelNonUniformityNormalized, log.sigma.0.5.mm.3D_glszm_GrayLevelNonUniformityNormalized)

$$GLNN=\frac{\sum_{i=1}^{N_{g}} (\sum_{j=1}^{N_{r}} P(i,j|\theta))^{2}}{N_{r}(\theta)^{2}}$$

GLNN measures the similarity of gray-level intensity values in the image, where a lower GLNN value correlates with a greater similarity in intensity values. This is the normalized version of the GLN formula.

1. **Surface Volume Ratio** (original_shape_SurfaceVolumeRatio)

$$surface\begin{matrix} \\ \end{matrix}to\begin{matrix} \\ \end{matrix}volume\begin{matrix} \\ \end{matrix}ratio=\frac{A}{V}$$

Here, a lower value indicates a more compact (sphere-like) shape. This feature is not dimensionless, and is therefore (partly) dependent on the volume of the ROI.

1. **Low Gray Level Run Emphasis  (LGLRE)** (logarithm_glrlm_LowGrayLevelRunEmphasis, squareroot_glrlm_LowGrayLevelRunEmphasis, log.sigma.2.5.mm.3D_glrlm_LowGrayLevelRunEmphasis, log.sigma.3.5.mm.3D_glrlm_LowGrayLevelRunEmphasis)

$$LGLRE=\frac{\sum_{i=1}^{N_{g}} \sum_{j=1}^{N_{r}} \frac{P(i,j|\theta)}{i^{2}}}{N_{r}(\theta)}$$

LGLRE measures the distribution of low gray-level values, with a higher value indicating a greater concentration of low gray-level values in the image.

1. **Low Gray Level Zone Emphasis  (LGLZE)** (logarithm_glszm_LowGrayLevelZoneEmphasis)

$$LGLZE=\frac{\sum_{i=1}^{N_{g}} \sum_{j=1}^{N_{s}} \frac{P(i,j)}{i^{2}}}{N_{z}}$$

LGLZE measures the distribution of lower gray-level size zones, with a higher value indicating a greater proportion of lower gray-level values and size zones in the image.

1. **Small Area Low Gray Level Emphasis (SALGLE)** (logarithm_glszm_SmallAreaLowGrayLevelEmphasis, wavelet.LLL_glszm_SmallAreaLowGrayLevelEmphasis)

$$SALGLE=\frac{\sum_{i=1}^{N_{g}} \sum_{j=1}^{N_{s}} \frac{P(i,j)}{i^{2}j^{2}}}{N_{z}}$$

SALGLE measures the proportion in the image of the joint distribution of smaller size zones with lower gray-level values.

1. **Minimum** (log.sigma.3.5.mm.3D_firstorder_Minimum)

$$\min imum=\min(X)$$

1. **Gray Level Non-Uniformity  (GLN)** (wavelet.HHH_glrlm_GrayLevelNonUniformity)

$$GLN=\frac{\sum_{i=1}^{N_{g}} (\sum_{j=1}^{N_{r}} P(i,j|\theta))^{2}}{N_{r}(\theta)}$$

GLN measures the similarity of gray-level intensity values in the image, where a lower GLN value correlates with a greater similarity in intensity values.

1. **Cluster Prominence** (log.sigma.0.5.mm.3D_glcm_ClusterProminence, log.sigma.3.5.mm.3D_glcm_ClusterProminence)

$$cluster\begin{matrix} \\ \end{matrix}pro\min ence=\sum_{i=1}^{N_{g}} \sum_{j=1}^{N_{g}} (i+j-u_{x}-u_{y}\text{）}^{4}p(i,j)$$

Cluster Prominence is a measure of the skewness and asymmetry of the GLCM. A higher values implies more asymmetry about the mean while a lower value indicates a peak near the mean value and less variation about the mean.

1. **Maximum** (logarithm_firstorder_Maximum)

$$\max imum=\max(X)$$

The maximum gray level intensity within the ROI.
